# Neonatal magnesium sulphate for neuroprotection: A systematic review and meta‐analysis

**DOI:** 10.1111/dmcn.15899

**Published:** 2024-03-11

**Authors:** Emily Shepherd, Tasneem Karim, Sarah McIntyre, Shona Goldsmith, Amy Keir, Nadia Badawi, Rod W. Hunt, Robert Galinsky

**Affiliations:** ^1^ Women and Kids Theme, South Australian Health and Medical Research Institute Adelaide South Australia Australia; ^2^ Adelaide Medical School The University of Adelaide Adelaide South Australia Australia; ^3^ Cerebral Palsy Alliance Research Institute, Sydney Medical School The University of Sydney Sydney New South Wales Australia; ^4^ Grace Centre for Newborn Intensive Care The Children's Hospital Sydney New South Wales Australia; ^5^ Department of Paediatrics Monash University Melbourne Victoria Australia; ^6^ Monash Newborn Monash Children's Hospital Melbourne Victoria Australia; ^7^ The Ritchie Centre, Hudson Institute of Medical Research Melbourne Victoria Australia; ^8^ Department of Obstetrics and Gynaecology Monash University Melbourne Victoria Australia

## Abstract

**Aim:**

To review the evidence of the effects of neonatal magnesium sulphate for neuroprotection in perinatal asphyxia and hypoxic‐ischaemic encephalopathy (HIE).

**Method:**

This was a systematic review of randomized controlled trials (RCTs) (with meta‐analysis) and non‐RCTs assessing magnesium sulphate for treating perinatal asphyxia and HIE at 35 weeks or more gestation (primary outcomes: neonatal death and death or long‐term major neurodevelopmental disability).

**Results:**

Twenty‐five RCTs (2099 infants) and four non‐RCTs (871 infants) were included, 23 in low‐ and middle‐income countries (LMICs). In RCTs, reductions in neonatal death with magnesium sulphate versus placebo or no treatment (risk ratio [RR] = 0.68; 95% confidence interval [CI] = 0.53–0.86; 13 RCTs), and magnesium sulphate with melatonin versus melatonin alone (RR = 0.74; 95% CI = 0.58–0.95; one RCT) were observed. No difference in neonatal death was seen for magnesium sulphate with therapeutic hypothermia versus therapeutic hypothermia alone (RR = 0.66, 95% CI = 0.34–1.26; three RCTs), or magnesium sulphate versus phenobarbital (RR = 3.00; 95% CI = 0.86–10.46; one RCT). No reduction in death or long‐term neurodevelopmental disability (RR = 0.52; 95% CI = 0.14–1.89; one RCT) but reductions in several short‐term adverse outcomes were observed with magnesium sulphate. Evidence was low‐ to very‐low certainty because of risk of bias and imprecision.

**Interpretation:**

Given the uncertainty of the current evidence, further robust neonatal magnesium sulphate research is justified. This may include high‐quality studies to determine stand‐alone effects in LMICs and effects with and after therapeutic hypothermia in high‐income countries.

## Graphical abstract

- Magnesium sulphate (MgSO4) in perinatal asphyxia/hypoxic‐ischaemic encephalopathy may reduce neonatal death.
- MgSO4 and therapeutic hypothermia (TH) may be no more effective than TH.
- Effects of MgSO4 on death or long‐term neurodevelopmental disability are very uncertain.
- Evidence available is limited and of low‐ to very‐low certainty.

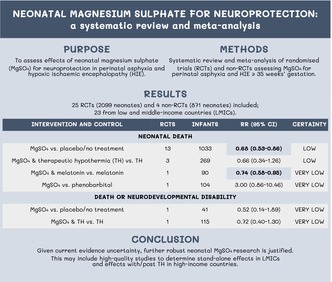

**Abbreviations**
HIC: high‐income country
HIE: hypoxic‐ischaemic encephalopathy
LMIC: low‐ and middle‐income country
RCT: randomized controlled trial

What this paper adds

- Magnesium sulphate in perinatal asphyxia and hypoxic‐ischaemic encephalopathy may reduce neonatal death.
- Magnesium sulphate and therapeutic hypothermia may be no more effective than therapeutic hypothermia.
- The effects of magnesium sulphate on death or long‐term neurodevelopmental disability are very uncertain.
- Current evidence is limited and uncertain.

Globally, approximately 2.4 million neonatal deaths (in the first 28 days of postnatal life) occur annually.^1^ One‐third of deaths are on the first day of life and three‐quarters within the first week; many are associated with preventable or treatable conditions, including hypoxia‐ischaemia during the birth process (before, during, or after birth, often referred to as ‘perinatal asphyxia’).^1^ Neonatal encephalopathy is a complex neurological syndrome in infants born late preterm and at term, who demonstrate difficulty initiating and maintaining respiration, depression of consciousness, tone, or reflexes, and seizures.^2^ It is associated with a high risk of death. For surviving infants, it is a strong predictor of long‐term neurodevelopmental disability.^2^ The subset of neonatal encephalopathy with clear evidence of intrapartum asphyxia has traditionally been referred to as ‘hypoxic‐ischaemic encephalopathy’ (HIE).^2^ It is estimated that 8.5 infants per 1000 live births develop neonatal encephalopathy associated with intrapartum events; 96% of these infants are born in low‐ and middle‐income countries (LMICs).^3^

Currently, therapeutic hypothermia, or ‘cooling’, is the only evidence‐based therapy shown to decrease infant death or major long‐term neurodevelopmental disability in early neonatal encephalopathy of suspected hypoxic‐ischaemic origin.^4^ However, not all treated infants benefit. The number needed to treat to prevent one infant dying or developing major long‐term neurodevelopmental disability is 7 (95% confidence interval [CI] = 5–10; eight randomized controlled trials [RCTs], 1344 infants).^4^ Recent research efforts focused on neuroprotective adjuvants to therapeutic hypothermia, such as erythropoietin, have not demonstrated improved outcomes.^5^ Evidence supporting the efficacy of therapeutic hypothermia specifically in LMICs is also uncertain, with recent RCTs and systematic reviews demonstrating conflicting findings.^6^, ^7^, ^8^ There is thus an urgent, unmet need for alternative and adjuvant therapies in LMICs and high‐income countries (HICs) respectively.

Magnesium sulphate is a common therapy in perinatal care, with long‐standing use for preventing and treating eclampsia,^9^, ^10^ tocolysis,^11^ and in the last decade, for preterm fetal neuroprotection.^12^ While the exact neuroprotective mechanism(s) are unclear, recent advances propose the modulation of brain excitotoxic and inflammatory pathways,^13^, ^14^, ^15^, ^16^ both of which are implicated in neonatal encephalopathy pathophysiology. To date, preclinical studies assessing magnesium sulphate in term‐equivalent models of perinatal hypoxia‐ischaemia (alone and in combination with therapeutic hypothermia) have demonstrated inconsistent, arguably unconvincing, evidence of benefit.^17^, ^18^ Despite this, clinical studies, including RCTs, continue, probably because of the relative low cost, widespread availability, and a favourable safety profile of magnesium sulphate (of consequence in LMICs).^19^, ^20^

The most recent systematic review evaluating magnesium sulphate for infants with HIE was published in 2013.^21^ Magnesium sulphate was not associated with differences in the primary outcome (death or moderate‐to‐severe neurodevelopmental disability), or secondary outcome, death. A reduction in an ‘unfavourable short‐term composite outcome’ (survival with abnormalities in any of the following: neurodevelopmental exam, neuroimaging, or neurophysiological studies) was observed.^21^ As a decade has passed since the last comprehensive literature search, and therapies in LMICs are limited, an update on this important topic is timely. This systematic review and meta‐analysis aimed to determine the effects of neonatal magnesium sulphate in perinatal asphyxia and HIE, on death and long‐term neurodevelopmental disability.

## METHOD

This systematic review adhered to the Preferred Reporting Items for Systematic Reviews and Meta‐Analyses (PRISMA) guidelines.^22^ A protocol was developed and registered with the International Prospective Register of Systematic Reviews (PROSPERO) on 25th July 2022 (registration no. CRD42022346545) before commencement.

### Eligibility criteria

#### Studies

To provide a more complete picture of effectiveness and harms, RCTs and quasi‐RCTs, as well as non‐randomized controlled studies (non‐randomized trials, cohort studies, or case–control studies) were eligible. We excluded cross‐sectional studies, case series, and case reports. We included studies available as abstracts only along with full‐text publications.

#### Participants

Infants with evidence of perinatal asphyxia and HIE born at 35 weeks' gestation or later were eligible, irrespective of the criteria or definitions used.

#### Interventions and comparators

We included neonatal magnesium sulphate for perinatal asphyxia and HIE, regardless of timing, route of administration, dose, and duration. We excluded magnesium sulphate for persistent pulmonary hypertension of the newborn infant. We included magnesium sulphate compared with no treatment, placebo, standard care (e.g. therapeutic hypothermia), or alternative treatment(s). We included magnesium sulphate in combination with therapeutic hypothermia or alternative neuroprotective agents.

#### Outcomes

The primary outcomes were neonatal death and the composite outcome of neonatal death or long‐term major neurodevelopmental disability.

Secondary outcomes included a long‐term major neurodevelopmental disability composite, individual components of long‐term major neurodevelopmental disability, additional short‐term predictors of neurological outcome, and potential adverse effects of magnesium sulphate.

To encompass all relevant data, we included outcomes as defined and reported by the study authors.

### Search methods

Comprehensive searches of the databases CINAHL, Cochrane Library, Embase, MEDLINE, Scopus, and Web of Science were undertaken from their inceptions to 5th April 2023, using combinations of controlled vocabulary (such as medical subject heading terms) and free‐text words, guided by our PICO (population of interest, intervention, control, outcome) parameters. No date or language restrictions were applied. We searched Google Scholar and Google using free‐text words. The reference lists of eligible articles and reviews were checked. Details of the database search strategies are available in Appendix S1.

### Data collection and analysis

#### Study selection

Retrieved studies were exported to EndNote X9 (Clarivate, Philadelphia, PA, USA),^23^ before being uploaded into Covidence (Melbourne, Victoria, Australia)^24^ for deduplication and screening. After screening all titles and abstracts, we assessed full‐text articles for studies that appeared to meet the inclusion criteria. Each stage of screening was carried out by two reviewers (ES and TK) and discrepancies were resolved through discussion or in consultation with a third reviewer (SM or RG).

#### Data extraction

For the studies included in the review, data were extracted using a standardized form, including information regarding design, participants, magnesium sulphate regimen(s), comparator(s), outcomes reported, relevant results, and risk of bias. Extraction was carried out by two reviewers (ES and TK, SG, SM, or RG); discrepancies were resolved through discussion.

#### Quality assessment

Quality appraisal of RCTs was undertaken using the Cochrane Handbook for Systematic Reviews of Interventions guidance.^25^ Quality assessment of non‐randomized studies was guided by the RTI Item Bank.^26^

#### Data synthesis

Statistical analyses for RCTs were performed using Review Manager v5.4 (Cochrane Library, John Wiley & Sons, Hoboken, NJ, USA).^27^ Quantitative data were presented as risk ratios (RRs) for dichotomous outcomes and mean differences (MDs) for continuous outcomes, with 95% CIs. Where there were sufficient data, pooled estimates were calculated, first using a fixed‐effects meta‐analysis (Mantel–Haenszel method). Where there was substantial statistical heterogeneity (where *I* ^2^ was >30% and either T^2^ was >0 or the *p*‐value was low [<0.10] in the *χ* ^2^ test), pooled estimates were calculated using a random‐effects meta‐analysis.

For non‐randomized studies, we presented effect estimates (without pooling) where possible with 95% CIs, *p*‐values only, or percentages (rates) in tabular format, and the results were summarized narratively.

#### Subgroup and sensitivity analyses

Expected sources of heterogeneity were explored through prespecified subgroup analyses for the primary outcome—neonatal death—considering the characteristics of the participants (gestational age, HIE severity at inclusion, economic setting) and treatment (timing, dose). Because of the paucity of data, we were unable to conduct other prespecified subgroup analyses for the second primary outcome, that is, death or major long‐term neurodevelopmental disability, nor for participant (presence of infection) and treatment (route of administration) characteristics. We assessed subgroup differences using interaction tests and reported the *χ* ^2^ statistic, *p*‐value, and interaction test *I* ^2^ value. We conducted non‐prespecified sensitivity analyses, restricting primary outcome analyses to RCTs considered at low risk of bias overall.

#### Assessment of the certainty of the evidence

Certainty of the evidence for the primary outcomes was appraised using the Grading of Recommendations, Assessment, Development and Evaluations (GRADE) approach.^28^ Evidence was classified as very low, low, moderate, or high considering the following domains: study limitations, consistency, directness, imprecision, and publication bias.^28^ Certainty of evidence was assessed by two reviewers (ES and TK); discrepancies were resolved through discussion.

## RESULTS

### Study selection

Database searching identified 1801 records; after duplicate removal, there were 1074 records. Title and abstract screening identified 76 reports for full‐text screening, of which we excluded 52. Other searching identified a further 13 records to screen; we excluded two. Therefore, we included 29 studies (reported across 35 references).^29^, ^30^, ^31^, ^32^, ^33^, ^34^, ^35^, ^36^, ^37^, ^38^, ^39^, ^40^, ^41^, ^42^, ^43^, ^44^, ^45^, ^46^, ^47^, ^48^, ^49^, ^50^, ^51^, ^52^, ^53^, ^54^, ^55^, ^56^, ^57^, ^58^, ^59^, ^60^, ^61^, ^62^, ^63^ A study flow diagram is shown in Figure S1; Appendix S2 lists the records excluded at the full‐text screening.

### Study characteristics

Of the 29 studies included, 25 were RCTs,^29^, ^30^, ^31^, ^32^, ^33^, ^34^, ^35^, ^36^, ^37^, ^38^, ^39^, ^40^, ^41^, ^42^, ^43^, ^44^, ^45^, ^46^, ^47^, ^49^, ^50^, ^51^, ^53^, ^54^, ^55^, ^56^, ^57^, ^58^, ^59^, ^60^, ^61^ and four were non‐RCTs (two were non‐randomized trials and two were non‐concurrent cohort studies);^48^, ^52^, ^62^, ^63^ three were available as abstracts only.^33^, ^40^, ^50^ Twenty‐three studies were conducted exclusively in LMICs (nine in India;^31^, ^32^, ^35^, ^46^, ^47^, ^51^, ^58^, ^60^, ^62^ eight in Pakistan;^30^, ^42^, ^43^, ^49^, ^56^, ^57^, ^59^, ^61^ three in Egypt;^29^, ^33^, ^44^ one each in Bangladesh,^40^ Nigeria,^52^ and Albania^50^); five in HICs (two in Poland;^39^, ^63^ one each in the Netherlands^36^ and Japan;^41^ one across five European centres^48^); and one across mixed settings (Qatar, Turkey, Saudi Arabia, Egypt, Malaysia, and the United Arab Emirates^55^). Where reported, study commencement dates ranged from 1995^36^ to 2021.^43^, ^57^ Twelve studies (41.4%) were published in the last 5 years.^29^, ^30^, ^33^, ^39^, ^42^, ^43^, ^47^, ^51^, ^52^, ^57^, ^59^, ^61^

There were 2099 infants randomized in the 25 RCTs,^29^, ^30^, ^31^, ^32^, ^33^, ^34^, ^35^, ^36^, ^37^, ^38^, ^39^, ^40^, ^41^, ^42^, ^43^, ^44^, ^45^, ^46^, ^47^, ^49^, ^50^, ^51^, ^53^, ^54^, ^55^, ^56^, ^57^, ^58^, ^59^, ^60^, ^61^ and 871 infants in the non‐randomized studies.^48^, ^52^, ^62^, ^63^ The number of infants per RCT ranged from 22^36^ to 260.^30^ Infants in the studies varied substantially according to gestational age, criteria for asphyxia, and presence of HIE (including severity). Magnesium sulphate regimens also varied across studies. In 24 RCTs, an initial dose of 250 mg/kg was given intravenously (commonly within 30 minutes to 6 hours after birth), followed by no additional treatment (one RCT^44^), or two further doses of either 125 mg/kg (three RCTs^35^, ^36^, ^58^) or 250 mg/kg (20 RCTs^29^, ^30^, ^31^, ^32^, ^39^, ^40^, ^41^, ^42^, ^43^, ^46^, ^47^, ^49^, ^50^, ^51^, ^55^, ^56^, ^57^, ^59^, ^60^, ^61^) intravenously, 24 hours and 48 hours later. In one RCT, the regimen was unclear.^33^ Detailed descriptions of the individual study characteristics are provided in Tables S1 (RCTs) and S2 (non‐randomized studies).

### Risk of bias

The methodological quality of the 25 RCTs varied considerably.^29^, ^30^, ^31^, ^32^, ^33^, ^34^, ^35^, ^36^, ^37^, ^38^, ^39^, ^40^, ^41^, ^42^, ^43^, ^44^, ^45^, ^46^, ^47^, ^49^, ^50^, ^51^, ^53^, ^54^, ^55^, ^56^, ^57^, ^58^, ^59^, ^60^, ^61^ Considering selection bias, only three were at low risk.^42^, ^47^, ^55^ Four RCTs were at low risk of performance and detection bias;^31^, ^36^, ^44^, ^55^ 15 were at low risk of attrition bias;^29^, ^30^, ^31^, ^35^, ^36^, ^41^, ^43^, ^44^, ^46^, ^49^, ^55^, ^56^, ^57^, ^58^, ^59^ and only one was at low risk of reporting bias.^47^ The overall risk of bias across RCTs is presented in Figure S2; the domain‐level risk of bias for individual RCTs is presented in Figure S3 and Table S3.

The four non‐randomized studies had an overall high risk of bias.^48^, ^52^, ^62^, ^63^ Common concerns were related to the potential for confounding, detection bias, and performance bias. Domain‐level judgements for the individual studies are presented in Table S4.

### Evidence from RCTs

Intervention (magnesium sulphate) and comparator groups differed across RCTs. Thus, the results have been presented under four comparisons.

#### Comparison 1: magnesium sulphate versus placebo or no treatment

This comparison included 18 RCTs;^30^, ^31^, ^32^, ^35^, ^36^, ^40^, ^41^, ^42^, ^44^, ^46^, ^49^, ^50^, ^51^, ^56^, ^58^, ^59^, ^60^, ^61^ 16 were conducted in LMICs^30^, ^31^, ^32^, ^35^, ^40^, ^42^, ^44^, ^46^, ^49^, ^50^, ^51^, ^56^, ^58^, ^59^, ^60^, ^61^ (see Table 1 for the Comparison 1 effect estimates, Table 2 for the GRADE Evidence Profile for the primary outcomes, and Appendix S3 for the forest plots [and funnel plot]).

**TABLE 1:** Results for Comparison 1: magnesium sulphate versus placebo or no treatment.

Outcome and subgroup	RCTs, *n*	Infants, *n*	Methods (*I* ^2^)	RR (95% CI)
Neonatal death	13	1033	F (22%)	**0.68 (0.53–0.86)**
Death or neurodevelopmental disability at 12 months	1	41	F (NA)	0.52 (0.14–1.89)
HIE				
Stage I	1	80	F (NA)	1.25 (0.55–2.84)
Stage II	1	50	F (NA)	0.63 (0.32–1.21)
Stage III	1	80	F (NA)	1.00 (0.15–6.76)
Any	1	80	F (NA)	0.85 (0.59–1.21)
Seizures	8	392	F (6%)	**0.79 (0.67–0.94)**
Refractory seizures	1	40	F (NA)	0.50 (0.05–5.08)
Seizure control				
Control of fits	1	260	F (NA)	**1.54 (1.28–1.86)**
With one anticonvulsant	1	52	F (NA)	**1.30 (1.02–1.64)**
Requiring more than one anticonvulsant	1	52	F (NA)	0.15 (0.02–1.17)
Use of anticonvulsant drugs				
0	1	22	R (NA)	0.58 (0.07–4.72)
1	2	249	R (0%)	**2.49 (1.89–3.28)**
2	2	249	R (6%)	**0.22 (0.09–0.50)**
3	2	249	R (53%)	0.52 (0.11–2.57)
4	2	249	R (0%)	**0.26 (0.07–0.99)**
Number of seizures				
1	2	307	R (82%)	3.16 (0.76–13.18)
2–5	1	227	R (NA)	0.84 (0.66–1.07)
Multiple	1	80	R (NA)	0.96 (0.65–1.41)
More than 5	1	227	R (NA)	**0.27 (0.10–0.69)**
Duration of seizures				
Less than 48 hours	2	132	R (81%)	2.27 (0.42–12.25)
More than 48 hours	1	80	R (NA)	0.79 (0.47–1.32)
Duration of seizures (days)	3	147	F (27%)	**MD: −0.84 (−1.19 to − 0.49)**
Anticonvulsant required at discharge	2	80	F (1%)	1.23 (0.34–4.44)
EEG abnormalities				
Any	3	101	F (0%)	0.59 (0.35–1.01)
Low‐voltage	1	33	F (NA)	0.56 (0.16–1.99)
Focal periodic lateralized epileptiform discharges	1	33	F (NA)	0.38 (0.08–1.67)
Suppression of aEEG background pattern before administration (3 hours) to 12 hours	1	21	F (NA)	**3.25 (1.11–9.48)**
CUS subcortical echodensities within 4 hours of birth	1	22	F (NA)	1.75 (0.59–5.15)
CUS abnormalities at day 14	1	32	F (NA)	0.83 (0.32–2.18)
CUS abnormalities at discharge	1	56	F (NA)	0.71 (0.42–1.19)
CT abnormalities at day 14				
Any	4	171	F (0%)	**0.45 (0.29–0.72)**
Diffuse hypodensities	1	32	F (NA)	0.33 (0.08–1.41)
Multifocal areas of hypodensity	1	32	F (NA)	2.00 (0.20–19.91)
Focal area of hypodensity	1	32	F (NA)	0.67 (0.13–3.47)
Diffuse cortical injury/cortical injury	2	69	F (0%)	0.96 (0.26–3.55)
Injury to both cortex and deep grey structure/injury to both cortical and subcortical areas	2	69	F (19%)	0.44 (0.10–1.85)
Injury to basal ganglia and thalamus/basal ganglia injury	2	69	F (24%)	0.33 (0.08–1.26)
CT abnormalities at discharge	1	66	F (NA)	**0.31 (0.13–0.75)**
Normal neuroimaging at discharge	1	67	F (NA)	1.28 (0.93–1.75)
Duration of recovery from neurological abnormalities (days)	1	120	F (NA)	**MD: −1.60 (−2.08 to − 1.12)**
Recovery from abnormal neurological examination within 4 days	1	105	F (NA)	**1.58 (1.19–2.11)**
Good short‐term neonatal outcome	4	138	F (0%)	**1.83 (1.29–2.60)**
Composite neonatal adverse outcome (including death)	2	72	F (3%)	**0.61 (0.39–0.95)**
Normal neuromotor tone (Amiel‐Tison criteria) at discharge	1	67	F (NA)	**1.33 (1.03–1.72)**
Neurological status normal or improved at discharge	3	213	F (0%)	**1.57 (1.27–1.95)**
Neurological abnormalities at discharge	5	207	F (0%)	**0.46 (0.32–0.67)**
Final outcome – discharge	1	227	F (NA)	**1.49 (1.22–1.82)**
Assisted ventilation	3	193	F (0%)	0.87 (0.65–1.16)
Mechanical ventilation at 72 hours	1	47	F (NA)	2.09 (0.73–5.99)
Need for oxygen				
Oxygen required	1	227	F (NA)	**0.84 (0.73–0.96)**
Need for oxygen at 72 hours	1	47	F (NA)	1.17 (0.55–2.51)
Duration of assisted ventilation (hours)	1	33	F (NA)	MD: −31.00 (−164.76 to 102.76)
Apnoea at 72 hours	2	127	R (58%)	1.54 (0.15–15.52)
Shock and use of inotropes				
Use of inotropes at 72 hours	1	47	F (NA)	1.04 (0.47–2.31)
Shock	2	347	F (0%)	**0.64 (0.46–0.87)**
Number of inotropes required				
Dobutamine	1	227	F (NA)	0.96 (0.46–1.97)
Dobutamine and dopamine	1	227	F (NA)	0.69 (0.36–1.34)
Dobutamine and dopamine and adrenaline	1	227	F (NA)	**0.32 (0.13–0.77)**
Hypotension requiring pressor support	1	40	F (NA)	1.40 (0.53–3.68)
PPHN	1	40	F (NA)	0.86 (0.35–2.10)
Disseminated intravascular coagulation	1	40	F (NA)	1.00 (0.16–6.42)
Creatine phosphokinase (U/l)	2	73	F (0%)	MD: −103.17 (−1235.05 to 1028.71)
Lactate dehydrogenase (U/l)	2	73	F (12%)	MD: 37.19 (−841.45 to 915.82)
CSF concentrations				
Glutamate at 72 hours (μmol/l)	1	36	F (NA)	MD: 0.32 (−10.19 to 10.83)
Glutamate from baseline to 72 hours (μmol/l)	1	36	F (NA)	MD: 6.11 (−4.76 to 16.98)
Aspartate at 72 hours (μmol/l)	1	36	F (NA)	MD: 0.26 (−1.02 to 1.54)
Aspartate from baseline to 72 hours (μmol/l)	1	36	F (NA)	MD: 0.25 (−1.48 to 1.98)
TNF‐α from baseline to 72 hours (pg/ml)	1	36	F (NA)	MD: −130.00 (−321.21 to 61.21)
Oliguria	1	40	F (NA)	1.00 (0.34–2.93)
Renal failure	1	40	F (NA)	1.09 (0.64–1.86)
Hepatic dysfunction	1	40	F (NA)	1.00 (0.23–4.37)
Acute kidney injury	1	120	F (NA)	0.50 (0.20–1.25)
Antibiotics required	1	227	F (NA)	1.02 (0.96–1.09)
Duration for initiation of feeding (days)				
Feeds	1	62	F (NA)	**MD: −0.94 (−1.33 to − 0.54)**
Nasogastric tube feeding	1	120	F (NA)	**MD: −0.88 (−1.28 to − 0.48)**
Paladai feeding	1	120	F (NA)	**MD: −2.10 (−2.72 to − 1.48)**
Direct breastfeeding	1	120	F (NA)	**MD: −1.40 (−1.91 to − 0.89)**
Established feeding at day 7	2	72	F (0%)	**1.75 (1.04–2.95)**
Nasogastric feed on day 14 of life	1	200	F (NA)	**0.33 (0.20–0.54)**
Established oral feeding at day 14	1	33	F (NA)	1.25 (0.74–2.13)
Oral feeding at discharge	9	815	F (19%)	**1.79 (1.58–2.04)**
OGT feeding at discharge	1	227	F (NA)	0.70 (0.29–1.67)
Duration of stay (days)	1	62	F (NA)	**MD: −1.13 (−1.92 to − 0.33)**
Normal development at 6 months	2	86	F (0%)	1.13 (0.88–1.45)
Developmental delay at 6 months	2	86	F (0%)	0.69 (0.33–1.45)
Occipitofrontal circumference at 6 months (cm)	1	32	F (NA)	MD: −0.02 (−0.83 to 0.79)
Head circumference at 12 months (cm)	1	41	F (NA)	MD: 0.30 (−0.01 to 0.61)
Weight at 12 months (kg)	1	41	F (NA)	**MD: −0.50 (−0.59 to − 0.41)**
Length at 12 months (cm)	1	41	F (NA)	**MD: −1.20 (−1.52 to − 0.88)**
Antiepileptic drugs for seizures at 12 months	1	41	F (NA)	0.58 (0.11–3.09)
Developmental delay at 12 months	1	41	F (NA)	0.52 (0.14–1.89)
Normal development at 12 months	1	41	F (NA)	1.17 (0.85–1.61)
Abnormal neuromotor tone at 12 months	1	41	F (NA)	0.65 (0.17–2.54)
Griffiths developmental quotient <85 (among survivors) at 24 months	1	12	F (NA)	0.36 (0.02–6.12)
Cerebral palsy (among survivors) at 24 months	1	12	F (NA)	0.36 (0.02–6.12)

*Note*: Statistically significant effect estimates are shown in bold. The test for heterogeneity is represented by the *I* ^2^ statistic; when *I* ^2^ > 30%, summary estimates were calculated using a random‐effects meta‐analysis.

Abbreviations: aEEG, amplitude‐integrated electroencephalogram; CI, confidence interval; CSF, cerebrospinal fluid; CT, computed tomography; CUS, cranial ultrasound; EEG, electroencephalogram; F, fixed effect; HIE, hypoxic‐ischaemic encephalopathy; MD, mean difference; NA, not applicable; OGT, orogastric tube; PPHN, persistent pulmonary hypertension of the newborn; R, random effects; RCT, randomized controlled trial; RR, risk ratio; TNF‐α, tumour necrosis factor‐alpha.

**TABLE 2:** GRADE Evidence Profile for the primary outcomes (certainty assessment)

Certainty assessment							No. of patients on		Effect of magnesium sulphate		Certainty
No. of studies	Design	Risk of bias	Inconsistency	Indirectness	Imprecision	Other considerations	Magnesium sulphate	Placebo or no treatment	RR (95% CI)	Absolute (95% CI)	
*Comparison 1: magnesium sulphate versus placebo or no treatment*											
Neonatal death											
13	RCTs	Very serious^a^	Not serious	Not serious^b^	Not serious	11/13 in LMICs	84/516 (16.3%)	126/517 (24.4%)	**0.68 (0.53–0.86)**	165 fewer per 1000 (from 129 fewer to 209 fewer)	⊕ ⊕ ⊝⊝ Low
Death or neurodevelopmental disability at 12 months											
1	RCT	Serious^c^	NA	Not serious^b^	Very serious^d^	LMIC	3/22 (13.6%)	5/19 (26.3%)	0.52 (0.14–1.89)	136 fewer per 1000 (from 36 fewer to 497 more)	⊕⊝⊝⊝ Very low
*Comparison 2: magnesium sulphate and therapeutic hypothermia versus therapeutic hypothermia alone*											
Neonatal death											
3	RCTs	Serious^c^	Not serious	Not serious^b^	Serious^e^	1/3 in LMICs (one mixed)	13/134 (9.7%)	20/135 (14.8%)	0.66 (0.34–1.26)	97 fewer per 1000 (from 50 fewer to 186 more)	⊕ ⊕ ⊝⊝ Low
Neonatal death or DASII score <70 at 12 months											
1	RCT	Serious^c^	NA	Not serious^b^	Very serious^d^	LMIC	14/58 (24.1%)	19/57 (33.3%)	0.72 (0.40–1.30)	240 fewer per 1000 (from 133 fewer to 433 more)	⊕⊝⊝⊝ Very low
*Comparison 3: magnesium sulphate and melatonin versus melatonin alone*											
Neonatal death											
1	RCT	Very serious^a^	NA	Serious^f^	Not serious	LMIC	29/45 (64.4%)	39/45 (86.7%)	**0.74 (0.58–0.95)**	641 fewer per 1000 (from 502 fewer to 823 fewer)	⊕⊝⊝⊝ Very low
*Comparison 4: magnesium sulphate versus phenobarbital*											
Neonatal death											
1	RCT	Very serious^a^	NA	Serious^g^	Very serious^d^	LMIC	9/52 (17.3%)	3/52 (5.8%)	3.00 (0.86–10.46)	173 per 1000 more (from 49 fewer to 603 more)	⊕⊝⊝⊝ Very low

*Note*: Statistically significant effect estimates are shown in bold.

Abbreviations: CI, confidence interval; DASII, Developmental Assessment Scales for Indian Infants; GRADE, Grading of Recommendations Assessment, Development and Evaluation; LMIC, low‐ and middle‐income country; NA, not applicable; RCT, randomized controlled trial; RR, risk ratio.

^a^ Contributing trial(s) at risk of bias, including unclear risk of selection bias (among other concerns).

^b^ Trials predominately conducted in LMICs; not downgraded for indirectness but noted as an important consideration.

^c^ Contributing trial(s) at risk of bias.

^d^ Very wide CIs crossing the line of no effect.

^e^ Wide CIs crossing the line of no effect.

^f^ Notably high neonatal death rates reported in the trial population.

^g^ Trial excluded infants with unstable vital signs (Apgar score <5 at 5 minutes).

##### Primary outcomes

**FIGURE 1:**
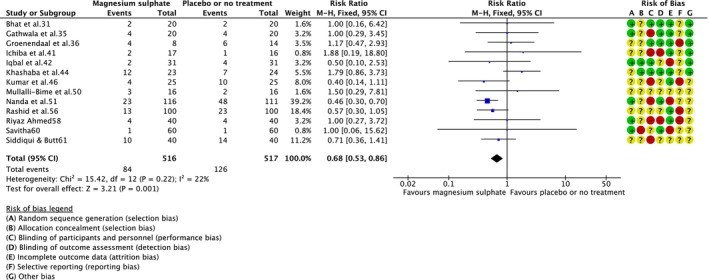
Effect of magnesium sulphate versus placebo or no treatment on neonatal death. Abbreviation: CI, confidence interval.

**FIGURE 2:**
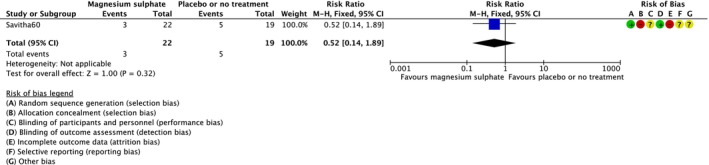
Effect of magnesium sulphate versus placebo or no treatment on death or neurodevelopmental disability at 12 months. Abbreviation: CI, confidence interval.

Treatment with magnesium sulphate versus placebo or no treatment reduced the risk of neonatal death (RR = 0.68; 95% CI = 0.53–0.86; 13 RCTs,^31^, ^35^, ^36^, ^41^, ^42^, ^44^, ^46^, ^50^, ^51^, ^56^, ^58^, ^60^, ^61^ 1033 infants; low‐certainty evidence [Figure 1]). No obvious asymmetry raising concern about potential publication bias was observed on visual assessment of the funnel plot (Appendix S3). In a sensitivity analysis restricted to the two RCTs and overall low risk of bias,^31^, ^42^ no difference between magnesium sulphate and placebo or no treatment was observed (RR = 0.67; 95% CI = 0.20–2.23; 102 infants). There was no clear difference in death or neurodevelopmental disability at 12 months (outcome not clearly defined in the RCT) with magnesium sulphate versus placebo or no treatment (RR = 0.52; 95% CI = 0.14–1.89; one RCT,^60^ 41 infants; very‐low‐certainty evidence) (Figure 2); sensitivity analysis was not possible (one included RCT^60^ was not at low risk of bias).

Subgroup analyses for neonatal death indicated possible differential treatment effects according to gestational age (*χ* ^2^ = 7.30, *p* = 0.007, *I* ^2^ = 86.3%): potential reduction in neonatal death was observed in the meta‐analysis of three RCTs with both infants born late preterm and at term (vs 10 RCTs with infants born at term only); HIE severity at inclusion (*χ* ^2^ = 10.36, *p* = 0.001, *I* ^2^ = 90.3%): potential reduction in neonatal death seen in the meta‐analysis of five RCTs that restricted inclusion to infants with moderate or severe HIE (vs eight RCTs with no restriction); timing of magnesium sulphate administration (*χ* ^2^ = 8.23, *p* = 0.04, *I* ^2^ = 63.5%): potential reduction in neonatal death observed in the meta‐analysis of seven RCTs initiating magnesium sulphate within 6 hours of birth (vs within 24 hours in four RCTs or beyond 24 hours in one RCT); magnesium sulphate dose (*χ* ^2^ = 10.19, *p* = 0.006, *I* ^2^ = 80.4%): potential reduction in neonatal death seen in the meta‐analysis of nine RCTs using three doses of 250 mg/kg magnesium sulphate at 24‐hour intervals (vs one dose of 250 mg/kg only in one RCT, or one dose of 250 mg/kg and two doses of 125 mg/kg in three RCTs).

The subgroup interaction test did not indicate a clear differential treatment effect according to setting (LMICs vs HICs) (*χ* ^2^ = 2.24, *p* = 0.13, *I* ^2^ = 55.3%) (see Table S5 for the subgroup effect estimates).

##### Secondary outcomes

Infants treated with magnesium sulphate versus placebo or no treatment were at reduced risk of seizures (eight RCTs,^31^, ^35^, ^36^, ^41^, ^46^, ^58^, ^60^, ^61^ 392 infants), requiring two or four anticonvulsant drugs (both two RCTs,^36^, ^51^ 249 infants), having more than five seizures (one RCT,^51^ 227 infants), having any abnormalities on computed tomography at day 14 (four RCTs,^31^, ^35^, ^41^, ^49^ 171 infants) and on day of discharge (one RCT,^59^ 66 infants), a ‘composite neonatal adverse outcome’ (variously defined, two RCTs,^36^, ^46^ 72 infants), neurological abnormalities at discharge (variously defined, five RCTs,^31^, ^40^, ^46^, ^50^, ^59^ 207 infants), requiring oxygen (one RCT,^51^ 227 infants), experiencing ‘shock’ (two RCTs,^51^, ^60^ 347 infants), requiring three inotropes (dobutamine, dopamine, adrenaline) (one RCT,^51^ 227 infants), and requiring nasogastric feeds on day 14 (Table 1).

For infants, magnesium sulphate versus placebo or no treatment was associated with an increase in ‘control of fits’ (one RCT,^30^ 260 infants), seizure control with one anticonvulsant (one RCT,^60^ 52 infants), requiring only one anticonvulsant (two RCTs,^36^, ^51^ 249 infants), improved recovery from abnormal neurological examination within 4 days (one RCT,^60^ 105 infants), a ‘good short‐term neonatal outcome’ (variously defined, four RCTs,^31^, ^40^, ^41^, ^50^ 138 infants), normal neuromotor tone at discharge (Amiel‐Tison criteria) (one RCT,^60^ 67 infants), normal or improved neurological status at discharge (variously defined, three RCTs,^59^, ^60^, ^61^ 213 infants), establishing feeding (oral or tube) at day 7 (two RCTs,^31^, ^46^ 72 infants), and oral feeding at discharge (nine RCTs,^31^, ^40^, ^49^, ^50^, ^51^, ^56^, ^59^, ^60^, ^61^ 815 infants) (Table 1).

Neonatal magnesium sulphate versus placebo or no treatment was associated with shorter duration of seizures (days) (three RCTs,^41^, ^42^, ^60^ 147 infants), recovery from neurological abnormalities (days) (not defined, one RCT,^60^ 120 infants), initiation of ‘feeds’ (days) (not further defined, one RCT,^42^ 62 infants), nasogastric tube feeding, paladai feeding, and direct breastfeeding (all days) (all in one RCT,^60^ 120 infants), hospital stay (days) (one RCT,^42^ 62 infants), and lower weight (kg) and length (cm) (both one RCT,^60^ 41 infants) at 12 months (Table 1).

No clear differences between the magnesium sulphate and placebo or no treatment groups were observed for all remaining secondary outcomes reported (Table 1).

Table S6 presents RCT data not suitable for meta‐analysis (i.e. results for RCTs reported incompletely, such as *p*‐values only, or narrative summaries).

#### Comparison 2: magnesium sulphate and therapeutic hypothermia versus therapeutic hypothermia alone

This comparison included four RCTs,^29^, ^39^, ^47^, ^55^ two conducted exclusively in LMICs^29^, ^47^ (see Table 3 for the Comparison 2 effect estimates, Table 2 for the GRADE Evidence Profile for the primary outcomes, and Appendix S3 for the forest plots).

**TABLE 3:** Results for Comparisons 2–4.

Outcome and subgroup	RCTs, *n*	Infants, *n*	Methods (*I* ^2^)	RR (99% CI)
*Comparison 2: magnesium sulphate and therapeutic hypothermia versus therapeutic hypothermia alone*				
Neonatal death	3	269	F (0%)	0.66 (0.34–1.26)
Neonatal death or DASII score <70 at 12 months	1	115	F (NA)	0.72 (0.40–1.30)
Seizures	3	269	F (0%)	0.94 (0.80–1.10)
Seizures requiring more than one antiseizure medication	1	134	F (NA)	0.79 (0.44–1.42)
Antiseizure therapy at discharge	1	134	F (NA)	0.87 (0.45–1.68)
Abnormal neurological status at discharge (assessed using the Hammersmith Neonatal and Infant Neurological Examination)	1	134	F (NA)	0.93 (0.60–1.42)
Respiratory depression requiring ventilator support	1	134	F (NA)	0.85 (0.59–1.22)
Intracranial haemorrhage				
Grade 1 or 2	2	135	R (78%)	0.68 (0.13–3.50)
Grade 3 or 4	1	60	R (NA)	3.20 (0.14–75.55)
Any	1	60	R (NA)	1.00 (0.94–1.07)
MRI findings				
Cortical lesions	1	36	F (NA)	2.00 (0.14–29.28)
Grey and white matter lesions	1	36	F (NA)	0.38 (0.02–7.43)
Normal	1	36	F (NA)	1.05 (0.83–1.32)
Length of mechanical ventilation (days)	1	75	F (NA)	MD: −0.63 (−2.63 to 1.37)
Length of non‐invasive respiratory support (days)	1	75	F (NA)	MD: 1.22 (−0.13 to 2.57)
Length of oxygen supplementation (days)	1	75	F (NA)	MD: −0.16 (−2.64 to 2.32)
Inhaled nitric oxide treatment	1	75	F (NA)	0.65 (0.11–3.67)
Pulmonary air leak syndrome	1	60	F (NA)	0.21 (0.01–4.26)
Pulmonary haemorrhage	1	60	F (NA)	1.07 (0.07–16.31)
Hypotension				
Mild‐to‐moderate hypotension	1	60	F (NA)	1.07 (0.58–1.99)
Severe hypotension	2	194	F (0%)	0.74 (0.48–1.12)
Any hypotension	1	60	F (NA)	0.95 (0.77–1.18)
PPHN	2	135	F (14%)	0.71 (0.24–2.12)
Catecholamine use				
None	1	75	F (NA)	0.60 (0.33–1.08)
1	1	75	F (NA)	1.38 (0.95–2.01)
2+	1	75	F (NA)	0.97 (0.56–1.69)
Meconium aspiration	1	60	F (NA)	0.64 (0.17–2.45)
Raised LFTs	1	60	F (NA)	1.07 (0.73–1.57)
Renal failure	1	60	F (NA)	0.99 (0.57–1.74)
Major venous thrombosis	1	60	F (NA)	No events
Subcutaneous fat necrosis	1	60	F (NA)	No events
Thrombocytopenia	3	269	F (0%)	1.11 (0.87–1.42)
Prolonged coagulation	1	60	F (NA)	0.99 (0.81–1.21)
Coagulation parameters				
Prothrombin time	1	59	F (NA)	MD: −4.30 (−13.28 to 4.68)
INR time	1	59	F (NA)	MD: 6.90 (−4.02 to 17.82)
aPTT	1	59	F (NA)	MD: −0.20 (−14.78 to 14.38)
Transfusion				
FFP	1	75	F (NA)	0.82 (0.42–1.60)
PLT count	1	75	F (NA)	0.73 (0.18–3.04)
RBC	1	75	F (NA)	0.97 (0.31–3.09)
Necrotizing enterocolitis	1	60	F (NA)	0.21 (0.01–4.26)
Length of antibiotic therapy (days)	1	75	F (NA)	MD: −1.18 (−4.45 to 2.09)
Time to full oral feeding (days)	1	75	F (NA)	MD: −2.00 (−5.50 to 1.50)
Time to full enteral feeding (days)	1	75	F (NA)	MD: −0.30 (−2.80 to 2.20)
Full oral feeding on discharge	1	70	F (NA)	**1.29 (1.06–1.58)**
Length of hospitalization (days)	1	75	F (NA)	MD: −4.22 (−9.43 to 0.99)
DASII score <70 at 12 months	1	96	F (NA)	0.85 (0.29–2.44)
*Comparison 3: magnesium sulphate and melatonin versus melatonin alone*				
Neonatal death	1	90	F (NA)	**0.74 (0.58–0.95)**
Seizures	1	90	F (NA)	1.12 (0.79–1.58)
Intracranial haemorrhage				
Grades 1 and 2	1	90	F (NA)	0.96 (0.89–1.03)
Grades 3 and 4	1	90	F (NA)	5.00 (0.25–101.31)
Any	1	90	F (NA)	1.00 (0.96–1.04)
Hypotension				
Mild to moderate	1	90	F (NA)	1.12 (0.67–1.86)
Severe	1	90	F (NA)	0.86 (0.55–1.36)
Any	1	90	F (NA)	0.97 (0.82–1.15)
Renal failure	1	90	F (NA)	1.00 (0.63–1.59)
Thrombocytopenia	1	90	F (NA)	1.21 (0.92–1.61)
pH				
After 3 days	1	90	F (NA)	**MD: 0.06 (0.04–0.08)**
After 7 days	1	90	F (NA)	**MD: 0.19 (0.18–0.20)**
*Comparison 4: magnesium sulphate versus phenobarbital*				
Neonatal death	1	104	F (NA)	3.00 (0.86–10.46)

*Note*: Statistically significant effect estimates are shown in bold. The test for heterogeneity is represented by the *I* ^2^ statistic; when *I* ^2^ > 30%, summary estimates were calculated using a random effects meta‐analysis.

Abbreviations: aPTT, activated partial thromboplastin clotting time; CI, confidence interval; DASII, Developmental Assessment Scales for Indian Infants; F, fixed effects; FFP, fresh frozen plasma; INR, international normalized ratio; LFT, liver function test; MD, mean difference; MRI, magnetic resonance imaging; NA, not applicable; PPHN, persistent pulmonary hypertension of the newborn; PLT, platelet; R, random effect; RBC, red blood cell; RCT, randomized controlled trial; RR, risk ratio.

##### Primary outcomes

**FIGURE 3:**
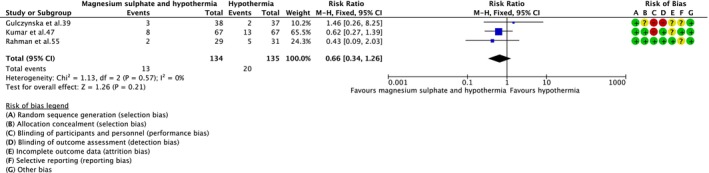
Effect of magnesium sulphate and therapeutic hypothermia versus therapeutic hypothermia alone on neonatal death. Abbreviation: CI, confidence interval.

**FIGURE 4:**
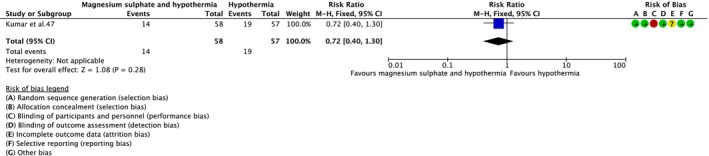
Effect of magnesium sulphate and therapeutic hypothermia versus therapeutic hypothermia alone on death with a Developmental Assessment Scales for Indian Infants score <70 at 12 months. Abbreviation: CI, confidence interval.

No clear difference in neonatal death was observed with magnesium sulphate and therapeutic hypothermia versus therapeutic hypothermia alone (RR = 0.66; 95% CI = 0.34–1.26; three RCTs,^39^, ^47^, ^55^ 269 infants; low‐certainty evidence) (Figure 3). In a sensitivity analysis restricted to the two RCTs^47^, ^55^ at low risk of bias overall, the result was like the overall analysis (RR = 0.56; 95% CI = 0.27–1.16; 194 infants). There was also no clear difference for the composite outcome of neonatal death or Developmental Assessment Scales for Indian Infants score less than 70 at 12 months (RR = 0.72; 95% CI = 0.40–1.30; one RCT,^47^ 115 infants; very‐low‐certainty evidence) (Figure 4); sensitivity analysis not possible and the results were unchanged (one RCT^47^ at low risk of bias overall).

For neonatal death, the subgroup interaction test did not indicate a differential treatment effect based on gestational age (two RCTs with infants born late preterm and at term vs one RCT with infants born at term only) (*χ* ^2^ = 0.06, *p* = 0.80, *I* ^2^ = 0%). A subgroup analysis based on setting (LMIC vs HIC) was not possible; of the three RCTs, one was conducted in a HIC, one across both HICs and LMICs, and one in an LMIC) (see Table S5 for the subgroup effect estimates).

##### Secondary outcomes

Neonatal magnesium sulphate and therapeutic hypothermia versus therapeutic hypothermia alone were associated with an increase in full oral feeding at discharge (one RCT,^39^ 70 infants) (Table 3).

No clear differences between magnesium sulphate and therapeutic hypothermia versus therapeutic hypothermia alone were observed for all remaining secondary outcomes reported (Table 3).

#### Comparison 3: magnesium sulphate and melatonin versus melatonin alone

This comparison included two RCTs conducted in LMICs^33^, ^43^ (see Table 3 for the effect estimates, Table 2 for the GRADE Evidence Profile for primary outcome, and Appendix S3 for the forest plots).

##### Primary outcomes

A reduction in neonatal death was observed with magnesium sulphate and melatonin versus melatonin alone (RR = 0.74; 95% CI = 0.58–0.95; one RCT,^43^ 90 infants; very‐low‐certainty evidence). Death or long‐term major neurodevelopmental disability was not reported. Sensitivity and subgroup analyses were not possible (one included RCT,^43^ not at low risk of bias overall).

##### Secondary outcomes

Neonatal magnesium sulphate and melatonin versus melatonin alone was associated with an increase in neonatal pH after 3 and 7 days (both one RCT,^43^ 90 infants) (Table 3).

No clear differences between magnesium sulphate and melatonin versus melatonin alone were observed for all remaining secondary outcomes (Table 3).

#### Comparison 4: magnesium sulphate versus phenobarbital

This comparison included one RCT conducted in an LMIC^57^ (see Table 3 for the effect estimate, Table 2 for the GRADE Evidence Profile for the primary outcome, and Appendix S3 for the forest plot).

##### Primary outcomes

No clear difference in neonatal death was observed with magnesium sulphate versus phenobarbital (RR = 3.00; 95% CI = 0.86–10.46; one RCT,^57^ 104 infants; very‐low‐certainty evidence). Death or long‐term major neurodevelopmental disability was not reported. Sensitivity and subgroup analyses were not possible (one included RCT,^57^ not at low risk of bias overall).

##### Secondary outcomes

Secondary outcomes were not reported.

### Evidence from non‐randomized studies

Comparison groups differed across the four non‐randomized studies,^48^, ^52^, ^62^, ^63^ which were not suitable for synthesis. Individual results are summarized in the next sections and presented in detail in Table S7.

#### Primary outcomes

Two of the four studies reported on neonatal death. In the first, magnesium sulphate (*n* = 100) versus no magnesium sulphate (*n* = 611) was associated with a reduction in neonatal death, overall (*p* = 0.016) and specifically in infants with HIE (*p* < 0.001) and severe HIE (*p* = 0.027).^52^ In the second, magnesium sulphate (*n* = 50) versus no magnesium sulphate (*n* = 50) was not associated with a clear difference in neonatal death (*p* > 0.05).^62^ Death or long‐term major neurodevelopmental disability was not reported.

#### Secondary outcomes

One study reported an improvement in several short‐term neonatal outcomes (such as measures of seizure control, early initiation of feeding, and neurological abnormalities) with magnesium sulphate (*n* = 50) versus no magnesium sulphate (*n* = 50).^62^ A further study reported an absence of side effects in infants with asphyxiation treated with magnesium sulphate (*n* = 18) versus no magnesium sulphate (*n* = 7).^63^ One study reported on hypotension and respiratory depression with a higher (400 mg/kg, *n* = 7) versus lower (250 mg/kg, *n* = 7) magnesium sulphate dose.^48^

## DISCUSSION

We screened 1008 references; 25 RCTs (2099 infants)^29^, ^30^, ^31^, ^32^, ^33^, ^34^, ^35^, ^36^, ^37^, ^38^, ^39^, ^40^, ^41^, ^42^, ^43^, ^44^, ^45^, ^46^, ^47^, ^49^, ^50^, ^51^, ^53^, ^54^, ^55^, ^56^, ^57^, ^58^, ^59^, ^60^, ^61^ and four non‐randomized studies (871 infants)^48^, ^52^, ^62^, ^63^ were included. Of the 29 studies, 23 (79%) were conducted in LMICs.^29^, ^30^, ^31^, ^32^, ^33^, ^35^, ^40^, ^42^, ^43^, ^44^, ^46^, ^47^, ^49^, ^50^, ^51^, ^52^, ^56^, ^57^, ^58^, ^59^, ^60^, ^61^, ^62^

We observed reductions in neonatal death with magnesium sulphate versus placebo or no treatment (13 RCTs, 1033 infants; low‐certainty evidence), and with magnesium sulphate and melatonin versus melatonin alone (one RCT, 90 infants; very‐low‐certainty evidence). No further reduction in neonatal death was seen with magnesium sulphate and therapeutic hypothermia versus therapeutic hypothermia alone (three RCTs, 269 infants; low‐certainty evidence), nor with magnesium sulphate versus phenobarbital (one RCT, 104 infants; very‐low‐certainty evidence). No reductions in death or long‐term neurodevelopmental disability (one RCT, 41 infants; very‐low‐certainty evidence), but reductions in several short‐term adverse outcomes (certainty of evidence not assessed for secondary outcomes) were observed with magnesium sulphate versus placebo or no treatment.

Importantly, no clear short‐term adverse outcomes or side effects were shown with magnesium sulphate. This is reassuring because unpublished data from the Randomized Asphyxia Trial in the 1990s indicated an increased risk of bradycardia and a trend towards increased mortality with magnesium sulphate.^19^ After suspension of this study, however, it was noted that infants had inadvertently received almost twice the intended dose (the dose commonly used in the RCTs included in this review [250 mg/kg]) because of an error in solution preparation.^19^ Indeed, in the authors' earlier dose comparison study (also included in this review^48^), a higher dose (400 mg/kg) versus a lower dose (250 mg/kg) of magnesium sulphate was associated with increased risks of hypotension and respiratory depression.

The most recent review of magnesium sulphate for infants with perinatal asphyxia and HIE^21^ searched the literature in 2012 and included five RCTs;^31^, ^35^, ^36^, ^41^, ^44^ all were captured in this review. Since then, a further 20 RCTs have been published.^29^, ^30^, ^32^, ^33^, ^39^, ^40^, ^42^, ^43^, ^46^, ^47^, ^49^, ^50^, ^51^, ^55^, ^56^, ^57^, ^58^, ^59^, ^60^, ^61^ As expected, with the widespread implementation of therapeutic hypothermia for HIE as standard care in HICs, all 13 of the newer RCTs^30^, ^32^, ^40^, ^42^, ^46^, ^49^, ^50^, ^51^, ^56^, ^58^, ^59^, ^60^, ^61^ comparing magnesium sulphate with placebo or no treatment were conducted in LMICs (six in Pakistan^30^, ^42^, ^49^, ^56^, ^59^, ^61^ and five in India^32^, ^46^, ^51^, ^58^, ^60^), where evidence for the efficacy of therapeutic hypothermia in neonatal encephalopathy is currently uncertain.^6^, ^7^, ^8^ Similarly, the three RCTs comparing magnesium sulphate with an alternative agent (melatonin^33^, ^43^ or phenobarbital^57^) in the absence of therapeutic hypothermia, were from LMICs. Our search identified a further ongoing RCT (registered this year in Pakistan), designed to compare magnesium sulphate and placebo in infants with HIE (*n* = 178).^64^ This reflects the enduring interest in magnesium sulphate in LMICs where perinatal asphyxia, HIE, and the associated burdens are clearly the highest and the therapies available have been regarded as technically too complex or extremely expensive.^64^

Our review demonstrated a potential reduction in neonatal death with neonatal magnesium sulphate versus placebo or no treatment, which was not observed previously.^21^ However, the certainty of the evidence was judged to be low, including because of significant study limitations (risk of bias). In a sensitivity analysis restricted to studies at low overall risk of bias, this potential reduction was no longer observed; this included only two small RCTs (102 infants). Thus, these data support the need for further research to establish the effects of magnesium sulphate for perinatal asphyxia and HIE in LMICs. If RCTs continue to be conducted, it is crucial that they are high quality in both design and reporting; ideally, they should include long‐term follow‐up. Of note, a core outcome set for neonatal encephalopathy treatment (COHESION) is under development; if applied, it will facilitate the standardization of outcome measurement and reporting in the future.^65^, ^66^

Of the RCTs comparing magnesium sulphate in combination with therapeutic hypothermia and therapeutic hypothermia alone, two were conducted in LMICs^29^, ^47^ and two included HICs (one was a multi‐country study).^39^, ^55^ While the available data did not enable exploration of possible differences in effects according to setting (i.e. in LMICs vs HICs), the results (including a sensitivity analysis restricted to the two RCTs at low risk of bias) do not support a clear benefit of neonatal magnesium sulphate as an adjuvant to therapeutic hypothermia in HIE. This, together with the variable, non‐persuasive evidence of benefit to date from preclinical studies, emphasizes the need for further preclinical evaluation of magnesium sulphate for neonatal neuroprotection (alone and in combination with therapeutic hypothermia), to ensure safety and identify optimal treatment regiments (including dose, route, and timing of administration), before conducting further RCTs.^17^, ^18^, ^19^

A recent systematic review and meta‐analysis demonstrated the breadth of neuroprotective adjuvants to therapeutic hypothermia evaluated in RCTs to date, including γ‐aminobutyric acid receptor agonists (phenobarbital, topiramate), n‐methyl‐d‐aspartic acid receptor antagonists (magnesium sulphate, xenon), neurogenic and angiogenic agents (darbepoetin alpha, erythropoietin), umbilical cord blood cells, glucocorticoids (hydrocortisone), and antioxidants (melatonin).^67^ As recognized by others,^68^, ^69^ magnesium sulphate is not the sole, or indeed favoured agent, in the current quest for an adjuvant to augment therapeutic hypothermia‐mediated neuroprotection. There is increasing evidence to suggest that neuroprotective adjuvants may be best administered after (vs during) therapeutic hypothermia. For example, a delayed rise in circulating cytokines (and associated white matter and basal ganglia injury) has been observed after therapeutic hypothermia.^70^ An increased odds of electrographic seizures during rewarming (vs the 12 hours before) has also been demonstrated in infants receiving therapeutic hypothermia, which was further associated with death or disability at 2 years.^71^ To the best of our knowledge, whether there is a role for delaying magnesium sulphate administration, to follow therapeutic hypothermia, has not yet been determined.

### Limitations

The main limitations of this review relate to the clinical heterogeneity across studies (designs, settings, participating infants, and particularly the criteria for asphyxia and presence of HIE [including severity], magnesium sulphate regimens, and outcomes reported) and the variable quality of studies (risk of bias). Only four of the 25 RCTs included in this review were considered as low risk of bias overall.^31^, ^42^, ^47^, ^55^ While we attempted to explore expected variation in treatment effects through subgroup and sensitivity analyses, our ability to do so was limited because of the paucity of the outcome data or data stratified according to participant or treatment characteristics not being available. Statistical heterogeneity was detected for a small number of secondary outcomes (not observed for the primary outcomes) and probably attributed to the previously described variations across RCTs. For our primary outcomes (death, composite of death, or long‐term major neurodevelopmental disability), 18 (72%)^31^, ^35^, ^36^, ^39^, ^41^, ^42^, ^43^, ^44^, ^46^, ^47^, ^50^, ^51^, ^55^, ^56^, ^57^, ^58^, ^60^, ^61^ and two (8%)^47^, ^60^ of the 25 RCTs reported usable data. Most secondary outcomes were reported sparsely (and were poorly defined), many by only one or two RCTs. Sample sizes were commonly small, further limiting the ability to detect small, clinically important differences in outcomes, such as long‐term neurodevelopment, which was reported in only five (20%)^35^, ^36^, ^42^, ^47^, ^60^ RCTs at varying time points (6–24 months).

### CONCLUSIONS

The certainty of the evidence for the use of magnesium sulphate in perinatal asphyxia and HIE is low to very low. As such, further robust research surrounding neonatal magnesium sulphate for neuroprotection is justified. This should include a pipeline of high‐quality preclinical studies informing future clinical trials to determine its stand‐alone effects in LMICs and its effects in combination with or after therapeutic hypothermia in HICs.

## CONFLICT OF INTEREST STATEMENT

The authors have no conflicts of interest relevant to this article to disclose.

## Supporting information


**Figure S1:** PRISMA flow diagram

**Figure S2:** Risk of bias across randomized trials

**Figure S3:** Risk of bias for individual randomized trials

**Appendix S1:** Search strategy

**Appendix S2:** Reports excluded at the full‐text review

**Appendix S3:** Forest plots, Comparisons 1–4

**Table S1:** Characteristics of the randomized trials

**Table S2:** Characteristics of the non‐randomized studies

**Table S3:** Risk of bias of randomized trials

**Table S4:** Risk of bias of non‐randomized studies

**Table S5:** Subgroup analyses for neonatal death, Comparisons 1 and 2

**Table S6:** Additional data from randomized trials not able to be included in meta‐analyses

**Table S7:** Results from non‐randomized studies

## Data Availability

The data that supports the findings of this study are available in the supplementary material of this article.
