# Supplementary figures and images for: Neonatal magnesium sulphate for neuroprotection: A systematic review and meta‐analysis
Source: Dev Med Child Neurol. 2024 Mar 11;66(9):1157–72. doi: 10.1111/dmcn.15899 (PMC11579813; doi:10.1111/dmcn.15899)

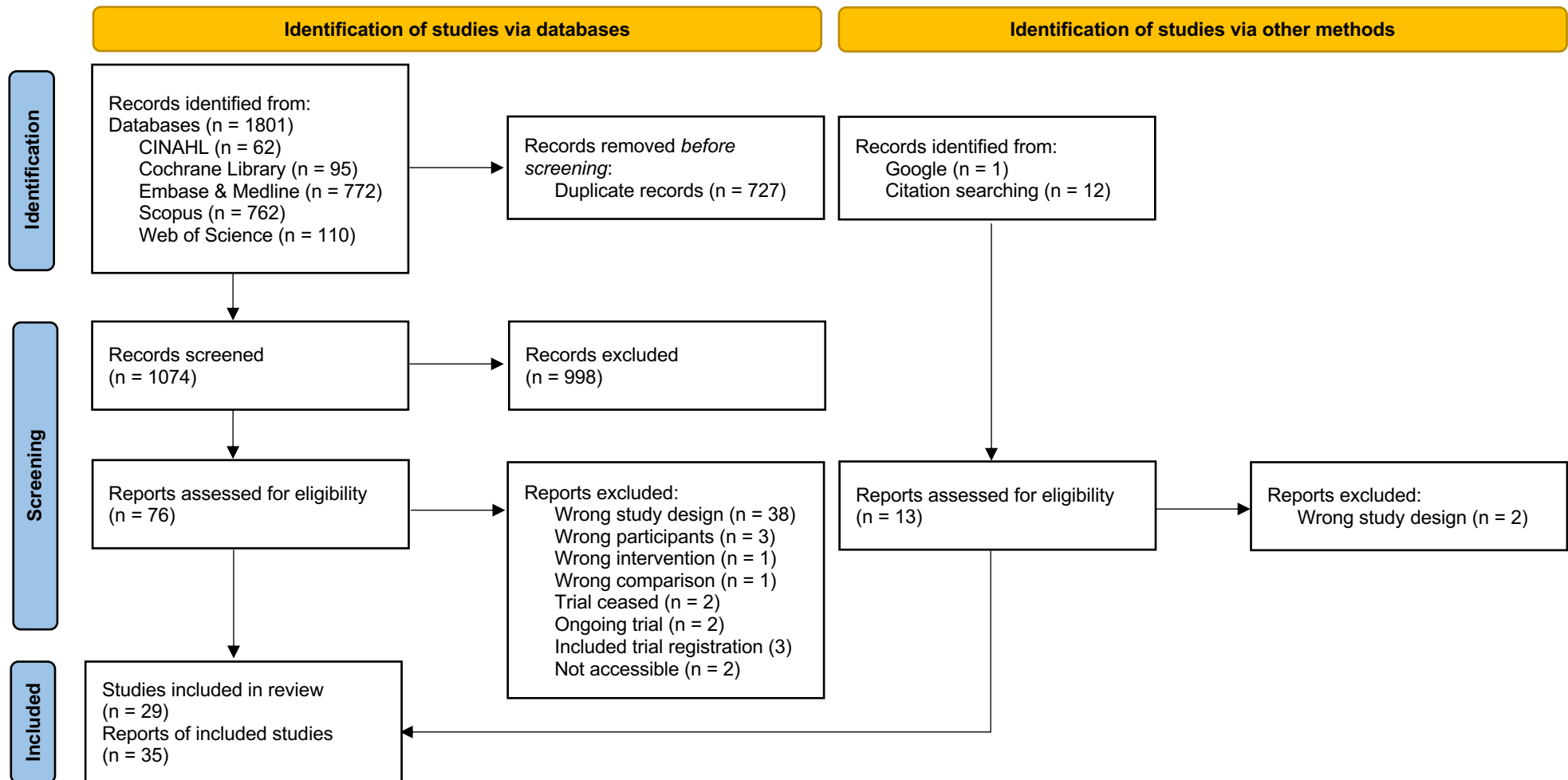

Supplement: Supplementary file 1 — Figure S1: PRISMA flow diagram [file DMCN-66-1157-s009.pdf]

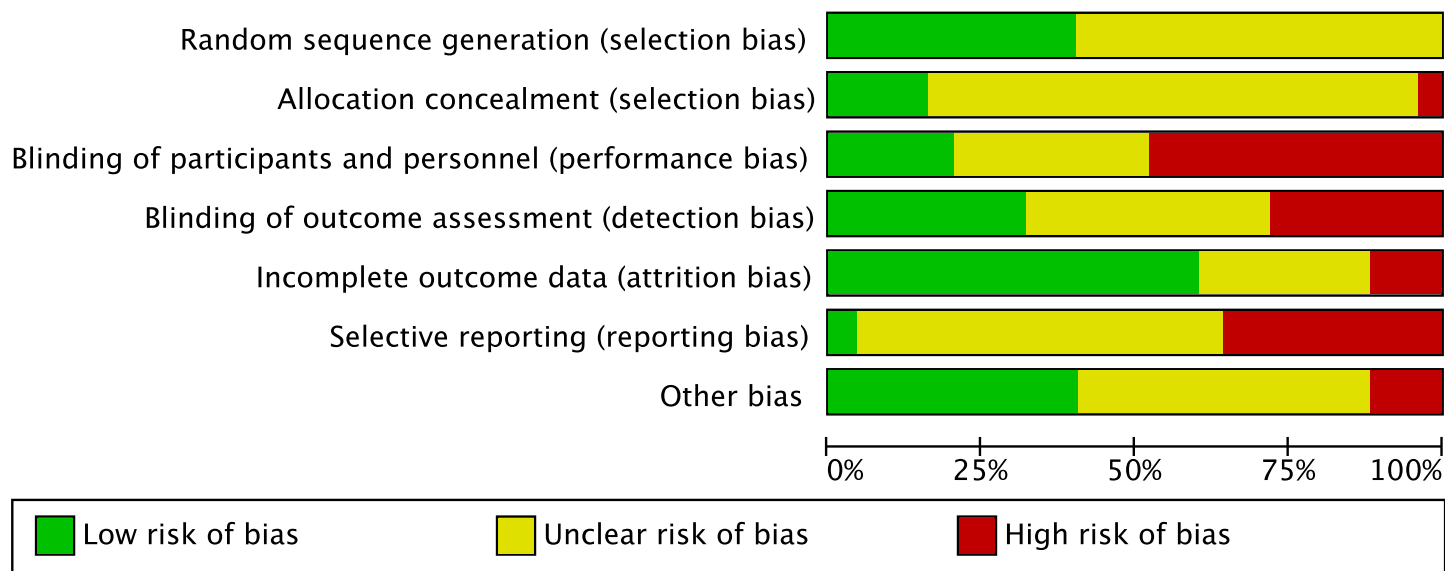

Supplement: Supplementary file 2 — Figure S2: Risk of bias across randomized trials [file DMCN-66-1157-s010.pdf]
