# Supplementary material for: Neonatal magnesium sulphate for neuroprotection: A systematic review and meta‐analysis
Source: Dev Med Child Neurol. 2024 Mar 11;66(9):1157–72. doi: 10.1111/dmcn.15899 (PMC11579813; doi:10.1111/dmcn.15899)
Supplement: Supplementary file 3 — Figure S3: Risk of bias for individual randomized trials [file DMCN-66-1157-s005.pdf]

	Random sequence generation (selection bias)	Allocation concealment (selection bias)	Blinding of participants and personnel (performance bias)	Blinding of outcome assessment (detection bias)	Incomplete outcome data (attrition bias)	Selective reporting (reporting bias)	Other bias
Abdel-Aziz 2021	?	?	−	−	+	−	−
Ahmad 2019	?	?	−	−	+	−	?
Bhat 2009	+	?	+	+	+	?	+
El Farargy 2020	?	?	?	?	?	?	?
Gathwala 2010	+	?	−	+	+	?	+
Groenendaal 2002	?	?	+	+	+	−	+
Gulczynska 2018	+	?	−	−	?	?	+
Hossain 2013	?	?	?	?	?	?	?
Ichiba 2002	?	?	−	−	+	?	+
Iqbal 2021	+	+	+	?	−	?	+
Khan 2022	?	?	−	−	+	?	+
Khashaba 2006	?	+	+	+	+	?	+
Kumar 2015	?	?	−	?	+	−	?
Kumar 2022	+	+	−	+	?	+	+
Mehmood 2015	?	?	?	?	+	−	−
Mullalli-Bime 2016	?	?	?	?	?	?	?
Nanda 2002	+	?	−	+	−	?	?
Rahman 2015	+	+	+	+	+	?	+
Rashid 2015	?	?	?	?	+	−	?
Riaz 2021	?	?	−	−	+	−	−
Riyaz Ahmed 2016	+	?	−	−	+	−	?
Sajid 2018	+	?	?	?	+	−	?
Savitha 2016	+	−	?	+	−	?	?
Siddiqui 2021	?	?	−	?	?	?	?
Singh 2015	?	?	?	?	?	?	?
