# Supplementary material for: Neonatal magnesium sulphate for neuroprotection: A systematic review and meta‐analysis
Source: Dev Med Child Neurol. 2024 Mar 11;66(9):1157–72. doi: 10.1111/dmcn.15899 (PMC11579813; doi:10.1111/dmcn.15899)
Supplement: Supplementary file 4 — Appendix S1: Search strategy [file DMCN-66-1157-s001.docx]

**Appendix S1** Search strategy

CINAHL search strategy and results

| **No.** | **Searches** | **Results** |
| --- | --- | --- |
| S1 | (MH “Magnesium sulfate”) | 1,748 |
| S2 | “magnesium sulfate” or “magnesium sulphate” or mgso4 | 2,405 |
| S3 | S1 OR S2 | 2,405 |
| S4 | (MH “Infant+”) | 291,050 |
| S5 | (MH “Intensive Care, Neonatal+”) | 6,581 |
| S6 | (baby or babies or neonat* or infan* or newborn* or “new born”) | 555,122 |
| S7 | S4 OR S5 OR S6 | 555,242 |
| S8 | (MH “Asphyxia Neonatorum”) | 1,325 |
| S9 | (MH “Hypoxia, Brain+”) | 2,538 |
| S10 | (MH “Cerebral Ischemia+”) | 21,179 |
| S11 | (MH “Anoxia+”) | 7,895 |
| S12 | (MH “Asphyxia”) | 1,593 |
| S13 | (hypoxi* or hypoxe* or hypoxae* or ischemi* or ischaemi* or anoxi* or HIE or encephalopat* or asphyxia* or depress*) | 349,624 |
| S14 | S8 OR S9 OR S10 OR S11 OR S12 OR S13 | 350,345 |
| S15 | S3 AND S7 AND S14 | 62 |

Cochrane Library search strategy and results

| **No.** | **Searches** | **Results** |
| --- | --- | --- |
| #1 | MeSH descriptor: [Magnesium Sulfate] explode all trees | 1185 |
| #2 | ((magnesium next sulfate) or (magnesium next sulphate) or MgSO4):ti,ab,kw | 3316 |
| #3 | #1 or #2 | 3316 |
| #4 | MeSH descriptor: [Infant] explode all trees | 41440 |
| #5 | MeSH descriptor: [Intensive Care, Neonatal] explode all trees | 375 |
| #6 | (baby or babies or neonat* or infan* or newborn* or (new next born)):ti,ab,kw | 90539 |
| #7 | #4 or #5 or #6 | 90539 |
| #8 | MeSH descriptor: [Asphyxia Neonatorum] explode all trees | 260 |
| #9 | MeSH descriptor: [Brain Ischemia] explode all trees | 5875 |
| #10 | MeSH descriptor: [Hypoxia] explode all trees | 3113 |
| #11 | MeSH descriptor: [Asphyxia] explode all trees | 112 |
| #12 | (hypoxi* or hypoxe* or hypoxae* or ischemi* or ischaemi* or anoxi* or HIE or encephalopat* or asphyxia* or depress*):ti,ab,kw | 168388 |
| #13 | #8 or #9 or #10 or #11 or #12 | 169209 |
| #14 | #3 and #7 and #13 | 95 |

Embase and Medline search strategy and results

| **No.** | **Searches** | **Results** |
| --- | --- | --- |
| 1 | exp Magnesium Sulfate/ | 24698 |
| 2 | ((magnesium adj sulfate) or (magnesium adj sulphate) or MgSO4).mp. | 31673 |
| 3 | 1 or 2 | 31673 |
| 4 | exp Infant/ | 2371862 |
| 5 | Intensive Care, Neonatal/ | 33566 |
| 6 | (baby or babies or neonat$ or infan$ or newborn$ or (new adj born)).mp. | 3255720 |
| 7 | 4 or 5 or 6 | 3255720 |
| 8 | exp Asphyxia Neonatorum/ | 13765 |
| 9 | exp Brain Ischemia/ | 340243 |
| 10 | exp Hypoxia/ | 239162 |
| 11 | exp Asphyxia/ | 23427 |
| 12 | (hypoxi$ or hypoxe$ or hypoxae$ or ischemi$ or ischaemi$ or anoxi$ or HIE or encephalopat$ or asphyxi$ or depress$).mp. | 3582849 |
| 13 | 8 or 9 or 10 or 11 or 12 | 3612809 |
| 14 | 3 and 7 and 13 | 997 |
| 15 | exp Animals/ | 56710258 |
| 16 | exp Humans/ | 46447588 |
| 17 | 15 not 16 | 10262670 |
| 18 | 14 not 17 | 904 |
| 19 | remove duplicates from 18 | 772 |

Scopus search strategy and results

| **No.** | **Searches** | **Results** |
| --- | --- | --- |
| 1 | ( TITLE-ABS-KEY ( “magnesium sulphate” OR “magnesium sulphate” OR mgso4 ) ) | 28,517 |
| 2 | ( TITLE-ABS-KEY ( baby OR babies OR neonat* OR infan* OR newborn* OR “new born” ) ) | 1,992,524 |
| 3 | ( TITLE-ABS-KEY ( hypoxi* OR hypoxe* OR hypoxae* OR ischemi* OR ischaemi* OR anoxi* OR hie OR encephalopat* OR asphyxia* OR depress* ) ) | 2,160,281 |
| 4 | 1 and 2 and 3 | 762 |

Web of Science search strategy and results

| **No.** | **Searches** | **Results** |
| --- | --- | --- |
| 1 | TS=((“magnesium sulphate” OR “magnesium sulphate” OR MgSO4) ) | 9,413 |
| 2 | TS=((baby or babies or neonat* or infan* or newborn* or “new born”) ) | 950,032 |
| 3 | TS=((hypoxi* or hypoxe* or hypoxae* or ischemi* or ischaemi* or anoxi* or HIE or encephalopat* or asphyxia* or depress*)) | 1,639,946 |
| 4 | #3 AND #2 AND #1 | 110 |
