# Supplementary material for: Neonatal magnesium sulphate for neuroprotection: A systematic review and meta‐analysis
Source: Dev Med Child Neurol. 2024 Mar 11;66(9):1157–72. doi: 10.1111/dmcn.15899 (PMC11579813; doi:10.1111/dmcn.15899)
Supplement: Supplementary file 5 — Appendix S2: Reports excluded at the full‐text review [file DMCN-66-1157-s002.docx]

**Appendix S2** Reports excluded at full text review

| **No.** | **Citation** | **Reason for exclusion** |
| --- | --- | --- |
| 1 | Al-Alaiyan S. Multicenter randomized controlled trial of therapeutic hypothermia plus magnesium sulfate versus therapeutic hypothermia plus placebo in the management of term and near term infants with hypoxic ischemic encephalopathy (the mag cool study): A pilot study (Journal of Clinical Neonatology (158-163)). J Clin Neonatol 2015; 4(4): 293. | Wrong study design: erratum |
| 2 | Anabrees J. Magnesium sulphate for newborns with HIE; Synopsis of evidence from a systematic review. J Clin Neonatol 2013; 2(3): 114-6. | Wrong study design: systematic review synopsis |
| 3 | Bell SG. Hypoxic-ischemic encephalopathy and serum magnesium monitoring and maintenance. Neonatal Netw 2016; 35(3): 159-63. | Wrong study design: narrative review |
| 4 | Bhat MA, Charoo BA, Bhat JI, Ahmad SM, Ali SW, Mufti MUH. Magnesium sulfate in severe perinatal asphyxia: a randomized, placebo-controlled trial. Obstet Gynecol Surv 2009; 64(9): 573-7. | Wrong study design: editorial comment re: included RCT (Bhat 2009) |
| 5 | Cowan A, Pearce J. Magnesium sulphate for neuroprotective therapy during resuscitation - a literature review. Australas J Paramedicine 2014; 11(1): 19-20. | Wrong study design: narrative review |
| 6 | Dunne JT, Miliigan JE, Thomas BW. The effect of magnesium sulfate on anoxia and resuscitation in the neonate. Am J Obstet Gynecol 1971; 109(3): 369-74. | Wrong participants: animal study |
| 7 | Evans D. Neuroprotection following intrapartum asphyxia: the Randomised Asphyxia Study (RAST). J Neonatal Nurs 1996; 2(3): 14-8. | Trial ceased (protocol) |
| 8 | Fai GZ, Gao SL, Yan ZH. Efficacy of magnesium sulfate in the treatment of neonatal hypoxic ischemic encephalopathy. Medical Journal of Qilu 2000; 15(2): 90-2. | Not accessible: record unable to be accessed (unable to determine eligibility based on Chinese abstract) |
| 9 | Gathwala G. Neuronal protection with magnesium. Indian J Pediatr 2001; 68: 417-9. | Wrong study design: review (‘annotation’) |
| 10 | Gonzalez FF. Neuroprotection strategies for term encephalopathy. Semin Pediatr Neurol 2019; 32: 100773. | Wrong study design: narrative review |
| 11 | Greenwood A, Evans J, Smit E. New brain protection strategies for infants with hypoxic-ischaemic encephalopathy. Pediatr Child Health 2018; 28(9): 405-11. | Wrong study design: narrative review |
| 12 | Gulczynska E, Cyranowicz B, Kesiak M, Lerch E, Nowiczewski M, Talar T. Therapeutic hypothermia for hypoxic-ischaemic encephalopathy in the neonates-first polish experience. J Matern Fetal Neonatal Med 2010; 23 (Suppl 1): 41. | Wrong study design: no control group |
| 13 | Hagag AA, El Frargy MS, Abd El-Latif AE. Vitamin D as an adjuvant therapy in neonatal hypoxia: is it beneficial? Endocr Metab Immune Disord Drug Targets 2019; 19(3): 341-8. | Wrong comparison: same MgSO4 regimen in intervention and control groups |
| 14 | Ichiba H, Yokoi T, Tamai H, Ueda T, Kim T, Yamano T. Neurodevelopmental outcome of infants with birth asphyxia treated with magnesium sulfate. Pediatr Int 2006; 48(1): 70-5. | Wrong study design: no control group |
| 15 | Jelin AC, Salmeen K, Gano D, Burd I, Thiet MP. Perinal neuroprotection update. F1000Res 2016; 5(F1000 Faculty Rev): 1939. | Wrong study design: narrative review |
| 16 | Juul SE, Ferriero DM. Pharmacologic Neuroprotective Strategies in Neonatal Brain Injury. Clin Perinatol 2014; 41(1): 119-31. | Wrong study design: narrative review |
| 17 | Kent A, Kecskes Z, Cochrane T. Magnesium sulfate for term infants following perinatal asphyxia (Protocol). Cochrane Database Syst Rev 2021; 2: CD004494. | Wrong study design: systematic review protocol |
| 18 | Khashaba MT, Shouman BO, Shaltour AA, Al-Marsafawy HW, Abdel-Aziz MM, Aly H. Proinflammatory cytokines, neurological sequelea and magnesium sulfate (MgSO4) in neonatal asphyxia. Pediatric Academic Societies Annual Meeting May 2-5 2009, Baltimore MD, United States. | Not accessible: conference abstract unable to be accessed |
| 19 | Khashaba M. Neuroprotection in HI – Egyptian data. J Matern Fetal Neonatal Med 2010; 23 (Suppl 1): 41. | Wrong study design: no control group (conference abstract) |
| 20 | Kornacka MK. Magnesium sulphate in the treatment of ischemic-hypoxic neonatal encephalopathy. Neurol Neurochir Pol 2001; 35(2): 299-308. | Wrong study design: narrative review |
| 21 | Leonard AS, Mikati MA. What is their fate after magnesium sulfate? Neonatol 2010; 98(2): 206-7. | Wrong study design: letter to the editor |
| 22 | Levene MI, Evans DJ, Mason S, Brown J. An international network for evaluating neuroprotective therapy after severe birth asphyxia. Semin Perinatol 1999; 23(3): 226-233. | Wrong study design: commentary (describes international network, pilot study initiation) |
| 23 | Levene MI. Cool treatment for birth asphyxia, but what's next? Arch Dis Child Fetal Neonatal Ed 2010; 95(3): F154-7. | Wrong study design: narrative review (‘leading article’) |
| 24 | Lingam I, Robertson NJ. Magnesium as a neuroprotective agent: a review of its use in the fetus, term infant with neonatal encephalopathy, and the adult stroke patient. Dev Neurosci 2018; 40(1): 1-12. | Wrong study design: narrative review |
| 25 | Maroszynska I, Sobolewska B, Gulczynska E, Zylinska L, Lerch E, Kicinska M et al. Can magnesium sulfate reduce the risk of cerebral injury after perinatal asphyxia? Acta Pol Pharm 1999; 56(6): 469-73. | Wrong study design: no control group |
| 26 | McAdams RM, Berube MW. Emerging therapies and management for neonatal encephalopathy-controversies and current approaches. J Perinatol 2021; 41(4): 661-74. | Wrong study design: narrative review |
| 27 | McGuire W. Perinatal asphyxia. Clin Evid 2007; 11: 320. | Wrong study design: systematic review |
| 28 | Nair J, Jumar VHS. Current and emerging therapies in the management of hypoxic ischemic encephalopathy in neonates. Children 2018; 5(7): 19. | Wrong study design: narrative review |
| 29 | Nonomura M, Harada S, Asada Y, Matsumura H, Iwami H, Tanaka Y et al. Combination therapy with erythropoietin, magnesium sulfate and hypothermia for hypoxic-ischemic encephalopathy: an open-label pilot study to assess the safety and feasibility. BMC Pediatr 2019; 19: 13. | Wrong study design: no control group |
| 30 | Oorschot DE. Cerebral palsy and experimental hypoxia-induced perinatal brain injury: is magnesium protective? Magnes Res 2000; 13(4): 265-73. | Wrong study design: narrative review |
| 31 | Pazaiti A, Spandou E, Karkavelas G, Georgiou T, Karalis P, Melios G et al. Sensorimotor function and neuropathology for the evaluation of long lasting consequences following hypoxic-ischemic(HI) neonatal brain injury and after MgSO4 treatment. Pediatr Res 2004; 55(4): 75A. | Wrong participants: animal study |
| 32 | Pius S, Bello M, Ambe JP, Yenti M, Genesis R, Clement AY, et al. Magnesium sulphate treated severly asphyxiated neonates, their characteristic and outcome. AJPR 2018; 1(2): 1-9. | Wrong study design: no control group |
| 33 | Poon WB, Ho SKY. Pilot case series on the use of magnesium sulphate as neuroprotection in hie babies in the cooling era. Arch Dis Child 2012; 97 (Suppl 2): A319. | Wrong study design: no control group |
| 34 | Rajeshwari K. Magnesium sulfate improves outcome in severe perinatal asphyxia: commentary. Indian Pediatr 2009; 46(6): 550. | Wrong study design: short commentary (‘clipping’) |
| 35 | Ramsay S. Getting the chemistry right in trials. Lancet 1998; 351(9115): 1528. | Trial ceased (short commentary) |
| 36 | Romero AM, Nannig PM. Hypomagnesemia in newborns with hypoxic ischemic encephalopathy and whole-body hypothermia. Rev Chil Pediatr 2020; 91(1): 116-21. | Wrong intervention: not MgSO4 for for perinatal asphyxia and HIE (given for hypomagnesemia) |
| 37 | Shi J, Ziong Y, Mu DZ. Clinical evidence for the pharmacological therapy in newborn infants with hypoxic-ischemic encephalopathy. Zhongguo Dang Dai Er Ke Za Zhi 2009; 11(9): 740-4. | Wrong study design: systematic review |
| 38 | Solevag AL, Schmolzer GM, Cheung PY. Novel interventions to reduce oxidative-stress related brain injury in neonatal asphyxia. Free Radic Biol Med 2019; 142: 113-22. | Wrong study design: narrative review |
| 39 | Tagin M, Shah PS, Lee K-S. Magnesium for newborns with hypoxic-ischemic encephalopathy: a systematic review and meta-analysis. J Perinatol 2013; 33: 663-9. | Wrong study design: systematic review |
| 40 | Viera MFB. Future of neuroprotection: Therapeutic strategies in the clinical frontier. J Perinat Med 2017; 45 (Suppl 2): 75. | Wrong study design: narrative review |
| 41 | Westermaier T, Stetter C, Kunze E, Willner N, Raslan F, Vince GH et al. Magnesium treatment for neuroprotection in ishemic diseases of the brain. Exp Transl Stroke Med 2013; 5: 6. | Wrong study design: narrative review |
| 42 | Whitelaw A. Systematic review of therapy after hypoxic-ischaemic brain injury in the perinatal period. Semin Neonatol 2000; 5: 33-40. | Wrong study design: systematic review |
| 43 | Whitelaw A, Thoresen M. Clinical trials of treatments after perinatal asphyxia. Current Opin Pediatr 2002; 14(6): 664-8. | Wrong study design: narrative review |
| 44 | Wu Q, Chen W, Sinha B, Tu Y, Manning S, Thomas N, et al. Neuroprotective agents for neonatal hypoxic-ischemic brain injury. Drug Discov Today 2015; 20(11): 1372-81. | Wrong study design: narrative review |
| 45 | Yildiz EP, Ekici B, Tatli B. Neonatal hypoxic ischemic encephalopathy: an update on disease pathogenesis and treatment. Expert Rev Neurother 2017; 17(5): 449-59. | Wrong study design: review |
| 46 | Zen Lee CY, Chakranon P, Huey Lee SW. Comparative efficacy and safety of neuroprotective therapies for neonates with hypoxic ischemic encephalopathy: A network meta-analysis. Front Pharmacol 2019; 10: 1211. | Wrong study design: systematic review |
| 47 | Zhou KQ, Davidson JO, Bennet L, Gunn AJ. Combination treatments with therapeutic hypothermia for hypoxic-ischemic neuroprotection. Dev Med Child Neurol 2020; 62(10): 1131-7. | Wrong study design: narrative review |
| 48 | A multicenter randomized controlled trial of therapeutic hypothermia plus magnesium sulphate (mgso4) versus therapeutic hypothermia plus placebo in the management of term and near term babies with hypoxic ischemic encephalopathy. https://clinicaltrials.gov/study/NCT01646619 | Trial registration (included RCT: Rahman 2015) |
| 49 | Erythropoietin, magnesium sulfate and hypothermia for hypoxic-ischemic encephalopathy. https://trialsearch.who.int/Trial2.aspx?TrialID=JPRN-UMIN000032627 | Wrong study design (trial registration for Nonomura 2019, no control group) |
| 50 | Erythropoietin and magnesium sulphate in hypoxic ischaemic encelpalopathy. https://trialsearch.who.int/Trial2.aspx?TrialID=PACTR201812814507775 | Ongoing trial (recruitment status: pending) |
| 51 | Magnesium sulphate in perinatal asphyxia (Magsulf). https://clinicaltrials.gov/show/NCT00553072 | Trial registration (included RCT: Bhat 2009) |
| 52 | Magnesium sulfate as an adjunct to cooling of babies who do not cry at birth. https://trialsearch.who.int/Trial2.aspx?TrialID=CTRI/2018/06/014594 | Trial registration (included RCT: Kumar 2022) |
| 53 | Prophylactic effect of magnesium sulfate on intraventricular hemorrhage of very low birth weight neonates. https://trialsearch.who.int/Trial2.aspx?TrialID=IRCT201307181162N21 | Wrong participants: not neonates with evidence of perinatal asphyxia and HIE born at 35 weeks’ gestation or later |
| 54 | Magnesium suplphate in protection against brain injury in newborn. https://trialsearch.who.int/Trial2.aspx?TrialID=CTRI/2018/07/015057 | Ongoing trial (recruitment status: not yet recruiting) |
