# Supplementary material for: Neonatal magnesium sulphate for neuroprotection: A systematic review and meta‐analysis
Source: Dev Med Child Neurol. 2024 Mar 11;66(9):1157–72. doi: 10.1111/dmcn.15899 (PMC11579813; doi:10.1111/dmcn.15899)
Supplement: Supplementary file 6 — Appendix S3: Forest plots, Comparisons 1–4 [file DMCN-66-1157-s006.docx]

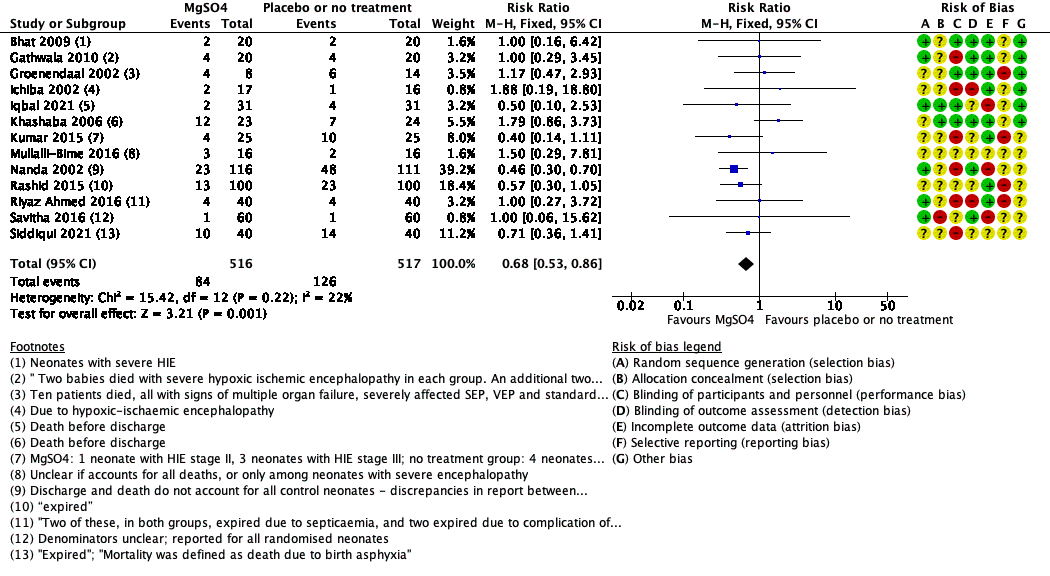


**Figure 1.1**: Forest plot of comparison: 1 MgSO_4_ versus placebo or no treatment, outcome: 1.1 Neonatal death.


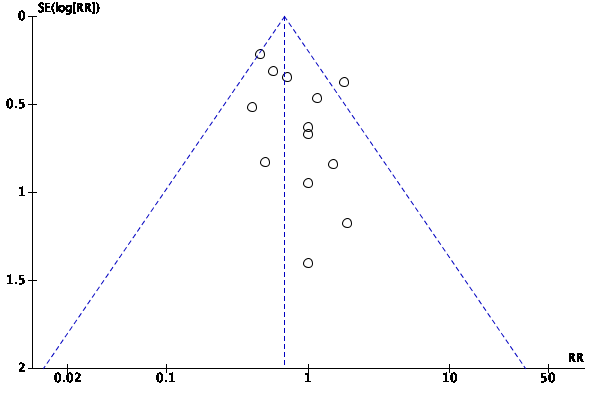


**Figure 1.1.2**: Funnel plot of comparison: 1 MgSO_4_ versus placebo or no treatment, outcome: 1.1 Neonatal death.


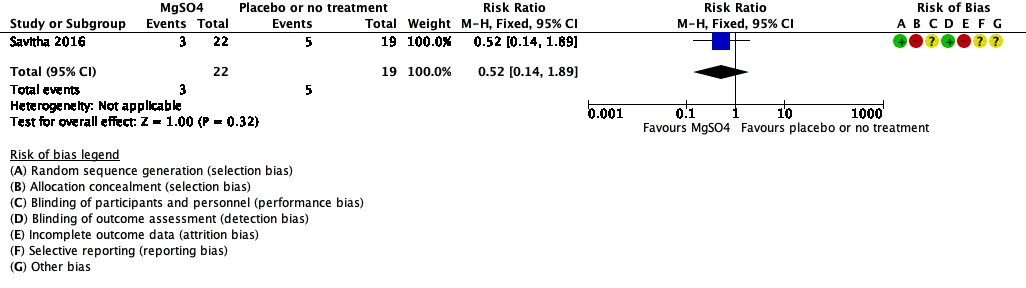


**Figure 1.2**: Forest plot of comparison: 1 MgSO_4_ versus placebo or no treatment, outcome: 1.2 Death or neurodevelopmental disability at 12 months.


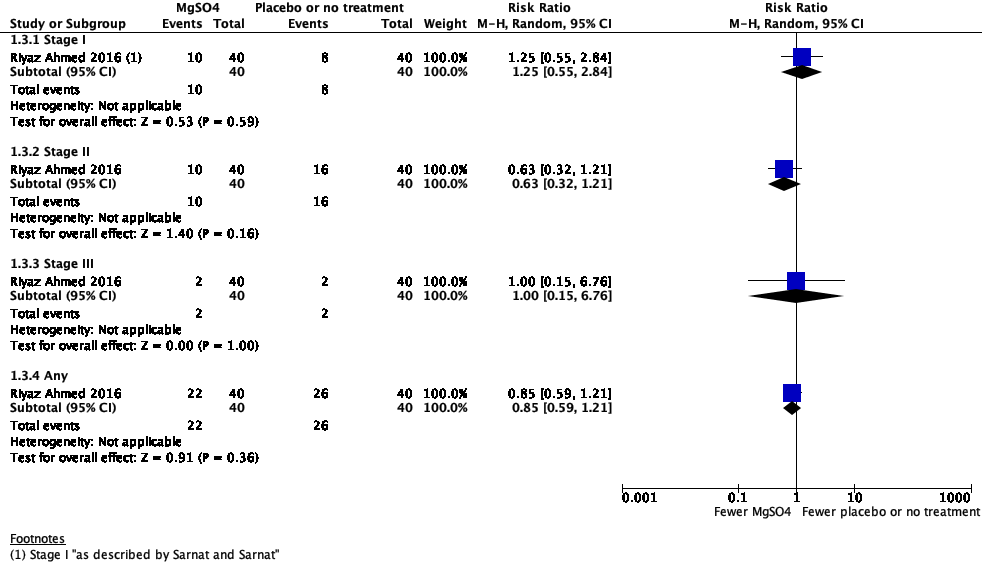


**Figure 1.3**: Forest plot of comparison: 1 MgSO_4_ versus placebo or no treatment, outcome: 1.3 Hypoxic-ischaemic encephalopathy.


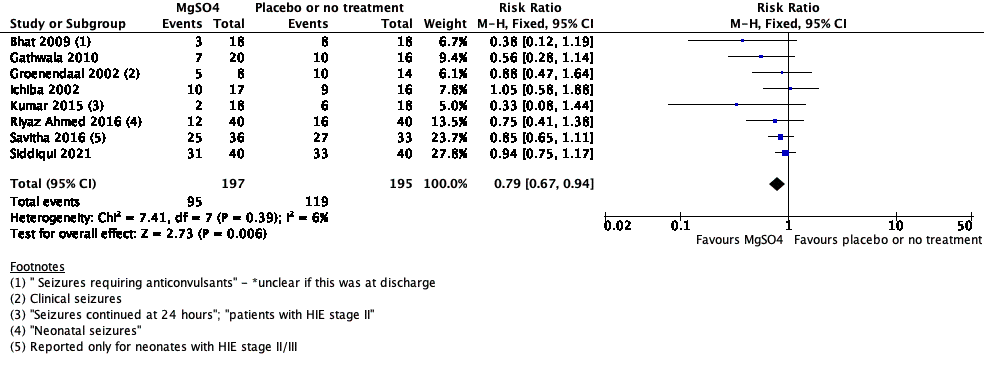


**Figure 1.4**: Forest plot of comparison: 1 MgSO_4_ versus placebo or no treatment, outcome: 1.4 Seizures.


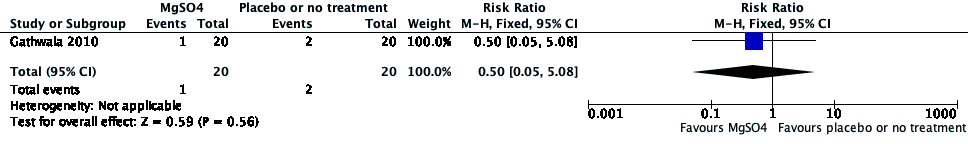


**Figure 1.5**: Forest plot of comparison: 1 MgSO_4_ versus placebo or no treatment, outcome: 1.5 Refractory seizures.


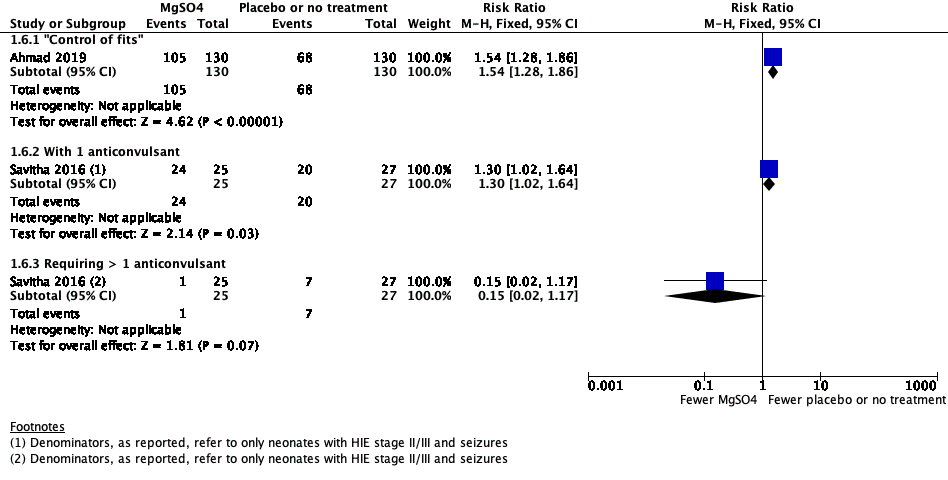


**Figure 1.6**: Forest plot of comparison: 1 MgSO_4_ versus placebo or no treatment, outcome: 1.6 Seizure control.


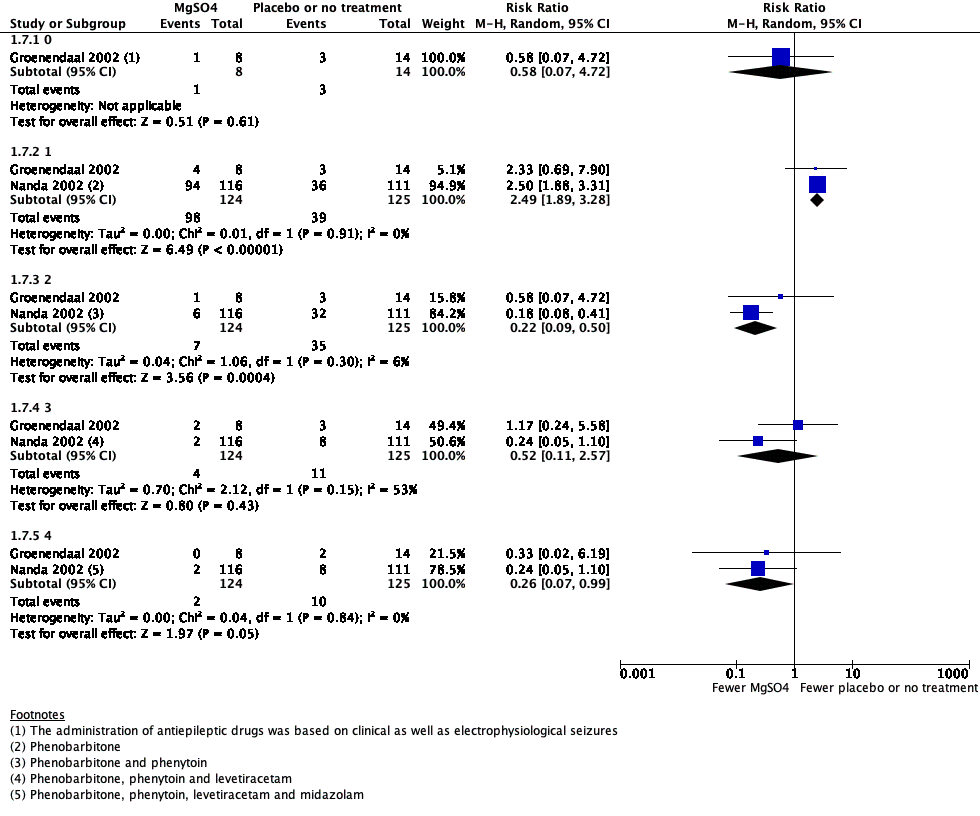


**Figure 1.7**: Forest plot of comparison: 1 MgSO_4_ versus placebo or no treatment, outcome: 1.7 Use of anticonvulsant drugs.


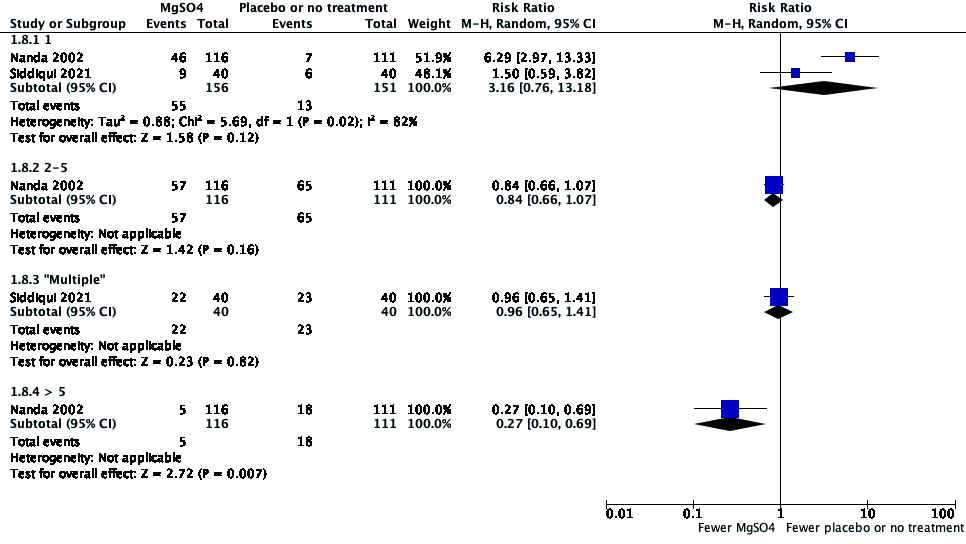


**Figure 1.8**: Forest plot of comparison: 1 MgSO_4_ versus placebo or no treatment, outcome: 1.8 Number of seizures.


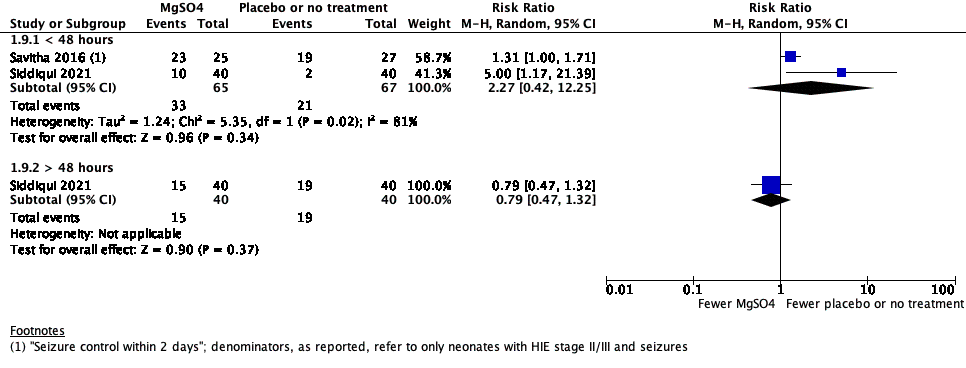


**Figure 1.9**: Forest plot of comparison: 1 MgSO_4_ versus placebo or no treatment, outcome: 1.9 Duration of seizures.


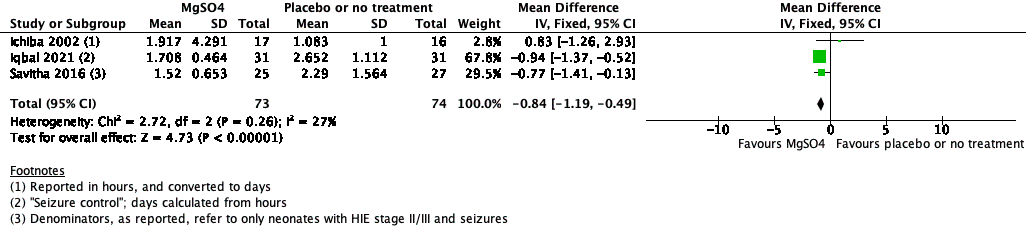


**Figure 1.10**: Forest plot of comparison: 1 MgSO_4_ versus placebo or no treatment, outcome: 1.10 Duration of seizures (days).


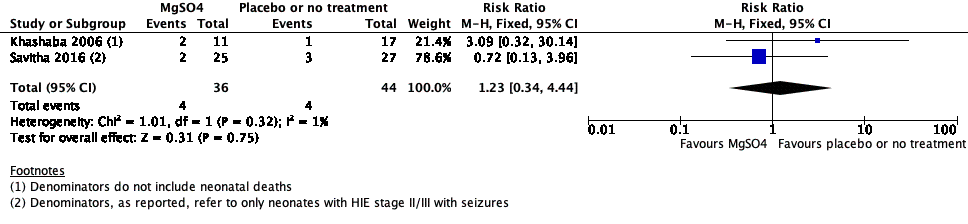


**Figure 1.11**: Forest plot of comparison: 1 MgSO_4_ versus placebo or no treatment, outcome: 1.11 Anticonvulsant required at discharge.


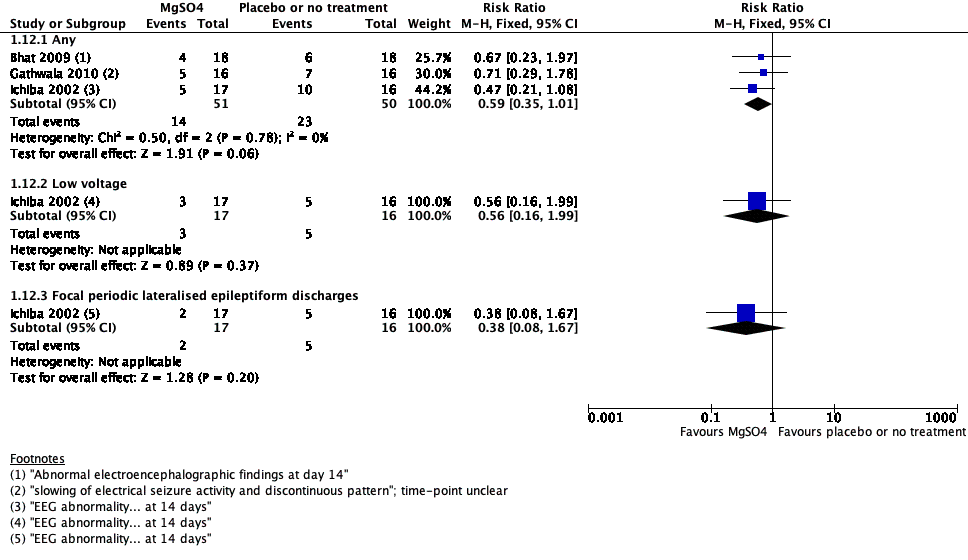


**Figure 1.12**: Forest plot of comparison: 1 MgSO_4_ versus placebo or no treatment, outcome: 1.12 EEG abnormalities.


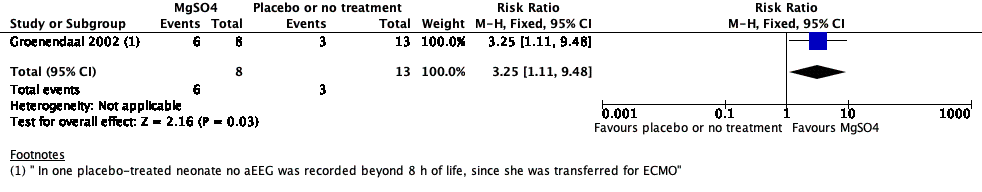


**Figure 1.13**: Forest plot of comparison: 1 MgSO_4_ versus placebo or no treatment, outcome: 1.13 Suppression of aEEG background pattern before administration (3 hours) to 12 hours.


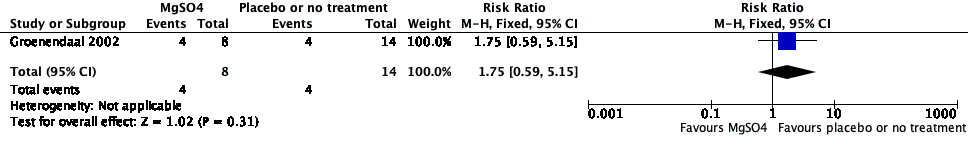


**Figure 1.14**: Forest plot of comparison: 1 MgSO_4_ versus placebo or no treatment, outcome: 1.14 CUS subcortical echodensities within 4 hours of birth.


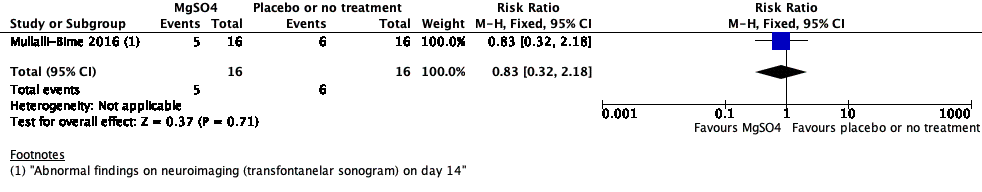


**Figure 1.15**: Forest plot of comparison: 1 MgSO_4_ versus placebo or no treatment, outcome: 1.15 CUS abnormalities at day 14.


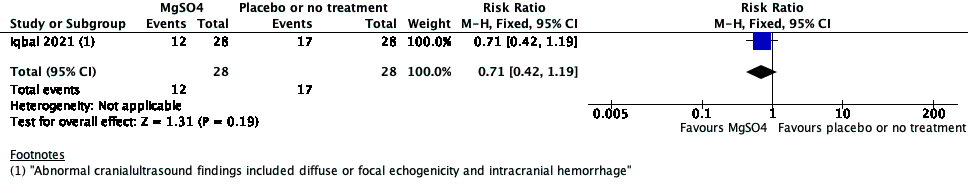


**Figure 1.16**: Forest plot of comparison: 1 MgSO_4_ versus placebo or no treatment, outcome: 1.16 CUS abnormalities at discharge.


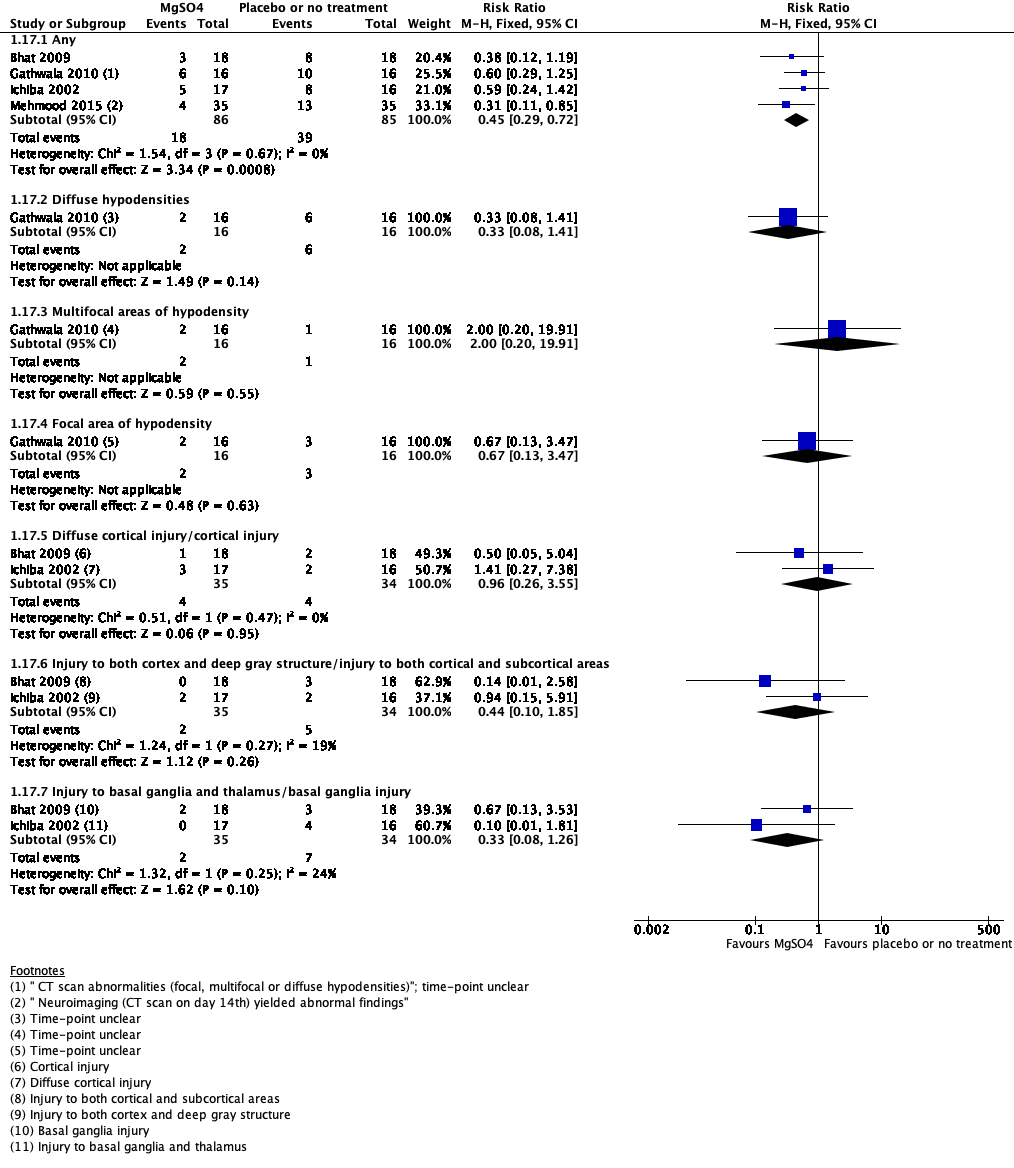


**Figure 1.17**: Forest plot of comparison: 1 MgSO_4_ versus placebo or no treatment, outcome: 1.17 CT abnormalities at day 14.


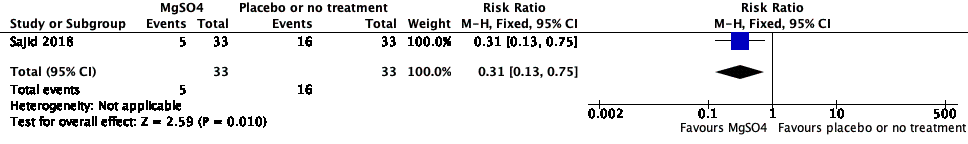


**Figure 1.18**: Forest plot of comparison: 1 MgSO_4_ versus placebo or no treatment, outcome: 1.18 CT abnormalities at discharge.


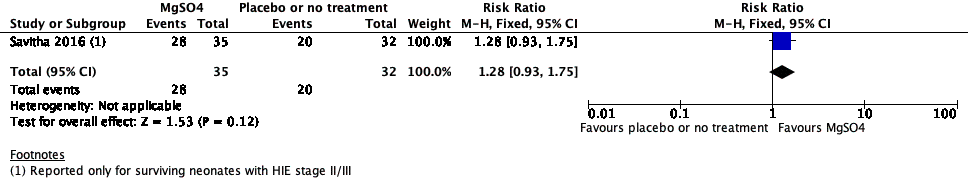


**Figure 1.19**: Forest plot of comparison: 1 MgSO_4_ versus placebo or no treatment, outcome: 1.19 “Normal neuroimaging” at discharge.


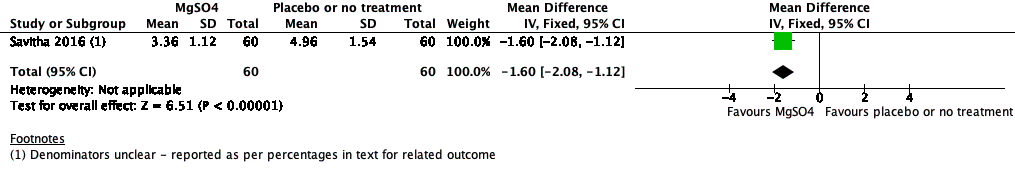


**Figure 1.20**: Forest plot of comparison: 1 MgSO_4_ versus placebo or no treatment, outcome: 1.20 Duration of recovery from neurological abnormalities (days).


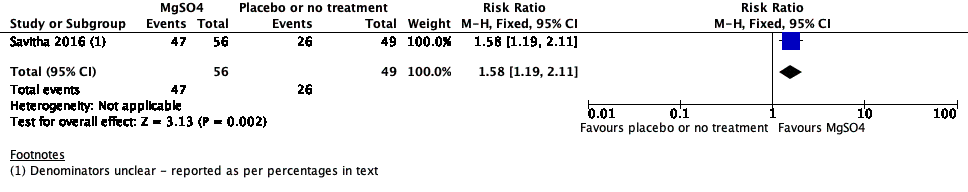


**Figure 1.21**: Forest plot of comparison: 1 MgSO_4_ versus placebo or no treatment, outcome: 1.21 Recovery from abnormal neurological examination within 4 days.


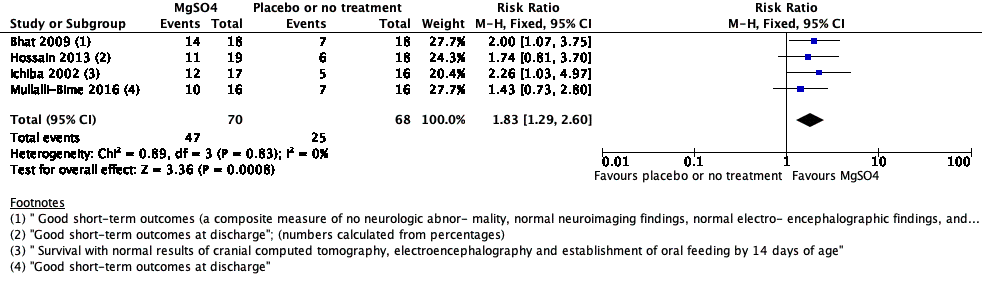


**Figure 1.22**: Forest plot of comparison: 1 MgSO_4_ versus placebo or no treatment, outcome: 1.22 Good short-term neonatal outcome.


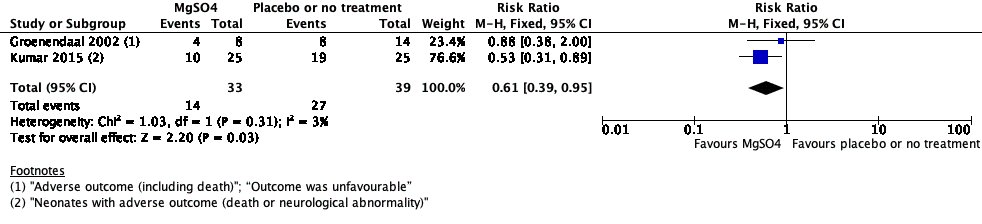


**Figure 1.23**: Forest plot of comparison: 1 MgSO_4_ versus placebo or no treatment, outcome: 1.23 Composite neonatal adverse outcome (including death).


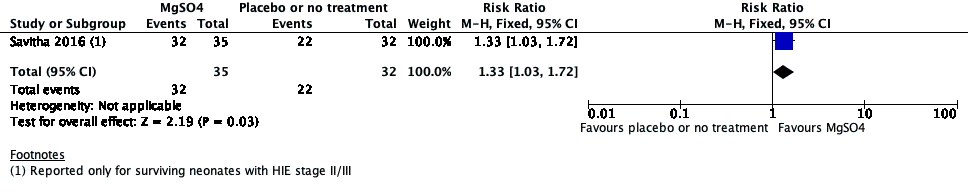


**Figure 1.24**: Forest plot of comparison: 1 MgSO_4_ versus placebo or no treatment, outcome: 1.24 Normal neuromotor tone (Amiel-Tison criteria) at discharge.


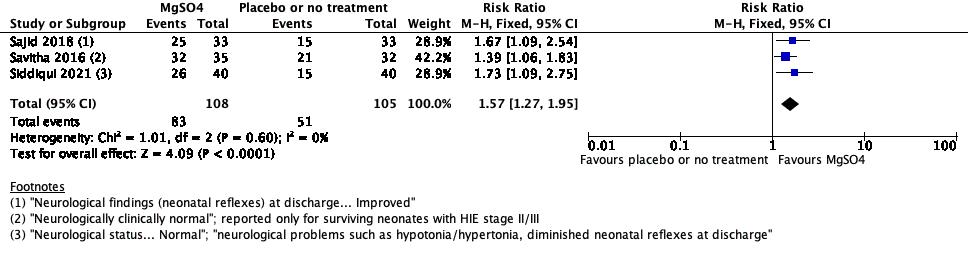


**Figure 1.25**: Forest plot of comparison: 1 MgSO_4_ versus placebo or no treatment, outcome: 1.25 Neurologic status normal/improved at discharge.


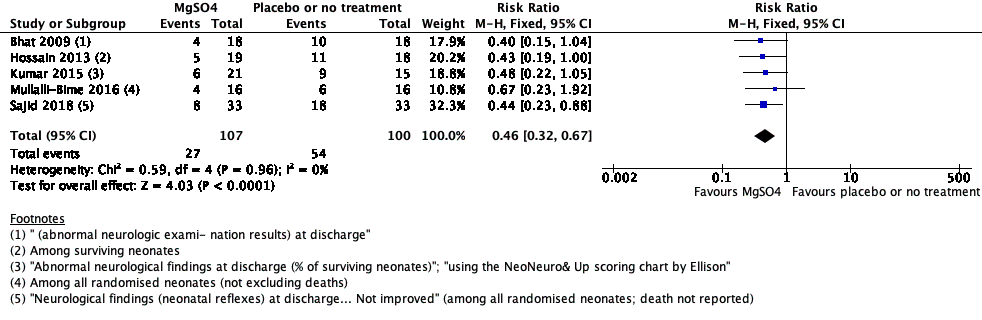


**Figure 1.26**: Forest plot of comparison: 1 MgSO_4_ versus placebo or no treatment, outcome: 1.26 Neurologic abnormalities at discharge.


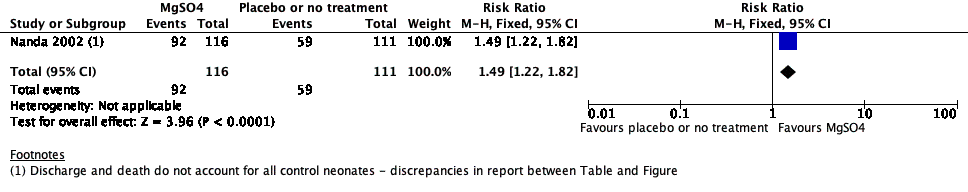


**Figure 1.27**: Forest plot of comparison: 1 MgSO_4_ versus placebo or no treatment, outcome: 1.27 “Final outcome... Discharge”.


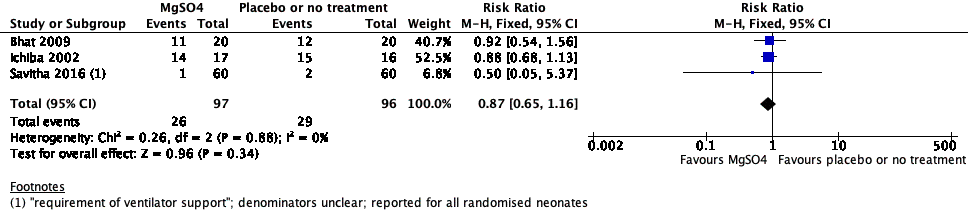


**Figure 1.28**: Forest plot of comparison: 1 MgSO_4_ versus placebo or no treatment, outcome: 1.28 Assisted ventilation.


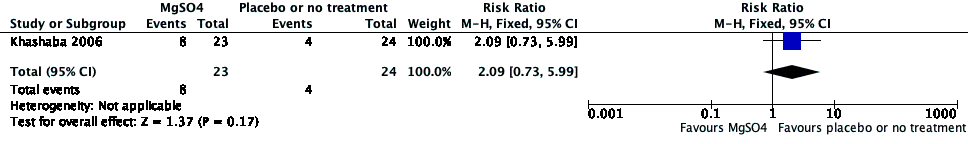


**Figure 1.29**: Forest plot of comparison: 1 MgSO_4_ versus placebo or no treatment, outcome: 1.29 Mechanical ventilation at 72 hours.


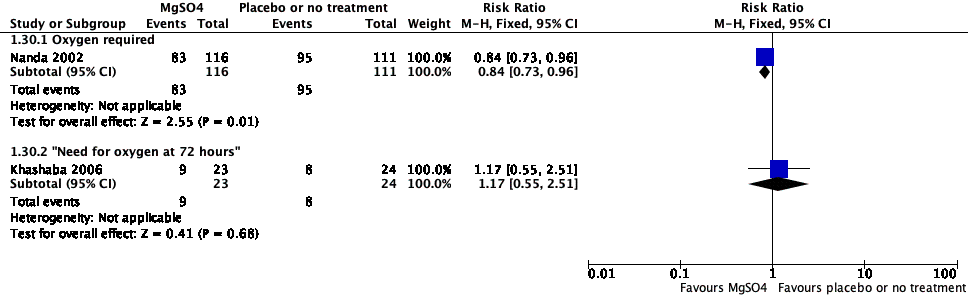


**Figure 1.30**: Forest plot of comparison: 1 MgSO_4_ versus placebo or no treatment, outcome: 1.30 Need for oxygen.


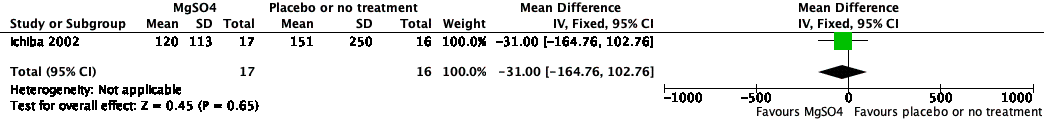


**Figure 1.31**: Forest plot of comparison: 1 MgSO_4_ versus placebo or no treatment, outcome: 1.31 Duration of assisted ventilation (hours).


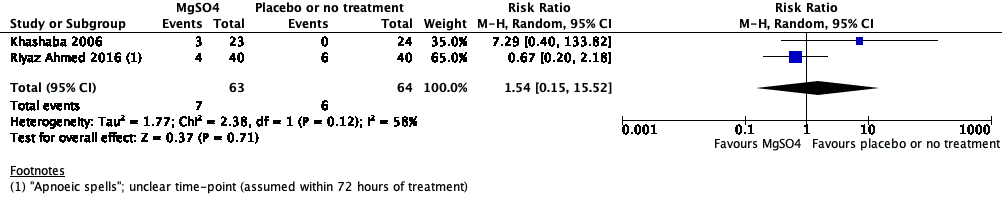


**Figure 1.32**: Forest plot of comparison: 1 MgSO_4_ versus placebo or no treatment, outcome: 1.32 Apnoea at 72 hours.


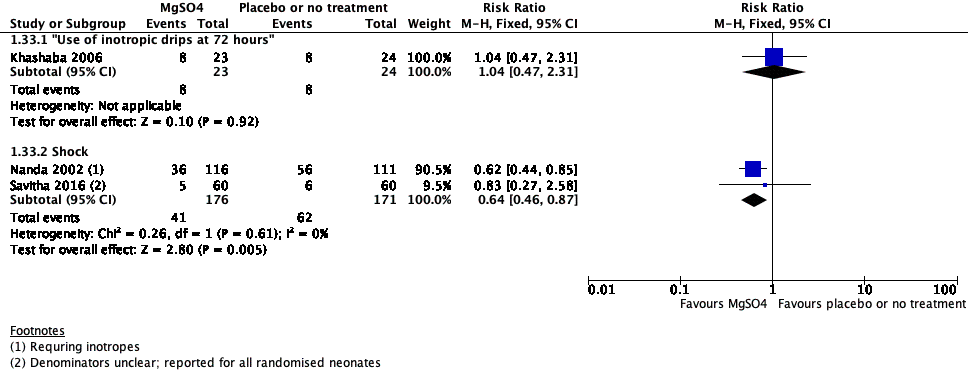


**Figure 1.33**: Forest plot of comparison: 1 MgSO_4_ versus placebo or no treatment, outcome: 1.33 Shock/use of inotropes.


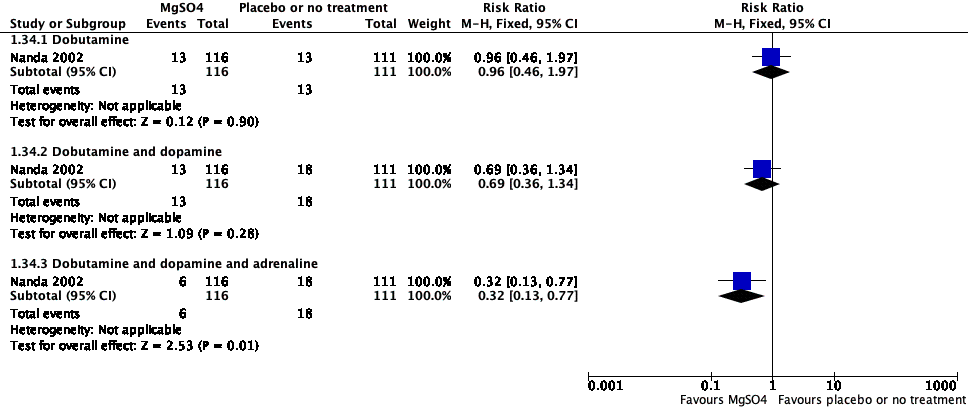


**Figure 1.34**: Forest plot of comparison: 1 MgSO_4_ versus placebo or no treatment, outcome: 1.34 Number of inotropes required.


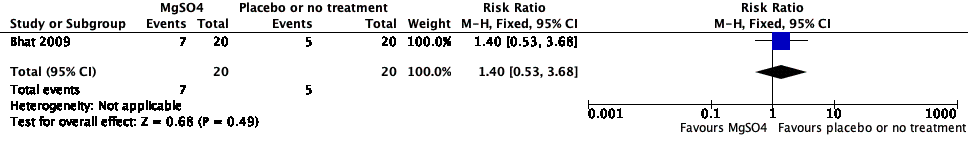


**Figure 1.35**: Forest plot of comparison: 1 MgSO_4_ versus placebo or no treatment, outcome: 1.35 Hypotension requiring pressor support.


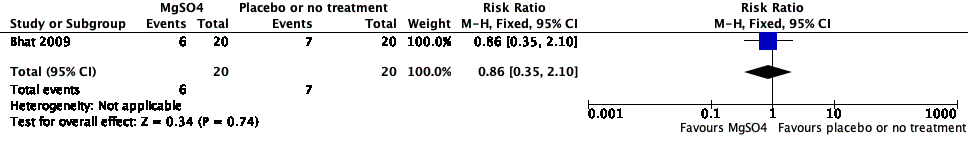


**Figure 1.36**: Forest plot of comparison: 1 MgSO_4_ versus placebo or no treatment, outcome: 1.36 Persistent pulmonary hypertension.


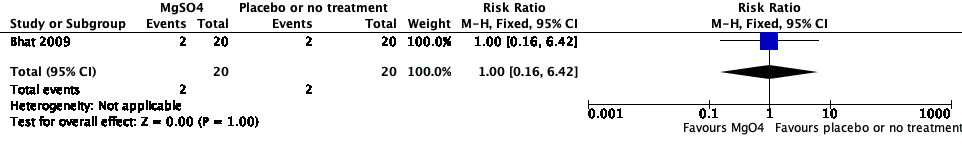


**Figure 1.37**: Forest plot of comparison: 1 MgSO_4_ versus placebo or no treatment, outcome: 1.37 Disseminated intravascular coagulation.


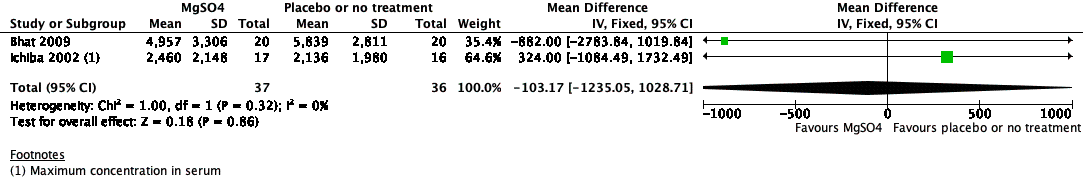


**Figure 1.38**: Forest plot of comparison: 1 MgSO_4_ versus placebo or no treatment, outcome: 1.38 Creatine (phospho)kinase (U/L).


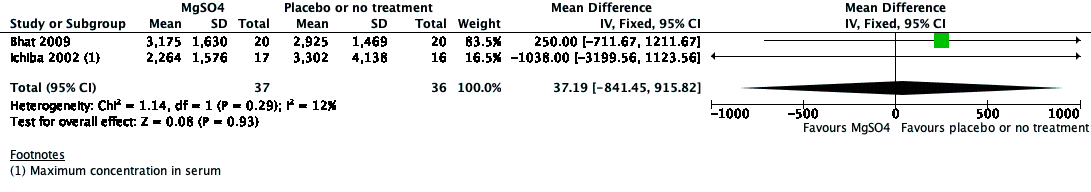


**Figure 1.39**: Forest plot of comparison: 1 MgSO_4_ versus placebo or no treatment, outcome: 1.39 Lactate dehydrogenase (U/L).


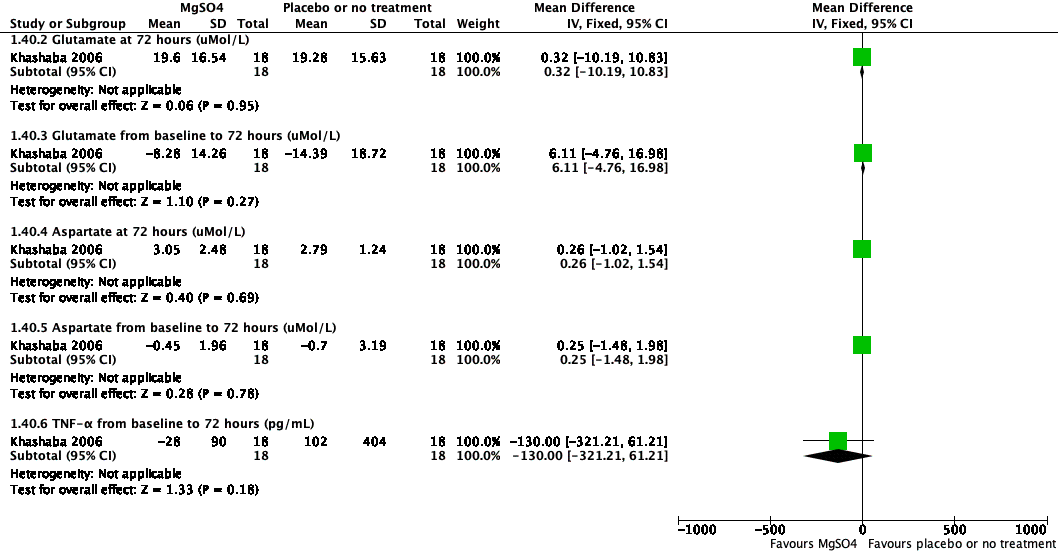


**Figure 1.40**: Forest plot of comparison: 1 MgSO_4_ versus placebo or no treatment, outcome: 1.40 CSF concentrations.


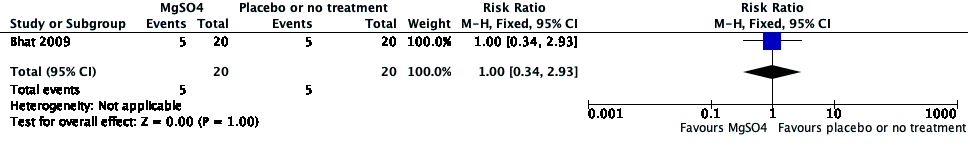


**Figure 1.41**: Forest plot of comparison: 1 MgSO_4_ versus placebo or no treatment, outcome: 1.41 Oliguria.


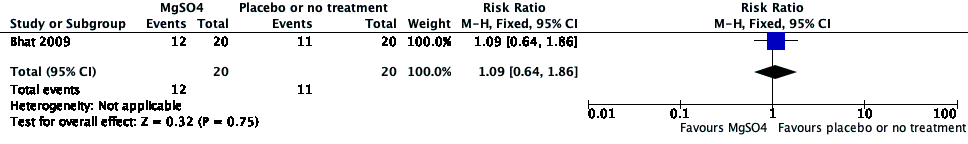


**Figure 1.42**: Forest plot of comparison: 1 MgSO_4_ versus placebo or no treatment, outcome: 1.42 Renal failure.


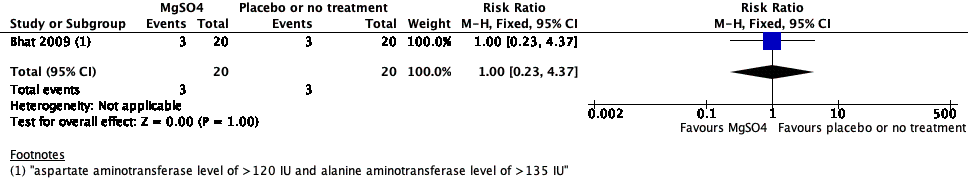


**Figure 1.43**: Forest plot of comparison: 1 MgSO_4_ versus placebo or no treatment, outcome: 1.43 Hepatic dysfunction.


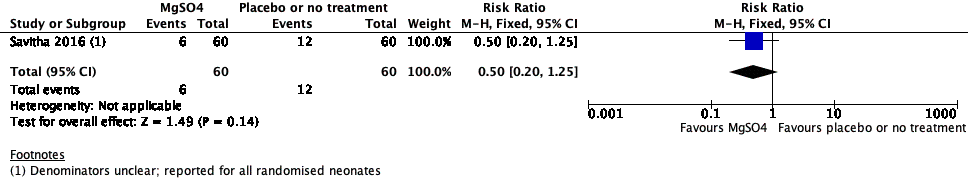


**Figure 1.44**: Forest plot of comparison: 1 MgSO_4_ versus placebo or no treatment, outcome: 1.44 Acute kidney injury.


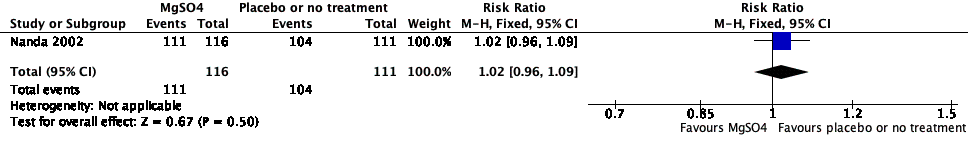


**Figure 1.45**: Forest plot of comparison: 1 MgSO_4_ versus placebo or no treatment, outcome: 1.45 Antibiotics required.


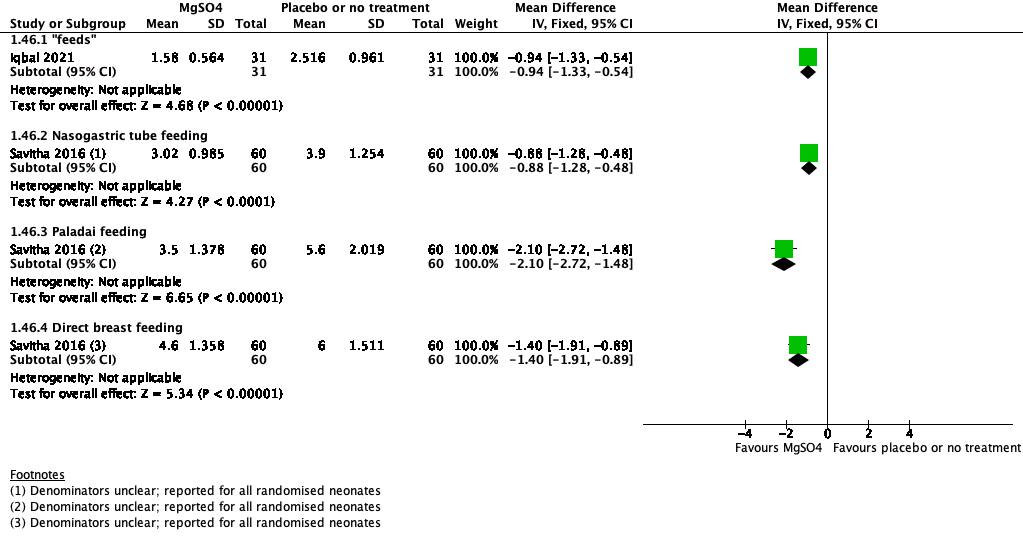


**Figure 1.46**: Forest plot of comparison: 1 MgSO_4_ versus placebo or no treatment, outcome: 1.46 Duration for initiation of feeding (days).


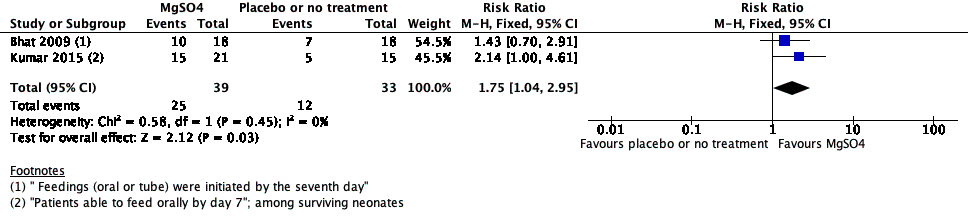


**Figure 1.47**: Forest plot of comparison: 1 MgSO_4_ versus placebo or no treatment, outcome: 1.47 Established feedings at day 7.


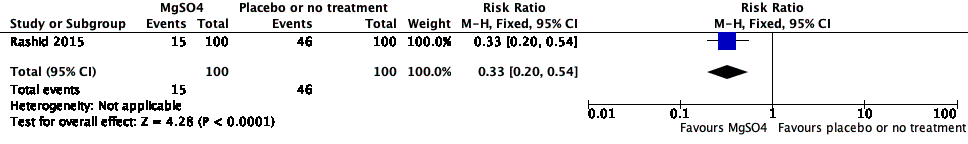


**Figure 1.48**: Forest plot of comparison: 1 MgSO_4_ versus placebo or no treatment, outcome: 1.48 “NG feed on day 14 of life”.


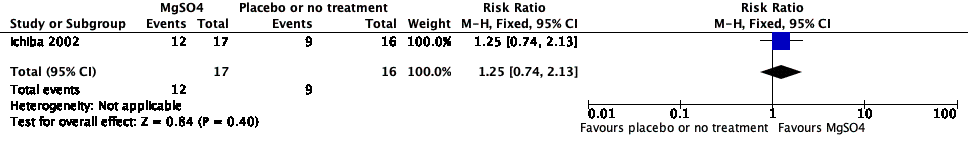


**Figure 1.49**: Forest plot of comparison: 1 MgSO_4_ versus placebo or no treatment, outcome: 1.49 Established oral feedings at day 14.


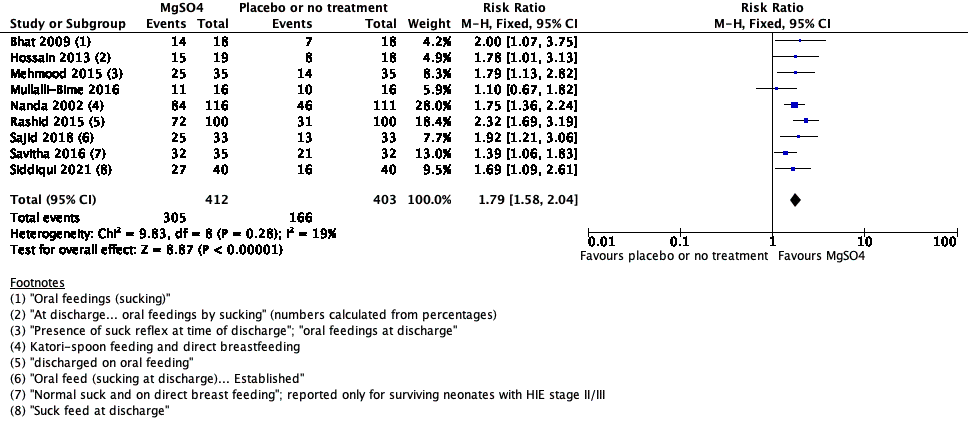


**Figure 1.50**: Forest plot of comparison: 1 MgSO_4_ versus placebo or no treatment, outcome: 1.50 Oral feedings at discharge.


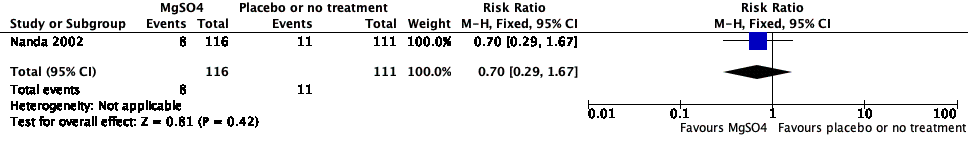


**Figure 1.51**: Forest plot of comparison: 1 MgSO_4_ versus placebo or no treatment, outcome: 1.51 OGT feeding at discharge.


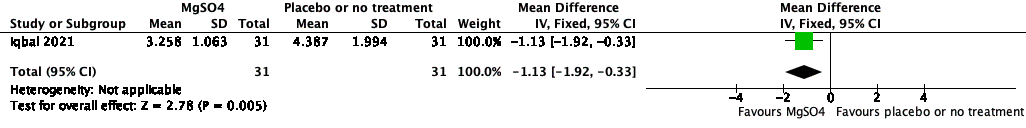


**Figure 1.52**: Forest plot of comparison: 1 MgSO_4_ versus placebo or no treatment, outcome: 1.52 Duration of stay (days).


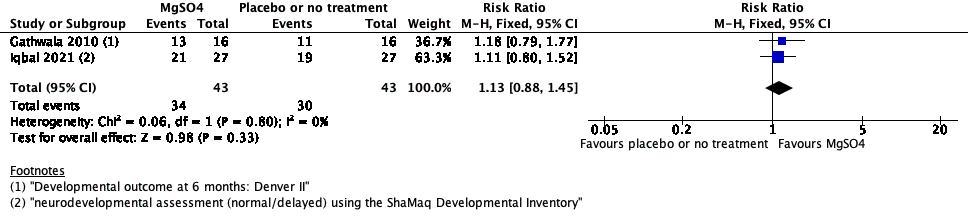


**Figure 1.53**: Forest plot of comparison: 1 MgSO_4_ versus placebo or no treatment, outcome: 1.53 Normal development at 6 months.


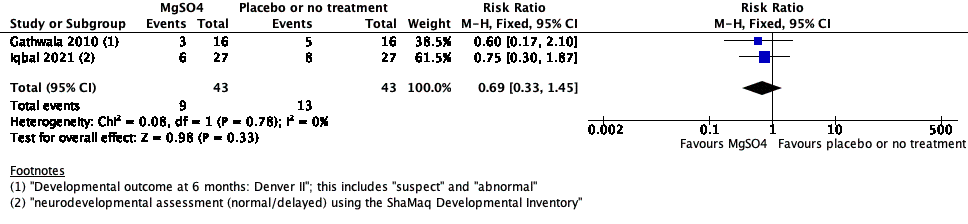


**Figure 1.54**: Forest plot of comparison: 1 MgSO_4_ versus placebo or no treatment, outcome: 1.54 Developmental delay at 6 months.


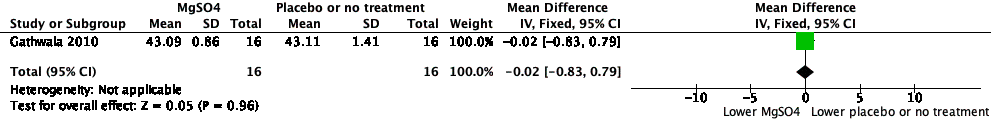


**Figure 1.55**: Forest plot of comparison: 1 MgSO_4_ versus placebo or no treatment, outcome: 1.55 Occipitofrontal circumference at 6 months (cm).


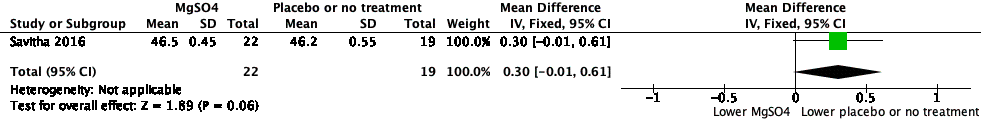


**Figure 1.56**: Forest plot of comparison: 1 MgSO_4_ versus placebo or no treatment, outcome: 1.56 Head circumference at 12 months (cm).


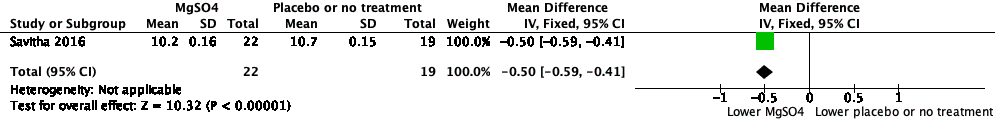


**Figure 1.57**: Forest plot of comparison: 1 MgSO_4_ versus placebo or no treatment, outcome: 1.57 Weight at 12 months (kg).


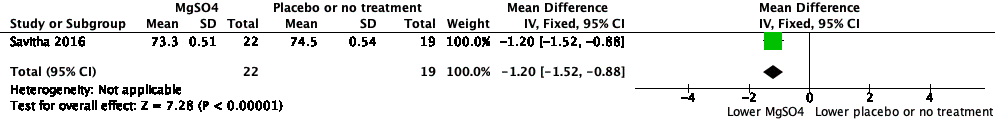


**Figure 1.58**: Forest plot of comparison: 1 MgSO_4_ versus placebo or no treatment, outcome: 1.58 Length at 12 months (cm).


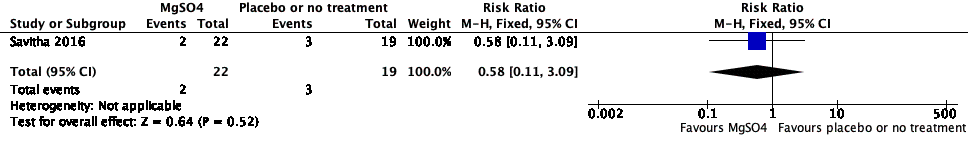


**Figure 1.59**: Forest plot of comparison: 1 MgSO_4_ versus placebo or no treatment, outcome: 1.59 Antiepileptic drugs for seizures at 12 months.


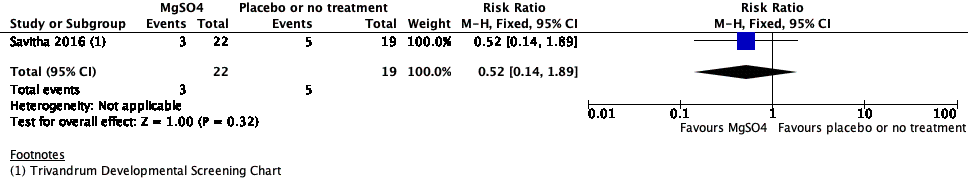


**Figure 1.60**: Forest plot of comparison: 1 MgSO_4_ versus placebo or no treatment, outcome: 1.60 Developmental delay at 12 months.


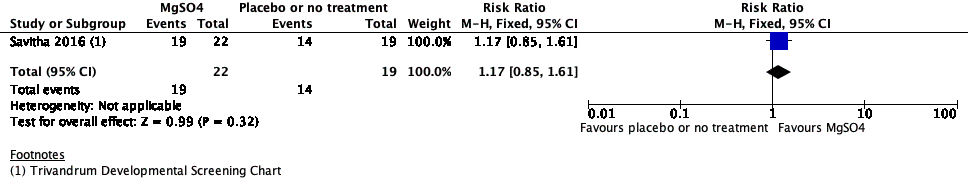


**Figure 1.61**: Forest plot of comparison: 1 MgSO_4_ versus placebo or no treatment, outcome: 1.61 Normal development at 12 months.


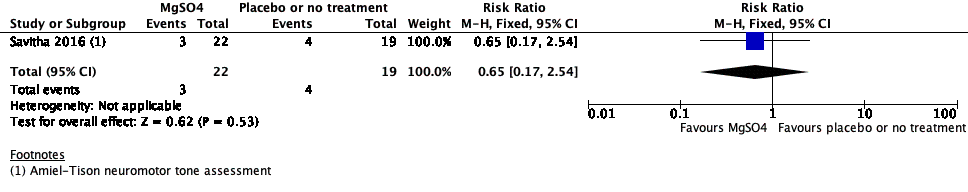


**Figure 1.62**: Forest plot of comparison: 1 MgSO_4_ versus placebo or no treatment, outcome: 1.62 Abnormal neuromotor tone at 12 months.


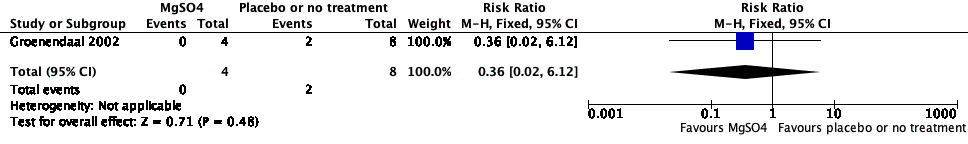


**Figure 1.63**: Forest plot of comparison: 1 MgSO_4_ versus placebo or no treatment, outcome: 1.63 Griffiths’ developmental quotient < 85 (among survivors) at 24 months.


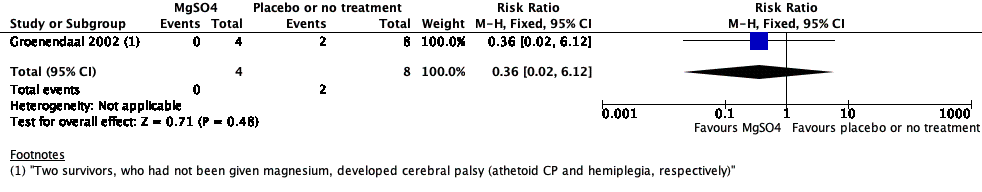


**Figure 1.64**: Forest plot of comparison: 1 MgSO_4_ versus placebo or no treatment, outcome: 1.64 Cerebral palsy (among survivors) at 24 months.


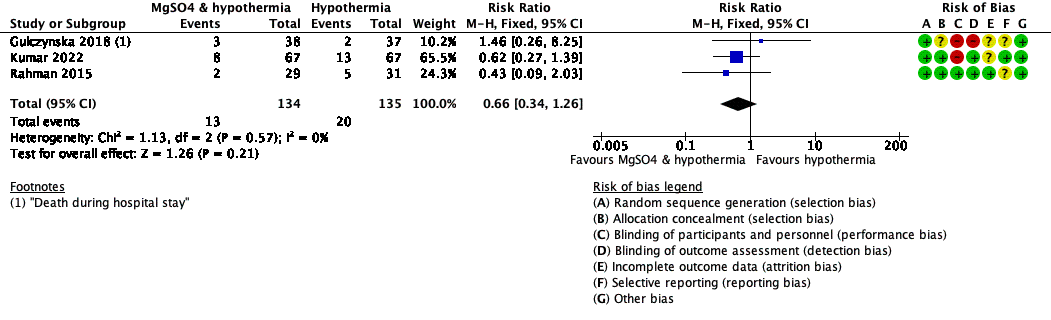


**Figure 2.1**: Forest plot of comparison: 2 MgSO_4_ and TH versus TH alone, outcome: 2.1 Neonatal death.


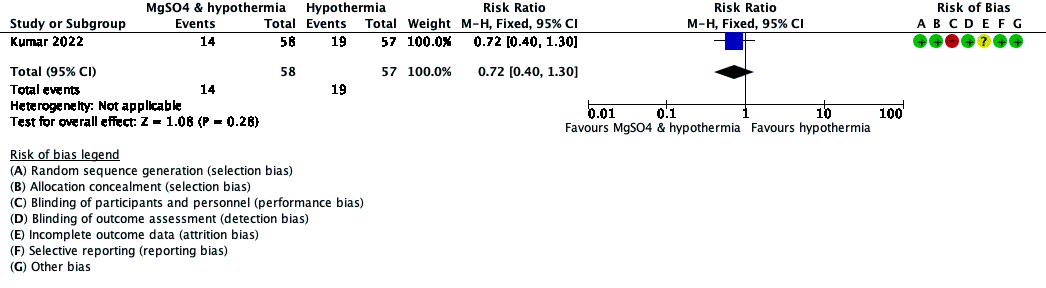


**Figure 2.2**: Forest plot of comparison: 2 MgSO_4_ and TH versus TH alone, outcome: 2.2 Neonatal death or DASII score < 70 at 12 months.


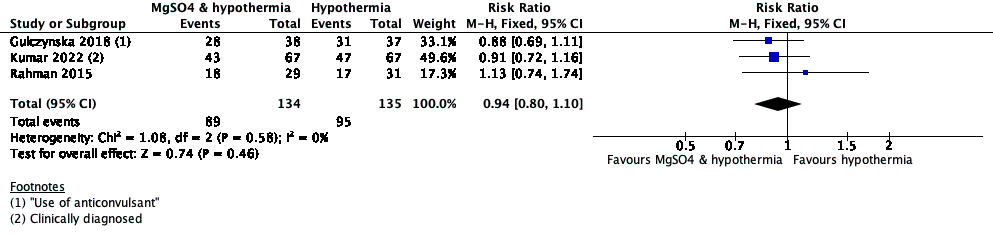


**Figure 2.3**: Forest plot of comparison: 2 MgSO_4_ and TH versus TH alone, outcome: 2.3 Seizures.


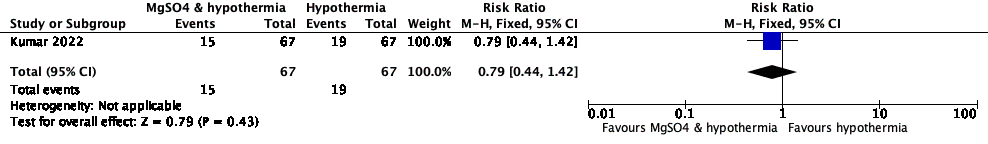


**Figure 2.4**: Forest plot of comparison: 2 MgSO_4_ and TH versus TH alone, outcome: 2.4 Seizures requiring > 1 anti-seizure medication.


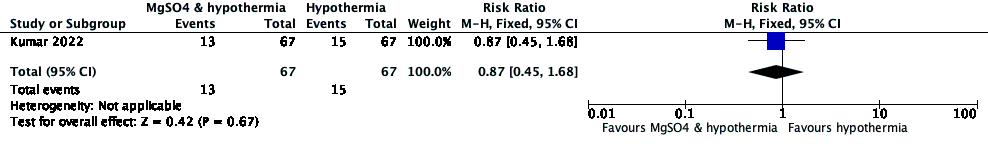


**Figure 2.5**: Forest plot of comparison: 2 MgSO_4_ and TH versus TH alone, outcome: 2.5 Anti-seizure therapy at discharge.


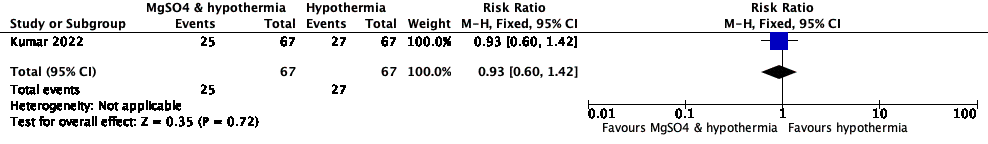


**Figure 2.6**: Forest plot of comparison: 2 MgSO_4_ and TH versus TH alone, outcome: 2.6 Abnormal neurological status at discharge (assessed by Hammersmith neonatal neurological examination).


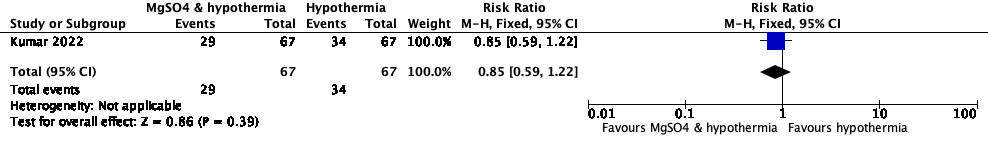


**Figure 2.7**: Forest plot of comparison: 2 MgSO_4_ and TH versus TH alone, outcome: 2.7 Respiratory depression requiring ventilator support.


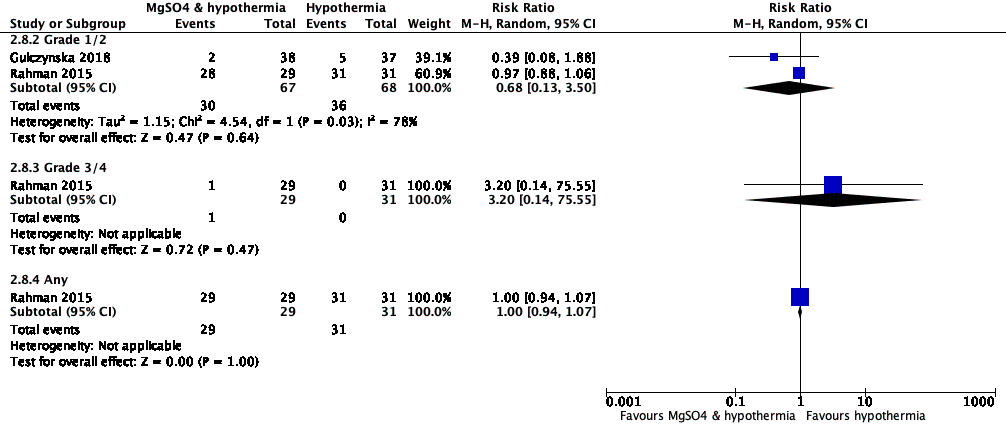


**Figure 2.8**: Forest plot of comparison: 2 MgSO_4_ and TH versus TH alone, outcome: 2.8 Intracranial haemorrhage.


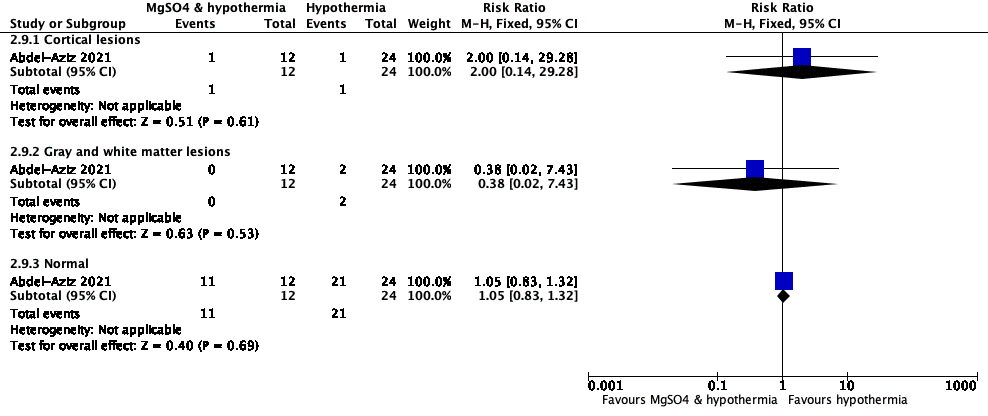


**Figure 2.9**: Forest plot of comparison: 2 MgSO_4_ and TH versus TH alone, outcome: 2.9 MRI findings.


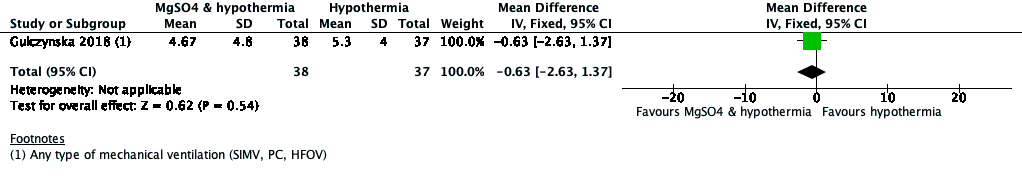


**Figure 2.10**: Forest plot of comparison: 2 MgSO_4_ and TH versus TH alone, outcome: 2.10 Length of mechanical ventilation (days).


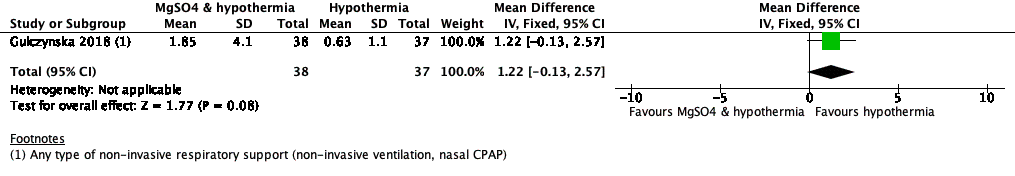


**Figure 2.11**: Forest plot of comparison: 2 MgSO_4_ and TH versus TH alone, outcome: 2.11 Length of non-invasive respiratory support (days).


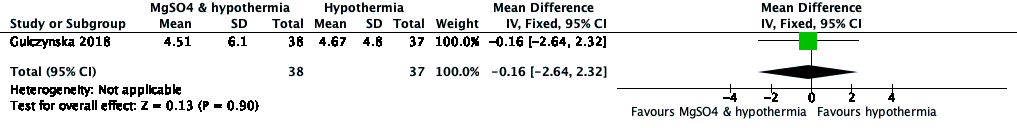


**Figure 2.12**: Forest plot of comparison: 2 MgSO_4_ and TH versus TH alone, outcome: 2.12 Length of oxygen supplementation (days).


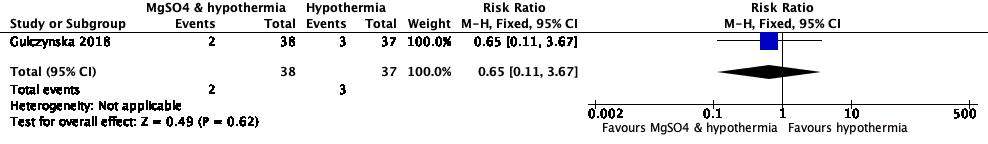


**Figure 2.13**: Forest plot of comparison: 2 MgSO_4_ and TH versus TH alone, outcome: 2.13 Inhaled nitric oxide treatment.


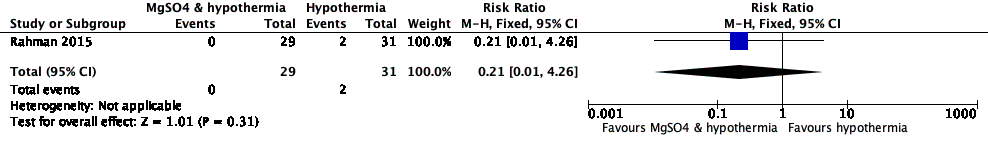


**Figure 2.14**: Forest plot of comparison: 2 MgSO_4_ and TH versus TH alone, outcome: 2.14 Pulmonary air leak syndrome.


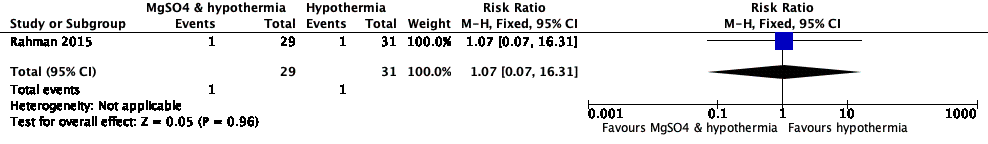


**Figure 2.15**: Forest plot of comparison: 2 MgSO_4_ and TH versus TH alone, outcome: 2.15 Pulmonary haemorrhage.


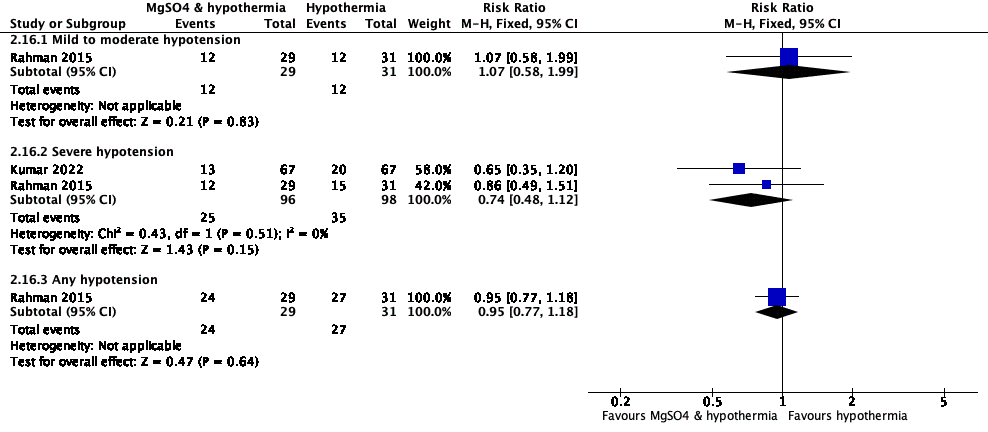


**Figure 2.16**: Forest plot of comparison: 2 MgSO_4_ and TH versus TH alone, outcome: 2.16 Hypotension.


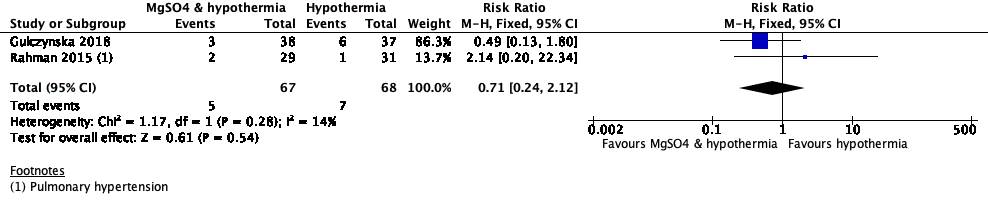


**Figure 2.17**: Forest plot of comparison: 2 MgSO_4_ and TH versus TH alone, outcome: 2.17 PPHN.


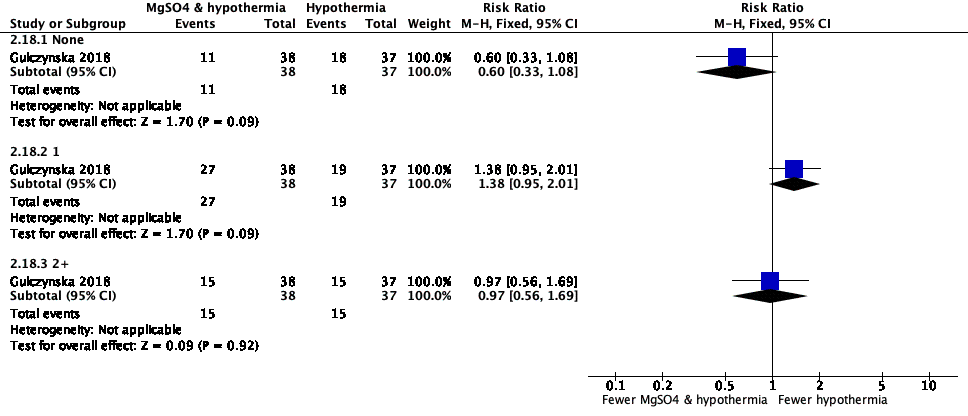


**Figure 2.18**: Forest plot of comparison: 2 MgSO_4_ and TH versus TH alone, outcome: 2.18 Catecholamine use.


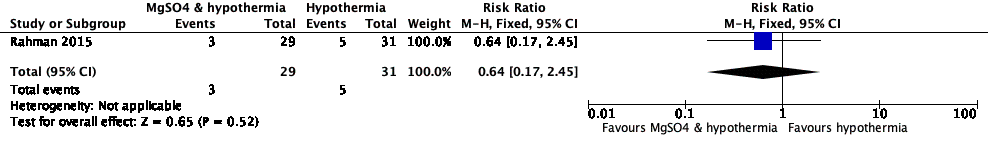


**Figure 2.19**: Forest plot of comparison: 2 MgSO_4_ and TH versus TH alone, outcome: 2.19 Meconium aspiration.


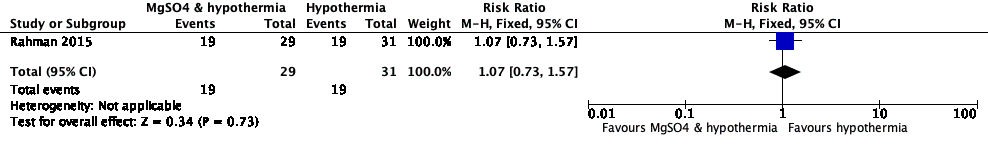


**Figure 2.20**: Forest plot of comparison: 2 MgSO_4_ and TH versus TH alone, outcome: 2.20 Raised LFTs.


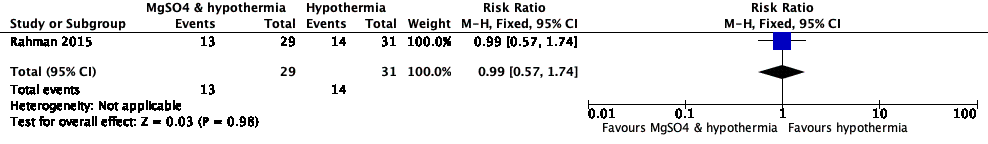


**Figure 2.21**: Forest plot of comparison: 2 MgSO_4_ and TH versus TH alone, outcome: 2.21 Renal failure.


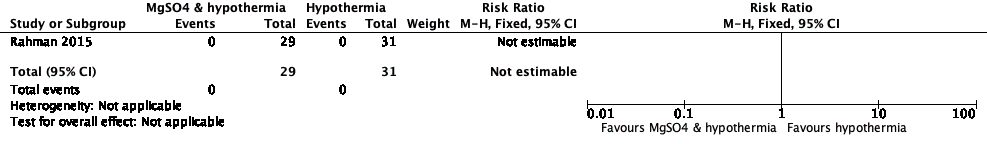


**Figure 2.22**: Forest plot of comparison: 2 MgSO_4_ and TH versus TH alone, outcome: 2.22 Major venous thrombosis.


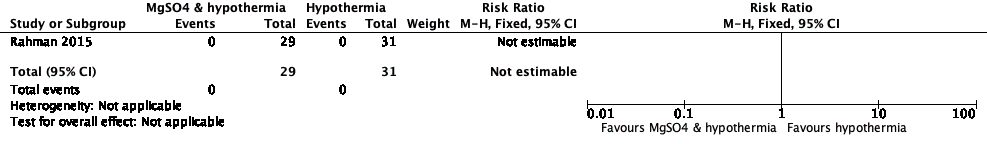


**Figure 2.23**: Forest plot of comparison: 2 MgSO_4_ and TH versus TH alone, outcome: 2.23 Subcutaneous fat necrosis.


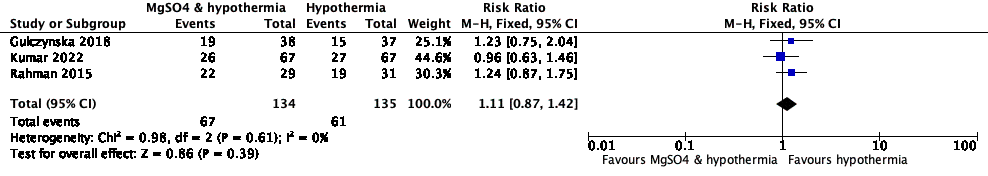


**Figure 2.24**: Forest plot of comparison: 2 MgSO_4_ and TH versus TH alone, outcome: 2.24 Thrombocytopenia.


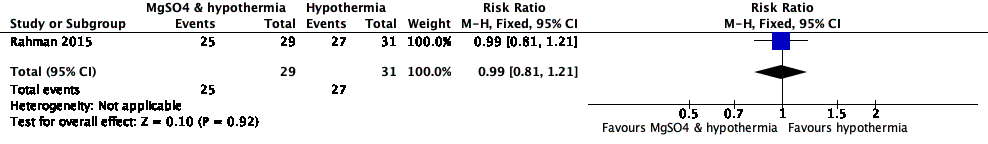


**Figure 2.25**: Forest plot of comparison: 2 MgSO_4_ and TH versus TH alone, outcome: 2.25 Prolonged coagulation.


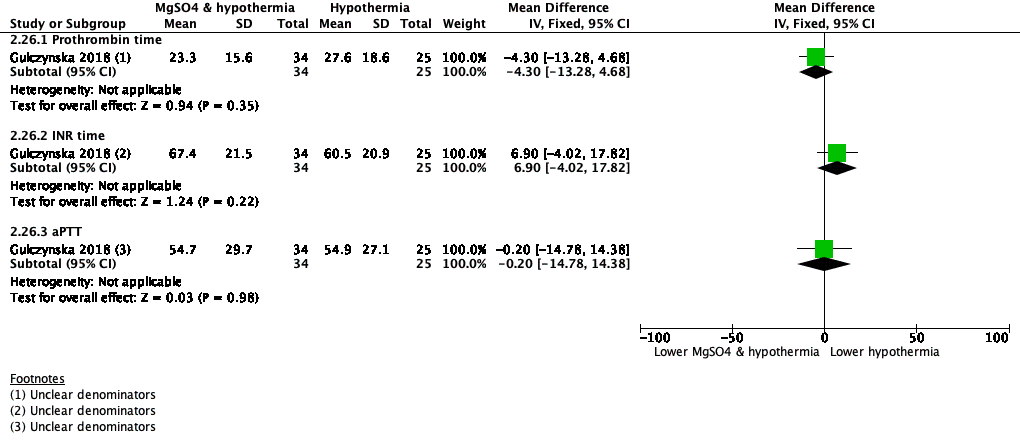


**Figure 2.26**: Forest plot of comparison: 2 MgSO_4_ and TH versus TH alone, outcome: 2.26 Coagulation parameters.


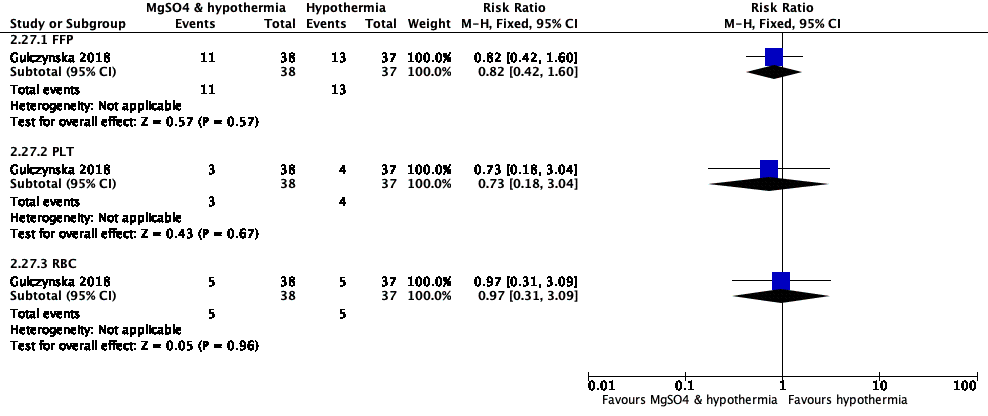


**Figure 2.27**: Forest plot of comparison: 2 MgSO_4_ and TH versus TH alone, outcome: 2.27 Transfusion.


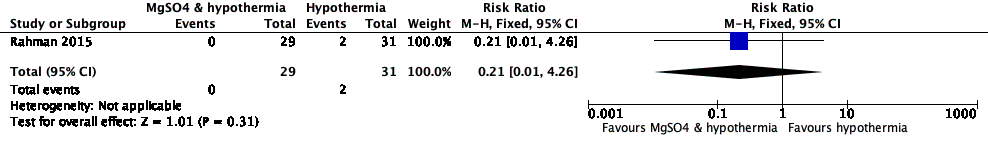


**Figure 2.28**: Forest plot of comparison: 2 MgSO_4_ and TH versus TH alone, outcome: 2.28 Necrotizing enterocolitis.


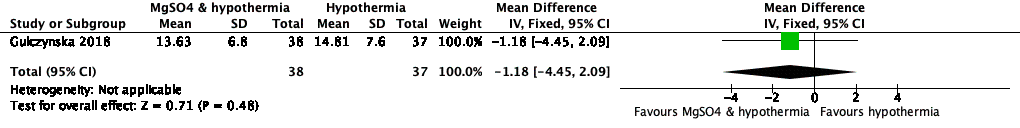


**Figure 2.29**: Forest plot of comparison: 2 MgSO_4_ and TH versus TH alone, outcome: 2.29 Length of antibiotic therapy (days).


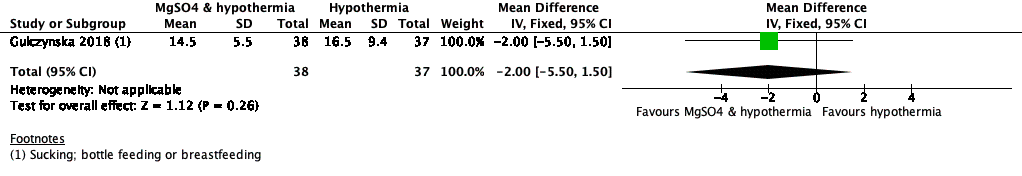


**Figure 2.30**: Forest plot of comparison: 2 MgSO_4_ and TH versus TH alone, outcome: 2.30 Time to full oral feedings (days).


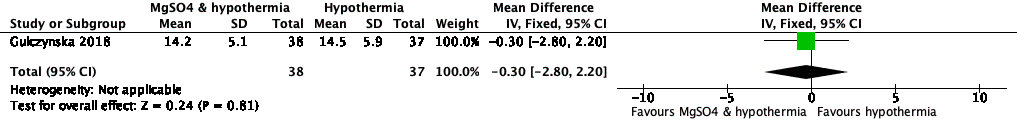


**Figure 2.31**: Forest plot of comparison: 2 MgSO_4_ and TH versus TH alone, outcome: 2.31 Time to full enteral feeding (days).


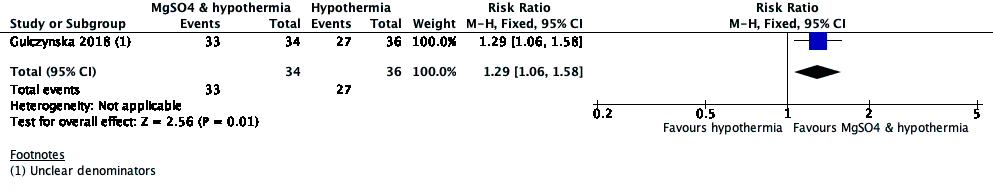


**Figure 2.32**: Forest plot of comparison: 2 MgSO_4_ and TH versus TH alone, outcome: 2.32 Full oral feedings on discharge.


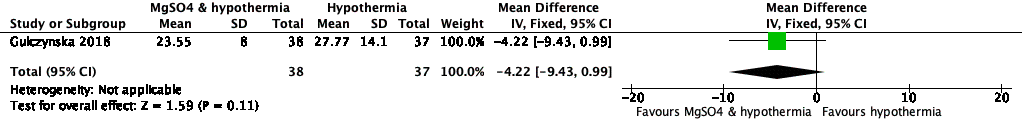


**Figure 2.33**: Forest plot of comparison: 2 MgSO_4_ and TH versus TH alone, outcome: 2.33 Length of hospitalisation (days).


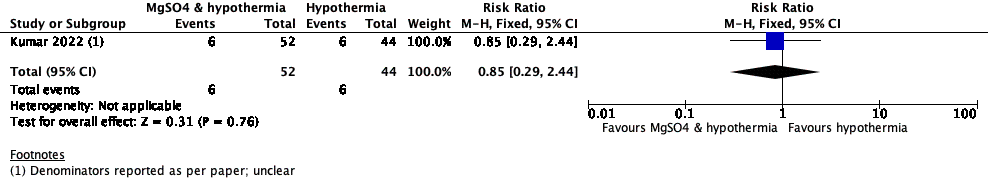


**Figure 2.34**: Forest plot of comparison: 2 MgSO_4_ and TH versus TH alone, outcome: 2.34 DASII score < 70 at 12 months.


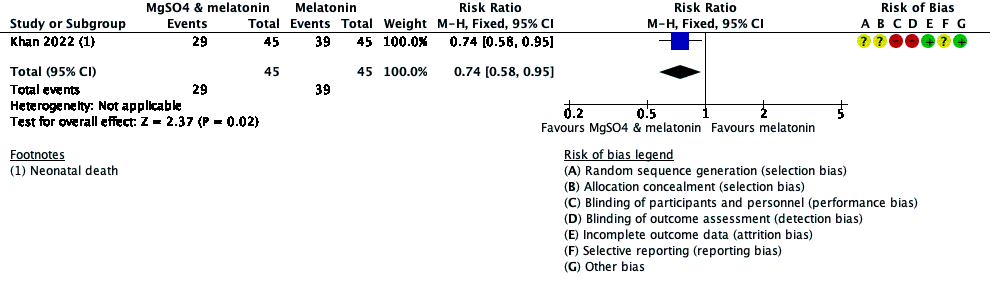


**Figure 3.1**: Forest plot of comparison: 3 MgSO_4_ and melatonin versus melatonin alone, outcome: 3.1 Neonatal death.

**Figure 3.2**: Forest plot of comparison: 3 MgSO_4_ and melatonin versus melatonin alone, outcome: 3.2 Seizures.

**Figure 3.3**: Forest plot of comparison: 3 MgSO_4_ and melatonin versus melatonin alone, outcome: 3.3 Intracranial haemorrhage.

**Figure 3.4**: Forest plot of comparison: 3 MgSO_4_ and melatonin versus melatonin alone, outcome: 3.4 Hypotension.

**Figure 3.5**: Forest plot of comparison: 3 MgSO_4_ and melatonin versus melatonin alone, outcome: 3.5 Renal failure.

**Figure 3.6**: Forest plot of comparison: 3 MgSO_4_ and melatonin versus melatonin alone, outcome: 3.6 Thrombocytopenia.

**Figure 3.7**: Forest plot of comparison: 3 MgSO_4_ and melatonin versus melatonin alone, outcome: 3.7 pH.

**Figure 4.1**: Forest plot of comparison: 4 MgSO_4_ versus phenobarbital, outcome: 4.1 Neonatal death.
