# Supplementary material for: Neonatal magnesium sulphate for neuroprotection: A systematic review and meta‐analysis
Source: Dev Med Child Neurol. 2024 Mar 11;66(9):1157–72. doi: 10.1111/dmcn.15899 (PMC11579813; doi:10.1111/dmcn.15899)
Supplement: Supplementary file 7 — Table S1: Characteristics of the randomized trials [file DMCN-66-1157-s008.docx]

**Table S1:** Characteristics of the randomised trials

| **Citation** | **Study design** | **Country; years recruited** | **Eligibility criteria** | **N** | **Intervention** | **Control** | **Primary review outcomes** | **Secondary review outcomes** | **Funding; conflicts** | **Notes** |
| --- | --- | --- | --- | --- | --- | --- | --- | --- | --- | --- |
| **MgSO_4_ versus placebo or no treatment** | | | | | | | | | |  |
| Ahmad 2019 | RCT | Pakistan; not reported | Inclusion: neonates admitted with birth asphyxia and “fits”; ≤ 6 hours post-birth  Exclusion: neonates with congenital heart diseases and multiple congenital anomalies | 260 | MgSO_4_ (N=130): 250 mg/kg ≤ 6 hours post-birth, and at 24 hours and 48 hours post-birth | No treatment (N=130) | Not reported | Control of fits; side effects* | Not reported; not reported | “preterm births were noted in 91 (35.0%) of our study cases” |
| Bhat 2009 | RCT | India  2004-2006 | Inclusion: ≥ 37 weeks’ gestation; < 6 hours of age at admission; with HIE (moderate or severe); and severe perinatal asphyxia – 3 of the following 4 criteria: 1) history of fetal distress; 2) need for immediate neonatal ventilation with bag and mask or ET for ≥ minutes post-birth; 3) Apgar score < 5 at 5 minutes; 4) base deficit ≥ 15 mEq/L or pH ≤ in cord blood or admission arterial blood samples ≤ 1 hour post-birth. Moderate or severe HIE was diagnosed when ≥ 1 sign present in 3 of 6 categories: 1) level of consciousness, lethargy (moderate) or stupor/coma (severe); 2) spontaneous activity decreased (moderate) or absent (severe); tone, hypotonia (moderate) or flaccidity (severe); posture, distal flexion or Moro reflex incomplete (moderate) or absent (severe); autonomic nervous system, pupils constricted, bradycardia, or periodic breathing (moderate) or pupils deviated/dilated/non-reactive to light, variable heart rate or apnoea (severe)  Exclusion: neonates with severe intrauterine growth restriction; any condition unrelated to asphyxia; aged > 6 hours at admission; maternal prenatal magnesium; metabolic disorder; chromosomal anomalies; congenital malformation | 40 | MgSO_4_ (N=20): 250 mg/kg IV (1 mL/kg in 20 mL 5% dextrose), 3 doses, 24 hours apart | Placebo (N=20): 1 mL/kg normal saline in 20 mL 5% dextrose, 3 doses, 24 hours apart | Neonatal death | Seizures (requiring anticonvulsants); EEG abnormalities at day 14; CT abnormalities at day 14 (any; basal ganglia injury; cortical injury; injury to both cortical and subcortical areas); assisted ventilation; hypotension requiring pressor support; persistent pulmonary hypertension; oliguria; renal failure; hepatic dysfunction; disseminated intravascular coagulation; established feedings (oral or tube) at day 7; oral feeding (sucking) at discharge; creatine phosphokinase; lactate dehydrogenase; good short-term neonatal outcome (composite measure of no neurologic abnormality, normal neuroimaging, normal EEG, and oral feedings at discharge); abnormal neurologic examination findings at discharge; blood pressure and apnoea* | Not reported; not reported | - |
| Gathwala 2010 | RCT | India; 2000-2001 | Inclusion: inborn, term, appropriate for gestational age neonates, admitted to NICU with Apgar score < 3 at 1 minute and < 6 at 5 minutes.  Exclusion: neonates with congenital malformations; maternal receipt of MgSO_4_, pethidine, phenobarbitone or other treatments “likely to depress the baby.” | 40 | MgSO_4_ (N=20): 250 mg/kg IV (in 5% dextrose) over 30 minutes ≤ 30 minutes of birth, followed by 125 mg/kg at 24- and 48-hours post-birth | No treatment (N=20) | Neonatal death | Seizures; refractory seizures; EEG abnormalities; CT abnormalities (focal, multifocal, or diffuse hypodensities); normal development at 6 months; developmental delay at 6 months (Denver II, normal, suspect, abnormal); occipitofrontal circumference at 6 months; tolerance (heart rate, oxygen saturation, respiratory rate, mean arterial pressure)* | None; none | 2 publications related to RCT |
| Groenendaal 2002 | RCT | The Netherlands; 1995-1998 | Inclusion: asphyxiated full-term neonates; admitted between 2-3 hours post-birth to the NICU; perinatal asphyxia defined, as combination ≥ 3 of: signs of fetal hypoxia (abnormal fetal heart rate patterns); pH < 7.1 in umbilical arterial blood; need for resuscitation at birth; Apgar score ≤ 5 at 10 minutes; need for intermittent positive pressure ventilation at 10 minutes.  Exclusion: not reported | 22 | MgSO_4_ (N=8): concentration 500 mg/mL (2.0 mmol/L), 0.5 mL/kg IV over 30 minutes [equivalent to 250 mg/kg]; 0.25 mL/kg at 24 and 48 hours later [equivalent to 125 mg/kg] (solutions diluted with normal saline to total volume 8 mL) | Placebo (N=14): NaCl 0.9%, 0.5 mL/kg IV over 30 minutes; 0.25 mL/kg at 24 and 48 hours later (solutions diluted with normal saline to total volume 8 mL) | Neonatal death | Clinical seizures; use of anticonvulsant drugs (0, 1, 2, 3, 4); suppression of aEEG background pattern before administration (3 hours) and 12 hours; CUS subcortical echodensities ≤ 4 hours of birth; composite neonatal adverse outcome (including death); Griffiths’ developmental quotient < 85 (among survivors) at 24 months; cerebral palsy (“criteria of Hagberg et al.” (among survivors) at 24 months; blood pressure, heart rate, spontaneous activity, ventilator settings*; use of cardiosupportive drugs*; dose or timing of antiepileptic drugs*; aEEG recordings between 13 and 24 hours* | Not reported; not reported | 5/8 and 10/14 neonates in MgSO_4_ and placebo groups received phenobarbital (20 mg/kg) in the first 2 hours post-birth (prior to study). Study ceased early due to personal communication” “severe hypotension and death were reported in two severely asphyxiated neonates that had received higher doses of magnesium (M. I. Levene, pers. comm.)” |
| Hossain 2013 | RCT | Bangladesh; 2010 | Inclusion: term neonates; < 12 hours post-birth; history of perinatal asphyxia and HIE (moderate or severe)  Exclusion: not reported | 50 | MgSO_4_ (N=not reported): 250 mg/kg IV (0.5 mL/kg) for 3 doses, 24 hours apart | Placebo (N=not reported): normal saline IV, 3 doses, 24 hours apart | Neonatal death* (reports overall rate only) | Oral feedings at discharge; good short-term neonatal outcome; neurologic abnormalities at discharge; “alteration in colour, heart rate, respiration, capillary filling time/blood pressure and oxygen saturation”*; electroencephalographic abnormalities* | Not reported; not reported | Data taken from abstract only; unable to obtain full report (via authors and institution) |
| Ichiba 2002 | RCT; multi-centre (9 clinical, 1 coordinating) | Japan; 1998-2000 | Inclusion: neonates ≥ 37 weeks’ gestation; Apgar score ≤ 7 at 5 minutes; failure to initiate spontaneous respiration at ≤ 10 minutes post-birth due to asphyxia or the presence of clinically apparent seizures ≤ 24 hours post-birth; ability to undergo initial MgSO_4_ treatment ≤ 24 hours post-birth; parental consent  Exclusion: maternal prenatal magnesium treatment; hypotension (systolic blood pressure < 40 mmHg) despite dopamine and/or volume expander infusion; major central nervous system malformation; central nervous system infection; metabolic disorder | 34 | MgSO_4_ (N=18): 250 mg/kg IV over 1 hour; 2 further doses at 24 and 48 hours; and a dopamine infusion (5 μg/kg per minute) | No treatment (N=16): “The placebo drug was not administered.” | Neonatal death | Seizures; duration of seizures; EEG abnormalities (any; focal periodic lateralised epileptiform discharges; low voltage); CT abnormalities at day 14 (any, diffuse cortical injury; injury to basal ganglia and thalamus; injury to both cortex and deep grey structure); duration of assisted ventilation; assisted ventilation; established oral feedings at day 14; maximum creatine kinase; maximum lactate dehydrogenase; good short-term neonatal outcome (survival with normal CT, normal EEG and established oral feeding at day 14); blood pressure, heart rate, respiratory rate* | Not reported; not reported; | - |
| Iqbal 2021 | RCT | Pakistan; 2020 | Inclusion: neonates > 35 weeks’ gestation; < 24 hours of age at presentation; unable to initiate/sustain breathing at birth; with clinical features suggestive of encephalopathy (neurological depression; depressed respiratory drive; seizures); classified as moderate/severe according to “modified Sarnat and Sarnat staging”  Exclusion: neonates with sepsis; pneumonia; congenital heart disease; inborn error of metabolism; congenital malformations | 70 | MgSO_4_ (N=31): 250 mg/kg IV (in 10% dextrose to make volume 3.0 mL/kg) over 1 hour, at 0, 24 and 48 hours | Placebo (N=31): 10% dextrose to make 3.0 mL/kg over 1 hour at 0, 24, 48 hours | Neonatal death | Seizure control; cranial ultrasound abnormalities at discharge (diffuse or focal echogenicity and intracranial haemorrhage); initiation of feeds; duration of stay; developmental status at 6-month follow-up (ShaMaq Developmental Inventory – normal; delayed) | Not reported; none | Length of hospital stay notably short |
| Khashaba 2006 | RCT | Egypt; not reported | Inclusion: ≥ 37 weeks’ gestation; asphyxiated neonates: Apgar score ≤ 3 at 5 minutes, and/or delayed first breath > 10 minutes post-birth. Severity of HIE determined during initial examination “according to Sarnat and Sarnat”  Exclusion: congenital anomalies; culture-proven sepsis or meningitis; suspected intracranial haemorrhage; maternal antenatal MgSO_4_ treatment | 47 | MgSO_4_ (N=23): 250 mg/kg IV over 10 minutes (10% solution) | Placebo (N=24): equal volume of isotonic saline (0.9%) IV over 10 minutes, ≤ 24 hours post-birth | Neonatal death | Apnoea at 72 hours; mechanical ventilation at 72 hours; need for oxygen at 72 hours; use of inotropic drips at 72 hours; need for anticonvulsants at discharge; CSF concentrations: aspartate at 72 hours; aspartate from baseline to 72 hours; TNF-α from baseline to 72 hours; glutamate concentration at 72 hours; glutamate from baseline to 72 hours; CSF concentrations at 72 hours (IL-1-β or IL-6)*; haemodynamic changes*; significant side effects* | Not reported; not reported | Data taken from 2 publications |
| Kumar 2015 | RCT | India; 6-month period | Inclusion: term (37-42 weeks’ gestation) and post-term (> 42 weeks’ gestation) neonates; moderate to severe perinatal asphyxia with HIE stage II or above (“Sarnat & Sarnat classification”); admitted ≤ 6 hours post-birth  Exclusion: not reported | 50 | MgSO_4_ (N=25): 250 mg/kg IV (0.5 mL/kg/dose, 50% w/v diluted in 5 mL/kg 5% dextrose, infused at 25 mg/minute), 3 doses, 24 hours apart, started ≤ 6 hours post-birth | No treatment (N=25): “did not receive this treatment” | Neonatal death | Seizures (continued at 24 hours); established feedings at day 7 (able to feed orally); composite neonatal adverse outcome (including death); neurological abnormalities at discharge (“NeoNeuro& Up scoring chart by Ellison”); haemodynamic parameters and respiration* | None; none | - |
| Mehmood 2015 | RCT | Pakistan; 2013 | Inclusion: term neonates; aged < 6 hours of either sex; moderate or severe HIE. Severe perinatal asphyxia characterised: history of fetal distress; need for immediate ventilation ETT or bag and mask > 2 minutes post-birth; Apgar score < 6 at 5 minutes; base deficit > 15 mEq/L or pH < 7 in arterial blood gases post-birth. Moderate or severe HIE established ≥ 1 sign present in 3 of 6 categories (level of consciousness; spontaneous activity; tone; posture; primitive reflexes; autonomic nervous system – pupils, heart rate, breathing pattern)  Exclusion: severe intrauterine growth restriction; condition unrelated to asphyxia; aged > 6 hours at presentation; maternal parenteral magnesium perinatally; chromosomal anomalies; congenital malformations | 70 | MgSO_4_ (N=35): 250 mg/kg IV (1 mL/kg in 20 mL 5% dextrose) over 1 hour at 0, 24, 48 hours | Placebo (N=35): 1 mL/kg normal saline in 20 mL 5% dextrose at 0, 24, 48 hours | Not reported | CT abnormalities at day 14; oral feedings at discharge (suck reflex); “good short term outcomes at discharge, the composite measure of all parameters”* | Not reported; not reported | - |
| Mullalli-Bime 2016 | RCT | Author from Albania; not reported | Inclusion: term neonates, ≥ 37 weeks’ gestation; severe perinatal asphyxia  Exclusion: not reported | 32 | MgSO_4_ (N=16): 250 mg/kg IV (1 mL/kg), 3 doses, 24 hours apart | Placebo (N=16): normal saline IV (1 mL/kg), 3 doses, 24 hours apart | Neonatal death | Abnormal findings on neuroimaging (transfontanellar sonogram) on day 14; oral feedings at discharge; good short-term outcome at discharge; neurological abnormalities at discharge | Not reported; not reported | Data taken from conference abstract only; (numbers calculated from percentages) |
| Nanda 2022 | RCT | India; 2019-2022 | Inclusion: neonates with moderate to severe perinatal asphyxia (preterm, term, post-term), with 1 of the following: Apgar score < 3 at 1 minute, or < 7 at 5 minutes, or with moderate to severe encephalopathy (lethargy, stupor, coma, abnormal reflexes, weak suck, or seizure ≤ 24 hours post-birth with a history of delayed cry at birth)  Exclusion: neonates with evident congenital malformations or proven chromosomal disorders; with proven inborn errors of metabolism; parents who refused consent | 227 | MgSO_4_ (N=116): 250 mg/kg IV (over 1 hour, in 20 mL 5% dextrose) at admission, and 24 and 48 hours after the first dose | No treatment (N=111): “standard treatment alone” | Neonatal death | Use of anticonvulsant drugs (1, 2, 3, 4); number of seizures (1, 2-5, > 5); oxygen required; shock (requiring inotropes); number of inotropes required (dobutamine; dobutamine and dopamine; dobutamine, dopamine and adrenaline); antibiotics required; oral feedings at discharge; OGT feeding at discharge; final outcome (discharge); time to initiation of first feeds*; time to full feeds*; NIBP at 72 hours*; duration of hospital stay* | Not reported; not reported | Several outcomes reported according to age at enrolment (< 6 hours, 6-24 hours, > 24 hours); some preterm neonates included |
| Rashid 2015 | RCT | Pakistan; not reported | Inclusion: ≥ 36 weeks’ gestation; perinatal asphyxia with neonatal encephalopathy “stage II & III”  Exclusion: neonates who were growth restricted; congenital anomalies; presenting > 6 hours post-birth; maternal administration of MgSO_4_ in labour | 200 | MgSO_4_ (N=100): 250 mg/kg/dose IV (in 20 mL saline) over 1 hour (syringe pump), commencing ≤ 6 hours post-birth, then at 24 and 48 hours later | Placebo (N=100): 1 mL/kg per dose normal saline infusion at the same time points (0, 24, 28 hours) (reported elsewhere, as 20 mL per dose) | Neonatal death | Oral feeding at discharge; NG feeds at day 14 of life | Not reported; not reported | - |
| Riyaz Ahmed 2016 | RCT | India; 2007-2009 | Inclusion: term neonates; appropriate for gestational age; severe birth asphyxia (Apgar score ≤ 3 at 1 minute and ≤ 6 at 6 minutes); supportive evidence: umbilical cord arterial blood pH ≤ 7  Exclusion: preterm; small or large for gestational age; maternal receipt of general anaesthesia during caesarean; maternal receipt of MgSO_4_ for pregnancy induced hypertension; maternal treatment with pethidine, phenobarbitone or other sedative treatments; gross congenital malformation; intracranial haemorrhage; pathological jaundice; clinical/laboratory evidence of intrauterine infection; abnormal head size at birth (< 5^th^ or > 95^th^ percentile) | 80 | MgSO_4_ (N=40): 250 mg/kg IV (in 5% dextrose) ≤ 30 minutes of birth over 30 minutes, followed by 125 mg/kg at 24 and 48 hours | No treatment (N=40) | Neonatal death | HIE (I, II, III, “as described by Sarnat and Sarnat”); seizures; apnoea at 72 hours; respiratory rate*; blood pressure* | None; none | - |
| Sajid 2018 | RCT | Pakistan; 2011 | Inclusion: neonates of either sex; > 37 weeks’ gestation; HIE (moderate to severe); severe birth/perinatal asphyxia; with a CT scan brain  Exclusion: neonates with intrauterine growth restriction; maternal prenatal magnesium; congenital malformation; with CT brain showing congenital brain malformations | 66 | MgSO_4_ (N=33): 250 mg/kg IV, 3 doses, 24 hours apart | Placebo (N=33): normal saline IV (1 mL/kg), 3 doses, 24 hours apart | Not reported | CT brain at discharge (normal; abnormal); oral feeds (sucking) at discharge (established; not established); neurological abnormalities at discharge (not improved; improved) | Not reported; not reported | - |
| Savitha 2016 | RCT | India; 2011-2012 | Inclusion: neonates at term, with perinatal asphyxia: a sentinel hypoxic event immediately before/during birth; failure to initiate breath at birth or Apgar score < 7 at 1 minute; need for resuscitation (positive pressure/chest compression) at birth; early onset features of HIE; exclusion of other aetiologies for encephalopathy  Exclusion: maternal MgSO_4_ prior to birth; maternal pethidine or phenobarbitone; obvious external congenital malformations | 120 | MgSO_4_ (N=60): 250 mg/kg IV (1 mL/kg/dose in 20 mL 5% dextrose) over 1 hour ≤ 6 hours of birth, with additional doses after 24 and 48 hours | Placebo/control (N=60): 3 doses of normal saline (1 m:/kg/dose) (in 20 mL 5% dextrose), 24 hours apart | Neonatal death; death or neurodevelopmental disability at 12 months | Seizures; duration of seizures; seizure control (with 1 anticonvulsant; requiring > 1 anticonvulsant); duration of seizures (< 48 hours); assisted ventilation; shock; acute kidney injury; duration for initiation of feeding (nasogastric tube; Paladai; direct breast); oral feeding at discharge; duration of recovery from neurological abnormalities; recovery from abnormal neurological examination ≤ 4 days; anticonvulsant required at discharge; normal neuromotor tone (Amiel Tison criteria) at discharge; neurologic status normal/improved at discharge; normal neuroimaging at discharge; head circumference at 12 months; weight at 12 months; length at 12 months; antiepileptic drugs for seizures at 12 months; developmental delay at 12 months (Trivandrum Developmental Screening Chart); abnormal neuromotor tone at 12 months; adverse effects*; heart rate, respiratory rate, blood pressure, oxygen saturation*; normal cranial ultrasound at 12 months* | None; none | Data taken from 3 publications; 2 papers present the same results, one as a “post hoc subgroup analysis” (neonates with moderate to severe HIE) |
| Siddiqui 2021 | RCT | Pakistan; 2019 | Inclusion: term (inborn/outborn) neonates arriving ≤ 6 hours of birth; with HIE, defined as: need for neonatal resuscitation at birth, with Apgar scores ≤ 3 at 1 minute and ≤ 7 at 5 minutes; “Sarnat scoring” used to grade HIE severity  Exclusion: preterm neonates; neonates with dysmorphism, comorbidities, or arriving > 6 hours post-birth | 82 | MgSO_4_ (N=41): 250 mg/kg IV (in 10 mL, 10% dextrose), over 30 minutes at admission, 24 and 48 hours | No treatment (N=41) | Neonatal death | Seizures present; frequency of seizures (single); frequency of seizures (multiple); seizures < 48 hours; seizures > 48 hours; suck feed at discharge; neurological status normal at discharge; side effects* | Not reported; none | - |
| Singh 2015~ (CTRI/2015/08/006107) | RCT | India; 2014-2015 | Inclusion: neonates > 37 weeks’ gestation; aged < 6 hours at admission; severe perinatal asphyxia  Exclusion: neonates with severe intrauterine growth restriction; any condition unrelated to asphyxia; aged > 6 hours at admission; maternal disorder; chromosomal anomalies or congenital malformation | 50 | MgSO_4_ (N=unclear): 250 mg/kg IV (in 20 mL, 5% dextrose over 1 hour) per day for 3 days | Placebo (N=unclear): 3 doses of normal saline, 1 mg/kg (in 20 mL, 5% dextrose) per day for 3 days | Not reported | Early control of seizure*; early appearance of normal cry*; early appearance of normal activity*; early acceptance of full oral feed by sucking*; “neuroprotective”* | “Source of Monetary of Material Support… M.L.B. Medical college, Jhansi”; not reported | Data taken from trial registration report only; unable to obtain full report (via authors) |
| **MgSO_4_ and TH versus TH alone** | | | | | | | | | |  |
| Abdel-Aziz 2021 | RCT | Egypt; 2019-2020 | Inclusion: neonates with moderate to severe HIE (“Sarnat modified score system”); ‘physiologic criteria’: ≥ 36 weeks’ gestation, admitted to NICU with ≥ 1 of: Apgar score at 5 minutes post-birth of 5; continued requirement for resuscitation via mask/ET at 10 minutes post-birth; acidosis ≤ 60 minutes post-birth (arterial, umbilical cord, or capillary pH < 7.1 or base deficit ≥ 16 mmol/L); manifestations of fetal distress before birth (meconium-stained amniotic fluid, tachycardia, or bradycardia); ‘neurological criteria’: changes of consciousness (lethargy, stupor, coma) with ≥ 1 of: clinical seizures; abnormal pupillary reflexes; abnormal oculomotor reflex; absent or weak suckling; absent or weak Moro reflex, and hypertonia  Exclusion: preterm neonates; neonates with congenital anomalies; neonates born to mothers who received treatments that caused neonatal depression (e.g., phenobarbitone or pethidine) | 36 | MgSO_4_ and TH (N=12): 250 mg/kg IV (with dopamine (5 μg/kg per minute)) over 1 hour per day for 3 days; and whole-body cooling ≤ 6 hours post-birth for 72 hours | TH alone (N=12): as described  Standard care (N=12): supportive treatment only | Not reported | MRI findings at discharge (normal, cortical lesions, grey and white matter lesions); duration of respiratory support and mechanical ventilation*; frequency of convulsions; initiation of feeding*; antiepileptic drugs at discharge*; | None; none | Data for control groups combined for review outcomes |
| Gulczynska 2018 | RCT; multi-centre (HEMEN) | Poland; not reported | Inclusion: gestational age > 36 weeks; acute perinatal sentinel event (asphyxia) confirmed by Apgar score ≤ 5 at 10 minutes and/or umbilical cord blood or any blood during the first hour post-birth with pH < 7.1 and/or base deficit ≥ 16; need for mechanical ventilation ≥ 10 minutes post-birth; and admission ≤ 6 hrs post-birth. Neonates meeting criteria underwent ‘Sarnat grading scale’, and those with moderate or severe HIE were further screened with aEEG – those with moderate or severe abnormalities were eligible  Exclusion: neonates with major congenital malformation; or with extremely poor prognosis (Apgar score 0 at 15 minutes or later | 75 | MgSO_4_ and TH (N=38): 250 mg/kg IV (1 hour infusion; 20%, 2 g/10 mL), 3 doses, 24 hours apart; and TH (selective head (rectal temperature: 34.0-35.0°C) or whole body (rectal temperature: 33.0-34.0)) for 72 hours, followed by rewarming at 0.5°C/hour | TH alone (N=37): as described | Neonatal death | Seizures (use of anticonvulsant); thrombocytopenia; intracranial haemorrhage (grade ½); PPHN; catecholamine use (none; 1; ≥ 2; any); length of mechanical ventilation; length of non-invasive respiratory support; length of oxygen supplementation; length of antibiotic therapy; time to full enteral feeding; time to full oral feedings (sucking – bottle or breastfeeding); length of hospitalisations; coagulation parameters (prothrombin time; INR time; aPTT); inhaled nitric oxide treatment; RBC transfusions; FFP transfusions; PLT transfusions; full oral feeds on discharge; RBC transfusion (/patient)*; FFP transfusions (/patient)*; PLT transfusion (/patient)*; doses of anticonvulsant (/patient)*; heart rate*; arterial blood pressure*; bradycardia*; hypotension*; need for vasopressors*; FiO2*; MAP*; platelet count*; HIE scores (‘Thompson scale’)* | Funding: NN407547538 form Polish Ministry of Education and Science; none | 2 additional conference abstracts identified (2012, 2014); no reply from author to confirm if relate to same RCT |
| Kumar 2022 | RCT | India; 2018-2019 | Inclusion: term neonates; umbilical arterial blood gas or postnatal venous blood gas ≤ 1 hour showing pH ≤ 7 or base deficit ≥ 12 mEq/L; moderate or severe encephalopathy based on “modified Sarnat and Sarnat criteria”; if blood gas not available, included based on: Apgar score ≤ 5 at 10 minutes or assisted ventilation for ≥ 10 minutes post-birth, with a history of acute perinatal event (intrapartum fetal distress, uterine rupture, cord prolapse, placental abruption)  Exclusion: aged ≥ 6 hours at randomisation; major congenital abnormalities; maternal receipt of MgSO_4_ | 134 | MgSO_4_ and TH (N=67): 250 mg/kg IV (50% w/v concentration, colourless solution, 500 mg/mL; dose diluted in 10 mL normal saline, administered over 30 minutes: 8 mg/kg/minute) ≤ 6 hours post-birth, with 2 subsequent doses at 24 hours intervals; and TH: whole body cooling (to attain rectal temperature 33°C-34°C) for 72 hours, with rewarming at a rate of 0.5°C/hour to reach the target temperature of 36.5°C | TH alone (N=67): as described | Neonatal death; neonatal death or DASHII score < 70 at 12 months | Seizures; seizures requiring > 1 anti-seizure medication; respiratory depression requiring ventilator support; hypotension (severe); abnormal neurological status at discharge (Hammersmith neonatal neurological examination); thrombocytopenia; anti-seizure therapy at discharge; DASHII score < 70 at 12 months; days of antiseizure therapy*; time to attain full oral feeding*; duration of ventilation*; oxidative stress markers (malondialdehyde and total antioxidant status)* | Funding: “Jawaharlal Institute of Postgraduate Medical Education and Research (JIPMER) intramural grant (JIPMER/01/134/2019/01009)”; none | - |
| Rahman 2015 | RCT; multi-centre (Mag Cool) | Qatar, Turkey, Saudi Arabia, Egypt, Malaysia, and United Arab Emirates; 2012-2013 | Inclusion: neonates ≥ 35 completed weeks’ gestation; moderate to severe HIE (“using the Sarnat and Sarnat criteria”); meeting ‘Criteria A’ (condition at birth): ≥ 1 of: Apgar score < 5 at 10 minutes due to birth asphyxia/perinatal depression; continued need for resuscitation (ET or mask) at 10 minutes post-birth; acidosis ≤ 60 minutes post-birth (umbilical cord, arterial, or capillary pH < 7), or base deficit (≥ -16 mmol/L in umbilical cord or any blood sample); and meeting ‘Criteria B’ (neurological assessment): evidence of moderate to severe encephalopathy: altered state of consciousness and ≥ 1 of: hypotonia; abnormal reflexes; absence of weak suck; clinical seizures; meeting ‘Criteria C’ (aEEG) was optional: ≥ 30 minutes aEEG recording showing abnormal background activity or seizures  Exclusion: neonates > 6 hours by randomisation; major congenital abnormalities or congenital abnormalities suggestive of chromosomal anomaly or other syndromes; very severe HIE where elective withdrawal is justified by attending clinician | 60 | MgSO_4_ and TH (N=29): 2.5 ml/kg/day (250 mg/kg, 10%) IV over 30 minutes, ≤ 6 hours of birth, with 3 doses in total (24 hours apart); and TH – either total body or head cooling, to a  core body temperature (rectal) of 33.5°C (range: 33.0-34.0°C) for 72 hours followed by slow re‐warming over a period of 8 hours at a rate not exceeding 0.5°C/hour | Placebo and TH (N=31): 2.5 mL/kg/day normal saline, IV over 30 minutes, ≤ 6 hours of birth, with 3 doses in total (24 hours apart); and TH, as described | Neonatal death | Seizures; hypotension (mild to moderate; severe; any); thrombocytopenia; renal failure; raised LFTs; prolonged coagulation; meconium aspiration; intracranial haemorrhage (grade < 3; grade ≥ 3; any); pulmonary air leak syndrome; necrotising enterocolitis; pulmonary haemorrhage; pulmonary hypertension; major venous thrombosis; subcutaneous fat necrosis | “funded by the Hamad Medical Corporation Internal Grants Competition (IG 1028)”; none | “Totally, 55 babies (91.7%) were cooled by total body cooling… and five babies (8.3%) by head cooling…” |
| **MgSO_4_ and melatonin versus melatonin alone** | | | | | | | | | |  |
| El Farargy 2020~ | RCT | Authors from Egypt; not reported | Inclusion: neonates with moderate HIE (“Sarnat grade II”)  Exclusion: not reported | 60 | MgSO_4_ and melatonin (N=30) | Melatonin alone (N=30) | Not reported | S100-B (marker of brain injury)* | Not reported; not reported | Data taken from abstract only; unable to obtain full text (via author or institution) |
| Khan 2022 | RCT | Pakistan; 2020-2021 | Inclusion: neonates with Apgar score < 5 at 5 minutes, umbilical blood pH < 7.0, and moderate neonatal encephalopathy (“modified Sarnat score”)  Exclusion: neonates with congenital abnormalities; intrauterine growth restriction; sepsis; born to mothers with diabetes mellitus type II | 90 | MgSO_4_ and melatonin (N=45): MgSO_4_ 250 mg/kg IV over 60 minutes at days 0, 1 and 3; in addition to melatonin 10 mg/kg enteral daily for 5 consecutive days | Melatonin only (N=45): melatonin as described | Neonatal death | Seizures; thrombocytopenia; renal failure; hypotension (mild to moderate; severe; total); intracranial haemorrhage (< grade 3; ≥ grade 3; total); pH (3 and 7 days) | Not reported; none | Gestational age range reported: 36-38 weeks; death rate notably high |
| **MgSO_4_ versus phenobarbital** | | | | | | | | | |  |
| Riaz 2021 | RCT | Pakistan; 2020-2021 | Inclusion: neonates aged 1-3 days old; either gender; presenting with birth asphyxia  Exclusion: neonates with major birth defects or congenital deformities, unstable vital signs (Apgar < 5 at 5 minutes) and very low birth weight (< 1000 grams) | 104 | MgSO_4_ (N=52): 250 mg/kg IV (diluted in 5 mL/kg/dose 5% dextrose, infused at 25 mg/minute), 3 doses, 24 hours apart | Phenobarbital (N=52): 20 mg/kg IV over 15 minutes, titrated to response; with further loading doses of 5 mg/kg given if seizures recurred up to a maximum of 40 mg/kg | Neonatal death (reported variably as “neonatal mortality” and “Adverse outcome”) | Not reported | Not reported; not reported | Preterm neonates included: mean (SD) gestation at birth, MgSO_4_ (37.87 ± 2.29); phenobarbital (37.15 ± 2.39); excludes neonates with Apgar < 5 at 5 minutes |

~Studies do not contribute data to main results/meta-analyses.

*Data for outcomes not able to be included in main results/meta-analyses; results summarised in Supplemental Table 14.

Abbreviations: aEEG: amplitude integrated electroencephalography; aPPT: activated partial thromboplastin time; CSF: cerebrospinal fluid; CT: computerised tomography; CUS: cranial ultrasound; DASHII: Developmental Assessment Scales for Indian Infants; EEG: electroencephalogram; ET: endotracheal; ETT: endotracheal tube; FFP: frozen fresh plasma; FiO2: fraction of inspired oxygen; HIE: hypoxic ischaemic encephalopathy; IL-1-β: interleukin-1 beta; IL-6: interleukin-6; INR: international normalised ratio; IV: intravenous; LFTs: liver function tests; MAP: mean arterial pressure; mEq/L: milliequivalents per litre; MgSO4: magnesium sulphate; mg/kg: milligrams per kilogram; mg/mL: milligrams per millilitre; mL: millilitre; mL/kg: millilitres per kilogram; mmHg: millimetres of mercury; mmol/L: millimole per litre; MRI: magnetic resonance imaging; N: number of participants; NaCl: sodium chloride; NG: nasogastric; NIBP: non-invasive blood pressure; NICU: neonatal intensive care unit; OGT: orogastric tube; pH: potential hydrogen; PLT: platelet; PPHN: persistent pulmonary hypertension of the newborn; RBC: red blood cell; RCT: randomised controlled trial; SD: standard deviation; TH: therapeutic hypothermia; TNF-α: tumour necrosis factor alpha; w/v: weight by volume; μg/kg: micrograms per kilogram.
