# Supplementary material for: Neonatal magnesium sulphate for neuroprotection: A systematic review and meta‐analysis
Source: Dev Med Child Neurol. 2024 Mar 11;66(9):1157–72. doi: 10.1111/dmcn.15899 (PMC11579813; doi:10.1111/dmcn.15899)
Supplement: Supplementary file 8 — Table S2: Characteristics of the non‐randomized studies [file DMCN-66-1157-s013.docx]

**Table S2:** Characteristics of the non-randomised studies

| **Citation** | **Study design** | **Country; years recruited** | **Eligibility criteria** | **N** | **Group 1** | **Group 2** | **Primary review outcomes** | **Secondary review outcomes** | **Funding; conflicts** |
| --- | --- | --- | --- | --- | --- | --- | --- | --- | --- |
| Levene 1995 | Non-randomised trial | 5 European centres (authors from UK, Sweden Norway, and Finland); years not reported | Inclusion: ≥ 35 weeks’ gestation; < 12 hours post-birth; no major congenital malformations; 10-minute Apgar score < 6-, or 5-minute Apgar score < 6 with evidence of fetal distress defined as any of: sustained episodes of fetal bradycardia < 100/minute, thick meconium stained liquor, scalp pH of < 7.11, or umbilical arterial pH of < 7.00 or umbilical base deficit greater than -10 mmol/L; with consent for MgSO_4_ from parent(s)  Exclusion: not detailed | 15 | Higher dose MgSO_4_ (N=7): 400 mg/kg IV, over 10-30 minutes | Lower dose MgSO_4_ (N=8): 250 mg/kg IV over 10 minutes | Not reported | Mean arterial pressure, respiratory depression, EEG readings, heart rate | Funding: Laerdal Foundation for Acute Medicine and the Norwegian Association for Public Health; not reported |
| Okonkwo 2018 | Non-concurrent cohort study (described as “cross sectional survey of cases”) | Nigeria; 19-month period (prospective); and 2009-2014 historical controls (retrospective) | Inclusion: inborn or outborn neonates, with a history of fetal distress, meconium-stained amniotic fluid requiring resuscitation with bag-mask-valve device or endotracheal intubation during resuscitation; Apgar score at 1 minute ≤ 3 or at 5 minutes < 7 and presence of signs of HIE; metabolic acidosis, multisystem organ dysfunction  Exclusion: maternal treatment with MgSO_4_; neonates with obvious congenital anomalies or features of congenital heart disease | 711 | Prospective (N=100): MgSO_4_ 250 mg/kg IV (over 15-30 minutes) ≤ 30 minutes of admission, every 24 hours for 3 doses | Retrospective (N=611): “pre-magnesium treatment era” | Neonatal death | None | No funding; no conflicts |
| Sreenivasa 2017 | Non-randomised trial | India; 2015-2016 | Inclusion: term neonates; admitted to the NICU; appropriate for gestational age (weight); 1 minute Apgar score < 3- and 5-minute Apgar score < 6  Exclusion: congenital malformations; maternal receipt of general anaesthesia, MgSO_4_ and drugs likely to depress the baby (e.g., pethidine or phenobarbitone) | 100 | MgSO_4_ (N=50): 250 mg/kg IV (1 mL/kg/dose in 20 mL 5% dextrose) over 1 hour ≤ 6 hours post-birth, repeated 24- and 48-hours post-birth | Control (N=50) | Neonatal death | Seizures; seizures controlled with 1 anticonvulsant; seizures continued at 24 hours; duration of recovery from neurological abnormalities; duration for initiation of feeding: nasogastric tube feeding, spoon feeding, direct breast feeding; neurological findings at discharge: abnormal neurological findings, normal neuromotor tone (Amiel Tison criterial), normal neuroimaging; heart rate, oxygen saturation, respiratory rate; adverse effects | Personal funding; no conflicts |
| Szemraj 2005 | Non-concurrent cohort study | Not reported (authors from Poland) | Inclusion (MgSO_4_ group): asphyxiated neonates at > 36 weeks’ gestation; with absence of major congenital malformations; 10-minute Apgar score < 6-, or 5-minute Apgar score < 6- and 10-minute Apgar score > 6 if the following present: fetal bradycardia < 100/minute, green amniotic fluid, umbilical artery blood pH < 7.11, umbilical artery blood base excess < -10 mmol/L  Inclusion (control group 1): asphyxiated neonates  Inclusion (control group 2): healthy (Apgar ≥ 8) with similar gestational age | 45 | MgSO_4_ (N=18): 250 mg/kg IV over 30 minutes, in first 2 hours post-birth | Control 1 (N=7): no MgSO_4_  Control 2 (N=20) | Not reported | ATPase activities in erythrocyte membranes; protein kinases A and C in erythrocyte membranes; immunocharacteristics of band 3 in erythrocyte membranes | Supported in part by grants No. 5P05E 09224 from the State Committee for Scientific Research, Poland, No. 502-16-197 and No. 503 from the Medical University of Lodz; not reported |

Abbreviations: ATPase: P-type adenosine triphosphatase; EEG: electroencephalogram; HIE: hypoxic ischaemic encephalopathy; IV: intravenous; MgSO4: magnesium sulphate; mg/kg: milligrams per kilogram; mL/kg: millilitres per kilogram; mmol/L: millimole per litre; N: number of participants; NICU: neonatal intensive care unit; pH: potential hydrogen; UK: United Kingdom.
