# Supplementary material for: Neonatal magnesium sulphate for neuroprotection: A systematic review and meta‐analysis
Source: Dev Med Child Neurol. 2024 Mar 11;66(9):1157–72. doi: 10.1111/dmcn.15899 (PMC11579813; doi:10.1111/dmcn.15899)
Supplement: Supplementary file 9 — Table S3: Risk of bias of randomized trials [file DMCN-66-1157-s007.docx]

**Table S3:** Risk of bias of randomised trials

| **Citation** | **Random sequence generation (selection bias)** | **Allocation concealment (selection bias)** | **Blinding of participants and personnel (performance bias)** | **Blinding of outcome assessment (detection bias)** | **Incomplete outcome data (attrition bias)** | **Selective reporting (reporting bias)** | **Other bias** |
| --- | --- | --- | --- | --- | --- | --- | --- |
| Abdel-Aziz 2021 | Unclear risk: Reported inconsistently: “The patients were separated into two subgroups before randomly assigning them to moderate and severe HIE, then each group divided into three groups. The researchers randomly assigned the neonates into three groups according to severity of HIE, priority of admission and available places in NICU by utilizing random number tables generated by computer according to the inclusion and exclusion characteristics;” and “They were divided equally into three groups.” | Unclear risk: No further detail. | High risk: “Not any of the NICU’s nurses or the attending neonatologists was aware of this randomization;” however no placebo was used. | High risk: No blinding reported for outcome assessment. | Low risk: No apparent missing data or exclusions. | High risk: Limited outcome data reported; reported incompletely in Figures and text (format not suitable for inclusion in meta-analyses) | High risk: Baseline differences reported between groups for pregnancy-related risk factors (e.g., pre-eclampsia, antepartum haemorrhage). |
| Ahmad 2019 | Unclear risk: Unclear; “Patients were randomly divided in 2 groups (by draws method).” | Unclear risk: No further detail. | High risk: No placebo was used. | High risk: No blinding reported for outcome assessment. | Low risk: No apparent missing data or exclusions. | High risk: Data reported for 1 outcome (control of fits) only (and side effects mention in text). | Unclear risk: Limited methodological detail provided to further assess other bias; data provided assumes even randomisation (N=130 per group), though not clearly reported. |
| Bhat 2009 | Low risk: Quote: “assigned randomly, with computer-generated random numbers.” | Unclear risk: No further detail provided. | Low risk: Placebo; “The investigators and caregivers were blinded to the assignment of patients… Serum magnesium levels were concealed from the clinicians caring for these neonates.” | Low risk: Placebo; “The neurologic examination was performed by certified clinicians who were blinded to the group assignment of the patients.” | Low risk: No apparent missing data or exclusions. | Unclear risk: No trial registration or protocol available to further assess. | Low risk: No other obvious sources of bias. |
| El Farargy 2020 | Unclear risk: Unclear; “is a randomized controlled trial… Infants were randomized into 2 groups.” | Unclear risk: Insufficient detail in abstract to assess. | Unclear risk: Insufficient detail in abstract to assess. | Unclear risk: Insufficient detail in abstract to assess. | Unclear risk: Insufficient detail in abstract to assess. | Unclear risk: Insufficient detail in abstract to assess. | Unclear risk: Insufficient detail in abstract to assess. |
| Gathwala 2010 | Low risk: Quote: “These babies were randomized…using a random number table.” | Unclear risk: No further detail. | High risk: No placebo; considered unlikely. | Low risk: Blinding reported for some outcomes; unclear for follow-up: “The lab personnel were blinded to the group to which the patient belonged;” and “The CT scan and the EEG were evaluated by examiners who were blinded to the groups to which the babies belonged.” | Low risk: No apparent missing data or exclusions. | Unclear risk: No trial registration or protocol available to further assess. | Low risk: No other obvious sources of bias. |
| Groenendaal 2002 | Unclear risk: Insufficient detail: “Randomization was performed in one block of 30… Since randomization was performed in one block, an unequal distribution of magnesium and placebo-treated neonates was created when the trial was halted.” | Unclear risk: No further detail. | Low risk: Placebo; “double blind…Trial solutions were prepared by the hospital pharmacy… Blood samples were taken at 1, 12, 24 and 48 h for magnesium measurement, but the results were not available for the clinicians.” | Low risk: Placebo; “The effects of administration of the trial solution on aEEG and outcome were analysed by the authors, who were unaware of the contents of the trial solution.” | Low risk: No apparent missing data or exclusions, other than 1 neonate in placebo group lost to follow up. | High risk: Results reported incompletely in text for some outcomes, e.g., “(data not shown).” | Low risk: No other obvious sources of bias. |
| Gulczynska 2018 | Low risk: “Each of the participating centers received an individually designed randomization table prepared by the central coordinating board.” | Unclear risk: No further detail. | High risk: No placebo; no further details provided regarding blinding of participants and personnel. | High risk: No details provided regarding blinding of outcome assessment. | Unclear risk: No apparent missing data or exclusions. | Unclear risk: Minimal detail confirmed regarding outcomes in “Clinical Trials Gov. ID: NCT02499393.”; no access to protocol to confirm further. Results of some analyses partially reported e.g., “P = NS”; or with no measure of variance (e.g., “RBC transfusion no/patient”). | Low risk: No other obvious sources of bias. |
| Hossain 2013 | Unclear risk: Quote: “Patients were assigned randomly”; insufficient detail in abstract to assess. | Unclear risk: Insufficient detail in abstract to assess. | Unclear risk: Quote: “single blind”; insufficient detail in abstract to assess. | Unclear risk: Insufficient detail in abstract to assess. | Unclear risk: Insufficient detail in abstract to assess; results reported for 19 and 18 infants in MgSO_4_ and placebo groups respectively (of 50 randomised). | Unclear risk: Insufficient detail in abstract to assess. | Unclear risk: Insufficient detail in abstract to assess. |
| Ichiba 2002 | Unclear risk: Quote: “selected into two groups randomally.” | Unclear risk: Unclear: “Infants studied were allocated to the treated or control group by randomization using sealed envelopes that were opened by the treating investigators who then confirmed the allocation codes, designated the treatment and sent group assignment data to the coordinating center.” | High risk: No placebo; “Thus, the treating investigators were not blinded to group assignments following entry.” | High risk: No placebo; no further detail provided; considered unlikely. | Low risk: No apparent missing data or exclusions, other than 1 neonate “randomized to the treated group was excluded because of protocol violations.” | Unclear risk: No trial registration or protocol available to further assess. | Low risk: No other obvious sources of bias. |
| Iqbal 2021 | Low risk: Drawing of lots: “Neonates were randomized equally to both groups (magnesium sulfate and placebo) through the lottery method.” | Low risk: Pharmacy controlled: “A randomization slip was picked and attached to the neonate’s file by the pharmacist for every neonate included in the study.” | Low risk: Placebo used; “The doctor and the nurse were blinded while the pharmacist was responsible to break the code in case of an adverse event.” | Unclear risk: “Cranial ultrasound was performed by the same sonologist.” No further detail for blinding of outcome assessment. | High risk: High rate of exclusion/loss to follow up: “A total of 70 neonates were initially included in the study; however, eight neonates were excluded after being diagnosed with sepsis, pneumonia, congenital heart disease, and an inborn error of metabolism;” thus 62/70 neonates initially included. Following 6 deaths and 2 further losses to follow up, 54/70 were included in the 6 month follow up. | Unclear risk: No trial registration or protocol available to further assess. Absence of clear outcome definitions. | Low risk: No other obvious sources of bias. |
| Khan 2022 | Unclear risk: Unclear: “A non-probability convenience sampling technique was employed for the recruitment of the participants;” and “They were randomly assigned to either of the groups.” | Unclear risk: No further detail. | High risk: No placebo; no further details provided regarding blinding of participants and personnel. | High risk: No details provided regarding blinding of outcome assessment. | Low risk: No apparent missing data or exclusions. | Unclear risk: No access to trial registration or protocol assess further. | Low risk: No other obvious sources of bias. |
| Khashaba 2006 | Unclear risk: Quote: “infants were randomly assigned.” | Low risk: “Randomization was done using sequentially numbered sealed envelopes.” | Low risk: Placebo used: “prospective double-blinded randomized controlled trial”; and “The study solution for each infant was delivered to the neonatal intensive care unit (NICU) in a non-distinguishable ampoule;” and “All personnel involved in providing care to the infant or performing the analysis in the laboratory were unaware of infants’ group assignment.” | Low risk: Blinding of outcome assessment with use of a placebo. | Low risk: No apparent losses or exclusions for clinical outcomes. For the CSF measures: “36 (18 in each group) were collected from the follow up tap. Eleven samples were not available because of technical difficulties (N=7) or due to gross contamination with blood (N=4);” thus data available for 77% of participant. | Unclear risk: No trial registration or protocol available to further assess. 2011 publication reports that the 2006 publication presented “examined the efficacy of a single dose of MgSO4 on neurodevelopmental outcomes at 6 mo of age”, which is untrue. | Low risk: No other obvious sources of bias. |
| Kumar 2015 | Unclear risk: “These patients were randomized into two groups.” | Unclear risk: No detail. | High risk: Described as “single-blind;” no further details provided. No placebo, thus blinding considered unlikely for participants/personnel. | Unclear risk: Described as “single-blind”; no further details provided. | Low risk: No apparent missing data or exclusions. | High risk: Very few outcomes reported; with limited detail regarding definitions, and some detail reported incompletely in text (“hemodynamic parameters and respiration”). | Unclear risk: Limited methodological detail provided to further assess other bias. |
| Kumar 2022 | Low risk: “Computer-generated random numbers were used for randomization by a person not involved in the study. Block randomization was done using blocks of 2, 4, and 6.” | Low risk: “Allocation of participants to specific groups was carried out using serially labelled opaque sealed envelopes.” | High risk: No placebo; “The clinical team was not blinded to the intervention due to the logistics involved.” | Low risk: “However, the person who did the development assessment and the biochemist involved were blinded to the allocation.” | Unclear risk: 9/67 MgSO_4_ and TH group and 10/67 TH alone group were lost to follow up, and not included in primary outcome analysis (14% loss to follow up overall). | Low risk: Trial registration provided, and outcomes appear to have been reported as per pre-specifications. | Low risk: No other obvious risk of bias identified. |
| Mehmood 2015 | Unclear risk: Unclear; “Patients were randomly divided into two groups A and B. | Unclear risk: No further detail provided. | Unclear risk: Saline placebo used; however, no further detail provided regarding blinding of participants and personnel. | Unclear risk: No further detail provided regarding blinding of outcome assessment. | Low risk: No apparent missing data or exclusions. | High risk: Only 2 outcomes reported (and not neonatal death); in the discussion a composite measure was discussed which was not reported in the main text. | High risk: Concerns regarding limited methodological detail provided, including regarding inclusion criteria; potential baseline imbalances; poor outcome pre-specification and definition. |
| Mullalli-Bime 2016 | Unclear risk: Quote: “randomly assigned.” | Unclear risk: Insufficient detail in abstract to assess. | Unclear risk: Use of a “placebo”; no further detail in abstract to assess. | Unclear risk: Insufficient detail in abstract to assess. | Unclear risk: Insufficient detail in abstract to assess. | Unclear risk: Insufficient detail in abstract to assess. | Unclear risk: Insufficient detail in abstract to assess. |
| Nanda 2022 | Low risk: “We followed individual participant randomization and allocation for the trial. Upon admission, the admission registration numbers of the babies who were assessed for eligibility and included in the trial were allocated into the intervention or control group using an R-software based computer program.” | Unclear risk: No further detail provided. | High risk: “We used a non-blinded randomized controlled design”; and “Our study had some limitations. The care giver did have knowledge of the intervention, and this could potentially introduce bias.” | Low risk: “However, we have attempted to address this by relying on a blinded data collector and clinical evaluator.” | High risk: For death/discharge/left against medical advice (final outcome), not all 'no treatment' babies were accounted for. Death numbers for both groups do not appear to match in table and figure provided. | Unclear risk: Means reported only with no measure of variance for several outcomes/characteristics. No trial registration or protocol available to further assess. | Unclear risk: Limited methodological detail provided to further assess other bias. |
| Rahman 2015 | Low risk: Use of a computer (net based) random number generator: “the babies were randomized using a net‐based randomization system... Each recruitment center had its own unique password and PIN code for using the system. Each randomization was immediately notified by E‐mail directly from the randomization website to the data coordinating center in Doha, Qatar. Each randomized baby received a unique randomization code with two letters and one number.” | Low risk: Central allocation (web-based): “provided by a London‐based independent clinical trials randomization company (sealed envelope).” | Low risk: Blinding of study participants and key study personnel (placebo): “The code was prescribed by the physician at a dose of 2.5 ml/kg/day q 24 h for three doses which would be equivalent to 250 mg/kg/dose of 10% MgSO4 or 2.5 ml/kg/dose of normal saline. Once the coded prescription was received by the pharmacist, he/ she un‐coded the prescription and dispensed the study medication in a sealed syringe labeled with the code and study number… The decoding of individual patients was done by the study statistician keeping the investigators blinded. For statistical analysis, the patients were grouped into Arm A (therapeutic hypothermia plus MgSO4) or Arm B (therapeutic hypothermia plus placebo).” | Low risk: Blinding of outcome assessment (placebo). | Low risk: No apparent missing data or exclusions. | Unclear risk: Insufficient information available to permit a judgement of low risk or high risk (no protocol available).  A search for the trial registration identified the following: <https://clinicaltrials.gov/ct2/show/NCT01646619>. There are several discrepancies between the registration and the reported trial – including regarding outcomes (primary outcome pre-specified in the trial registration: “Combined outcome of Mortality and Severe Neurodevelopmental Disability [ Time Frame: 18 - 24 months of age ].” | Low risk: No other obvious sources of bias. |
| Rashid 2015 | Unclear risk: Unclear; “Patients were randomly assigned in two groups” (abstract); and “Patients were divided in to two groups (group A & group B) each having equal no of patients that was 100...” (main text). | Unclear risk: No further detail. | Unclear risk: Saline used in control group at same intervals (0, 24, 48 hours); however, no further detail provided regarding blinding for participants/personnel. | Unclear risk: No further details provided regarding blinding of outcome assessment. | Low risk: No apparent missing data or exclusions. | High risk: Results reported for only 3 outcomes, and incompletely (e.g., “a significant difference”, with no effect measure/P value provided). | Unclear risk: Limited methodological detail provided to further assess other bias. |
| Riaz 2021 | Unclear risk: Unclear; “Then patients were divided in to two groups… neonates were randomly allocated into two groups.” | Unclear risk: No further detail. | High risk: Treatment regimens differed (no placebo); thus, considered unlikely. | High risk: Not reported and considered unlikely. | Low risk: No apparent missing data or exclusions for the 1 outcome reported. | High risk: Data reported for 1 outcome only (no clear definition provided). | High risk: Several discrepancies (such as in numbers of neonates; dates of study) between abstract and main text; and errors in Tables 2 and 3 for numbers of neonates with the “adverse outcome.” |
| Riyaz Ahmed 2016 | Low risk: Random number table: “Babies were randomly allotted to one of the study or the control groups, using the randomization table.” | Unclear risk: No further detail provided. | High risk: No placebo reported; considered unlikely in view of monitoring protocol reported for MgSO_4_ group. | High risk: Not reported; considered unlikely. | Low risk: No apparent missing data or exclusions. | High risk: Data reported for a limited number of outcomes; outcomes discussed in methods (e.g., anticonvulsants used; biochemical investigations) not in results; and incomplete reporting in text for other outcomes (e.g., respiratory rate and blood pressure). | Unclear risk: Baseline characteristics reported in abstract only. |
| Sajid 2018 | Low risk: Computer-generated random numbers: “Neonates were randomly divided into two groups; each group has 33 subjects, group-A and B by using random number tables generated by computer.” | Unclear risk: No further detail provided. | Unclear risk: Use of a saline placebo; no further details provided regarding blinding of participants and personnel. | Unclear risk: No details provided regarding blinding of outcome assessment. | Low risk: No apparent missing data or exclusions. | High risk: Very few outcomes reported (3; not neonatal death), with limited detail regarding definitions. | Unclear risk: Limited methodological detail provided; potential baseline imbalance (age at birth, hours). |
| Savitha 2016 | Low risk: Quote: “were assigned randomly, with computer generated random numbers.” | High risk: Quote: “The sequentially numbered allocation was not concealed.” | Unclear risk: Control group reported as “Comparison group” throughout methods, but as “placebo group” in methods. Quote: “Blinding was not done due to settings limitation. | Low risk: Use of placebo; and quote: “clinical assessments were done by investigator number 2 who was blinded for assignment of patients.” | High risk: Unclear denominators reported throughout results (in table 2 and text). Regarding follow up, it was reported that 41/69 infants (of those with moderate or severe HIE in initial neonatal hospitalisation) could be followed up (59%). From the MgSO_4_ group 22/60 had follow up data (10 did not have adequate data; 20 were lost to follow up; 8 had incomplete assessments); from the control/placebo group 19/60 had follow up data (8 did not have adequate data; 22 were lost to follow up; 11 had incomplete assessments); thus, the overall follow up rate was 34%. | Unclear risk: Some outcomes (heart rate, respiratory rate, BP, and oxygen saturation) reported incompletely “not statistically significant” in text; no trial registration or protocol available to further assess. | Unclear risk: Unclear two of the papers reported the same results, one presented as a “subgroup analysis” (group of neonates with moderate to severe HIE). |
| Siddiqui 2021 | Unclear risk: Unclear: “Lottery method was used for randomisation. | Unclear risk: No further detail. | High risk: No placebo; blinding considered unlikely. | Unclear risk: “This was a single-blinded study.” No further detail reported re: blinding of outcome assessment. | Unclear risk: 2 neonates (1 in each group) left against medical advice; 80/82 analysed. For frequency and duration of seizures results reported do not account for all neonates. | Unclear risk: Manuscript provides trial registration number (“Clinical Registry No. NCT04705142”); details do not appear to correspond to trial reported (e.g., sample size of 200; single group assignment); the registration was retrospective. | Unclear risk: Quote: “the weight for gestation was significantly lower in the control group (p-value <0.05; Table 1).” |
| Singh 2015 | Unclear risk: Quote from registration: “Computer generated randomization.” | Unclear risk: Quote from registration: “Sequentially numbered, sealed, opaque envelopes.” | Unclear risk: Quote from registration: “Participant, Investigator and Outcome Assessor Blinded.” | Unclear risk: Insufficient detail in registration to assess. | Unclear risk: Insufficient detail in registration to assess. | Unclear risk: Insufficient detail in registration to assess; primary outcome listed as “neuroprotective” at 1 year, and secondary outcome as “not hypotensive at given dose” at 1 year; results reported varied. | Unclear risk: Insufficient detail in registration to assess. |

Abbreviations: aEEG: amplitude integrated electroencephalography; CSF: cerebrospinal fluid; CT: computerised tomography; EEG: electroencephalogram; h: hours; HIE: hypoxic ischaemic encephalopathy; mg/kg: milligrams per kilogram; MgSO_4_: magnesium sulphate; mo: months; N: number of participants; NICU: neonatal intensive care unit; NS: non-significant; RBC: red blood cell.
