# Supplementary material for: Neonatal magnesium sulphate for neuroprotection: A systematic review and meta‐analysis
Source: Dev Med Child Neurol. 2024 Mar 11;66(9):1157–72. doi: 10.1111/dmcn.15899 (PMC11579813; doi:10.1111/dmcn.15899)
Supplement: Supplementary file 10 — Table S4: Risk of bias of non‐randomized studies [file DMCN-66-1157-s004.docx]

**Table S4:** Risk of bias of non-randomised studies

| **Citation** | **Selection bias (and confounding)** | **Confounding** | **Performance bias** | **Detection bias (and confounding)** | **Attrition bias (and detection bias)** | **Selective outcome reporting bias** | **Overall risk of bias** |
| --- | --- | --- | --- | --- | --- | --- | --- |
|  | Do the inclusion and/or exclusion criteria vary across groups?  Does the strategy for recruiting participants differ across groups?  Is the selection of the comparison group inappropriate? | Any attempt to balance the allocation between the groups or match groups? (confounding)  Were important confounding variables not taken into account? (confounding) | Does the study fail to account for important variations from the proposed protocol? | Was the outcome assessor not blinded?  Were valid and reliable measures implemented consistently? | Was the length of follow-up different across groups?  In cases of high or differential loss to follow-up, was the impact assessed? | Are any important primary outcomes missing from the results?  Are any important harms missing from the results? |  |
| Levene 1995 | No. | No (allocation by centre). Yes, not taken into account. | Cannot determine. | Yes, not blinded. Yes (though mean arterial pressure measured with oscillometer in 2/15 neonates). | No. Not applicable. | Cannot determine. No (though neonatal death not reported). | High |
| Okonkwo 2018 | Partially. Differs. Yes, inappropriate. | No. Yes, not taken into account. | Cannot determine. | Yes, not blinded. Yes (neonatal death only reported outcome). | Cannot determine. No applicable. | Cannot determine. Yes (neonatal death only reported outcome). | High |
| Sreenivasa 2017 | No. | No. Yes, not taken into account. | Cannot determine. | Yes, not blinded. Yes. | No. Not applicable. | Cannot determine (some incomplete reporting of results). No. | High |
| Szemraj 2005 | Yes. Differs. | No. Yes, not taken into account. | Cannot determine | Yes, not blinded. Cannot determine (for inclusion/exclusion criteria). | No. Not applicable. | Cannot determine. | High |
