# Supplementary material for: Neonatal magnesium sulphate for neuroprotection: A systematic review and meta‐analysis
Source: Dev Med Child Neurol. 2024 Mar 11;66(9):1157–72. doi: 10.1111/dmcn.15899 (PMC11579813; doi:10.1111/dmcn.15899)
Supplement: Supplementary file 11 — Table S5: Subgroup analyses for neonatal death, Comparisons 1 and 2 [file DMCN-66-1157-s012.docx]

**Table S5:** Subgroup analyses for neonatal death, Comparisons 1-2

| **Outcome and subgroup** | **RCTs** | **N** | **Method (I^2^)** | **RR (95% CI)** | **χ^2^, P, I²** |
| --- | --- | --- | --- | --- | --- |
| **Comparison 1: MgSO_4_ versus placebo or no treatment** | | | | | |
| 5.1 Neonatal death: gestational age | | | | | |
| 5.1.1 Preterm and term (≥ 35 weeks' gestation) | 3 | 489 | F (0%) | 0.49 (0.35, 0.69) | **7.30, 0.007, 86.3%** |
| 5.1.2 Term (≥ 37 weeks’ gestation) | 10 | 544 | F (0%) | 0.96 (0.68, 1.35) |  |
| 5.2 Neonatal death: moderate or severe HIE | | | | | |
| 5.2.1 Inclusion criteria, yes | 5 | 579 | F (0%) | 0.49 (0.36, 0.68) | **10.36, 0.001, 90.3%** |
| 5.2.2 Inclusion criteria, no | 8 | 454 | F (0%) | 1.11 (0.76, 1.61) |  |
| 5.3 Neonatal death: setting | | | | | |
| 5.3.1 LMIC | 11 | 978 | F (25%) | 0.65 (0.51, 0.83) | 2.24, 0.13, 55.3% |
| 5.3.2 HIC | 2 | 55 | F (0%) | 1.30 (0.54, 3.14) |  |
| 5.4 Neonatal death: timing | | | | | |
| 5.4.1 ≤ 30 minutes | 2 | 120 | F (0%) | 1.00 (0.41, 2.47) | **8.23, 0.04, 63.5%** |
| 5.4.2 ≤ 6 hours | 7 | 652 | F (2%) | 0.54 (0.40, 0.73) |  |
| 5.4.3 ≤ 24 hours | 4 | 202 | F (0%) | 1.16 (0.71, 1.90) |  |
| 5.4.4 > 24 hours | 1 | 27 | F (NA) | 0.27 (0.03, 2.25) |  |
| 5.5 Neonatal death: dose | | | | | |
| 5.5.1 250 mg/kg | 1 | 47 | F (NA) | 1.79 (0.86, 3.73) | **10.19, 0.006, 80.4%** |
| 5.5.2 250 mg/kg + 2 x 125 mg/kg | 3 | 142 | F (0%) | 1.06 (0.55, 2.05) |  |
| 5.5.3 3 x 250 mg/kg | 9 | 844 | F (0%) | 0.56 (0.42, 0.74) |  |
| **Comparison 2: MgSO_4_ and TH versus TH alone** | | | | | |
| 6.1 Neonatal death: gestational age | | | | | |
| 6.1.1 Preterm and term (≥ 35 weeks' gestation) | 2 | 135 | F (6%) | 0.73 (0.24, 2.22) | 0.06, 0.80, 0% |
| 6.1.2 Term (≥ 37 weeks’ gestation) | 1 | 134 | F (NA) | 0.62 (0.27, 1.39) |  |

Statistically significant effect estimates in bold.

Test for heterogeneity represented by I^2^ statistic.

Results of test for subgroup differences represented by χ^2^, P value and I² statistic.

Abbreviations: CI: confidence interval; F: fixed-effects; HIC: high-income country; HIE: hypoxic-ischaemic encephalopathy; kg: kilogram; LMIC: low or middle-income country; mg: milligram; MgSO4: magnesium sulphate; N: number of participants; NA: not applicable; RCTs: randomised controlled trials; RR: risk ratio; TH: therapeutic hypothermia.
