# Supplementary material for: Neonatal magnesium sulphate for neuroprotection: A systematic review and meta‐analysis
Source: Dev Med Child Neurol. 2024 Mar 11;66(9):1157–72. doi: 10.1111/dmcn.15899 (PMC11579813; doi:10.1111/dmcn.15899)
Supplement: Supplementary file 12 — Table S6: Additional data from randomized trials not able to be included in meta‐analyses [file DMCN-66-1157-s011.docx]

**Table S6:** Additional data from randomised trials not able to be included in meta-analyses

| **Citation** | **Outcome** | **Findings** | **Notes** |
| --- | --- | --- | --- |
| **MgSO_4_ versus placebo or no treatment** | | | |
| Ahmad 2019 | Side effects | “Magnesium Sulphate… having no side effect” | - |
| Bhat 2009 | Mean arterial pressure | “No significant decrease in mean arterial pressure occurred during the first 72 hours of life, during which 3 doses of magnesium sulfate were administered in the treatment group.” | - |
|  | Apnoea | “Two patients in the treatment group developed apnea during the second dose of magnesium sulfate, which necessitated intubation and temporary ventilatory support.” |  |
| Gathwala 2010 | Tolerance (heart rate, oxygen saturation, respiratory rate, mean arterial pressure) | “Magnesium infusion was well tolerated and there were no significant alterations in heart rate, oxygen saturation, respiratory rate or mean arterial pressure following magnesium infusion either with the 250 mg/kg or the 125 mg/kg doses.” | - |
| Groenendaal 2002 | Blood pressure, heart rate, spontaneous activity, ventilator settings | “not affected by giving magnesium… (data not shown)” | - |
|  | Use of cardio-supportive drugs | “Differences… between the two groups were not significant” |  |
|  | Dose or timing of antiepileptic drugs | “nor was there any difference” |  |
|  | aEEG recordings between 12 and 24 hours | “No significant changes occurred… in either group.” |  |
| Khashaba 2006 | CSF concentrations of cytokines at 72 hours (pg/mL) | “differences were not observed in IL-1-β or IL-6 (Fig.2)” | For cytokines, data not able to be extracted from figure |
|  | Hemodynamic changes | “did not observe any in the group of infants who received MgSO4.” |  |
|  | Significant side effects | “Of note, the dose of MgSO4 at 250 mg/kg used in this study was not associated with any…” |  |
| Kumar 2015 | Hemodynamic parameters and respiration | “No significant adverse effects of MgSO4 were observed at the used dosage on...” | - |
| Ichiba 2002 | Blood pressure, heart rate, respiratory rate | “No significant differences were noted… between the two groups” | - |
| Hossain 2013 | Neonatal death | “The overall mortality rate in our study was 26%” | Data taken from abstract only |
|  | Electroencephalographic abnormalities | “no significant difference” |  |
|  | Alteration in colour, heart rate, respiratory, capillary filling time/blood pressure, and oxygen saturation | “no significant difference” |  |
| Mehmood 2015 | Good short-term outcomes at discharge, the composite measure of all parameters | “were statistically significant…support the positive role of magnesium sulfate infusion” | - |
| Nanda 2002 | Initiation of first feeds (hours) | MgSO_4_ group: 6 hours at enrolment (N=71): 31.94; 6-24 hours at enrolment (N=30): 35.6; > 24 hours at enrolment (N=15): 32.66  No treatment group: 6 hours at enrolment (N=69): 50.15; 6-24 hours at enrolment (N=30): 47.7; > 24 hours at enrolment (N=12): 56.5  “The babies who received MgSO4 within 6 hours of life achieved earlier initiation of feeds (32 hours) as compared to control groups (63 hrs).” | No measures of variance; where not reported, assumed to be means |
|  | Full feeds (hours) | MgSO_4_ group: 6 hours at enrolment (N=71): 121.5; 6-24 hours at enrolment (N=30): 123.8; > 24 hours at enrolment (N=15): 120.0  No treatment group: 6 hours at enrolment (N=69): 156.7; 6-24 hours at enrolment (N=30): 159.5; > 24 hours at enrolment (N=12): 149.2 |  |
|  | Mean NIBP at 72 hours (mmHg)/hypotension | MgSO_4_ group: 6 hours at enrolment (N=71): 42; 6-24 hours at enrolment (N=30): 44; > 24 hours at enrolment (N=15): 39  No treatment group: 6 hours at enrolment (N=69): 38; 6-24 hours at enrolment (N=30): 40; > 24 hours at enrolment (N=12): 40  “The NIBP remained unchanged over 72 hours of magnesium sulphate administration. Some studies have reported hypotension associated with MgSO4 but we did not find any significant association in our study” |  |
|  | Mean duration of hospital stay (days) | MgSO_4_ group: 6 hours at enrolment (N=71): 9.09; 6-24 hours at enrolment (N=30): 10.04; > 24 hours at enrolment (N=15): 11.07  No treatment group: 6 hours at enrolment (N=69): 6.22; 6-24 hours at enrolment (N=30): 9.96; > 24 hours at enrolment (N=12): 5.88 |  |
| Riyaz Ahmed 2016 | Respiratory rate | “No significant increase in respiratory rate was documented in any case, in both the groups. No case required stoppage of magnesium infusion or any kind of ventilatory support. Statistically no significant difference was found between respiratory rates (p>0.05).” | - |
|  | Blood pressure | “Similarly no statistical difference was found in relation to mean arterial pressure at any time during the monitoring. No significant fall in blood pressure or mean arterial pressure was seen in any case. No case required dopamine or dobutamine infusion to augment blood pressure, during magnesium infusion” |  |
|  | Side effects | “not associated with any significant side effects.” |  |
| Savitha 2015 | Adverse effects | “No adverse effects related to elevated levels of magnesium were noted in any of the neonates.” | - |
|  | Heart rate, respiratory rate, blood pressure, oxygen saturation | “remained unchanged before and after intervention between two groups.” |  |
|  | Normal cranial ultrasound at 12 months | “for 32 out of 35 infants” (or 41 infants across both groups, included in follow up) |  |
| Siddiqui 2021 | Side effects | “No side effects were noted related to intravenous magnesium sulphate infusion” |  |
| Singh 2015 | Neuroprotective (moderate encephalopathy) | “found neuroprotective…” | Data taken from trial registration report only |
|  | Early control of seizures (moderate encephalopathy) | (p=0.04) |  |
|  | Early appearance of normal cry (moderate encephalopathy) | (p=0.02) |  |
|  | Early appearance of normal activity (moderate encephalopathy) | (p=0.02) |  |
|  | Early acceptance of full oral feed by sucking (moderate encephalopathy) | “p=0.04” |  |
|  | Good short-term outcome (moderate encephalopathy) | “p=0.04” |  |
|  | Neuroprotective (severe encephalopathy) | “not found neuroprotective… (p=>0.05)” |  |
|  | Blood pressure | “No significant differences” |  |
| **MgSO_4_ and TH versus TH alone** | | | |
| Abdel-Aziz 2021 | Respiratory support | “There were statistically significant differences in hypothermia and MS group versus hypothermia and control groups and in hypothermia group versus control group regarding…”  “(p=0.023; p=0.001, and p=0.005, respectively)” | Group data not able to be extracted from figures |
|  | Frequency of convulsions | “(p=0.005; p=0.002, and p=0.002, respectively)” |  |
|  | Initiation of feeding | “(p=0.005; p.0.001, and p=0.008, respectively)” |  |
|  | Antiepileptic drugs at discharge | “(p=0.001; p=0.007, and p=0.031, respectively)” |  |
| Gulczynska 2018 | RBC transfusion “No/patient” | MgSO_4_ and TH (N=38): 0.24; TH alone (N=37): 0.17; P = “NS” | No measures of variance reported |
|  | FFP transfusion “No/patient” | MgSO_4_ and TH (N=38): 0.4; TH alone (N=37): 0.72; P = “NS” |  |
|  | PLT transfusion “No/patient” | MgSO_4_ and TH (N=38): 0.08; TH alone (N=37): 0.14; P = “NS” |  |
|  | Anticonvulsant “No of doses/patient” | MgSO_4_ and TH (N=38): 2.6; TH alone (N=37): 4.4; P = 0.0025 |  |
|  | “mean heart rate and mean arterial blood pressure values” (36 points of measurement over cooling and rewarming; and averaged values over each day) | “In the study group (TH+Mg) there was a slight decrease in heart rate (by 7.6%) and mean arterial pressure (by 7.2%) on the first day of the study, but the difference between the groups was not statistically significant and of minor clinical significance.” | Group data not able to be extracted from figures |
|  | bradycardia or hypotension | “magnesium sulfate… does not significantly increase the occurrence of…” |  |
|  | Use of vasopressors | “magnesium was not associated with an increased need…” |  |
|  | FiNO and MAP | “were also not different between the groups.” |  |
|  | mean platelet count | “in the group receiving magnesium sulfate was slightly lower than in controls, but the difference was not statistically significant” |  |
|  | HIE scores (Thompson scale) (daily during cooling and rewarming) | “On day 5 of the study after the cooling procedure was completed the score was significantly lower in the treatment group (4.75 vs. 7.03; p-value 0.03).” |  |
| Kumar 2022 | Days of antiseizure therapy (median, IQR) | MgSO_4_ and TH (N=67): 3 (2-4); TH alone (N=67): 3 (2-4); P = 0.73 | For oxidative stress markers, unclear if means reported (and no measure of variance) |
|  | Time to attain full oral feeding (median, IQR) | MgSO_4_ and TH (N=67): 5 (5-8); TH alone (N=67): 6 (5-7); P = 0.86 |  |
|  | Median duration of ventilation | “No difference could be found… between the groups (p = 0.58)” |  |
|  | Oxidative stress markers (malondialdehyde and total antioxidant status) | “The serum level of malondialdehyde decreased significantly after 72 h in both the groups. However, the decline in malondialdehyde and increase in total antioxidant status were comparable.”  Serum malondialdehyde 72 hours (nmol/mL): MgSO_4_ and TH (N=unclear): 3.19; TH alone (N=67): 3.01; P = 0.57  Serum total antioxidant status at 72 hours (U/mL): MgSO_4_ and TH (N=unclear): 3.60; TH alone (N=67): 3.25; P = 0.46 |  |
| **MgSO_4_ and melatonin versus melatonin alone** | | | |
| El Farargy 2020 | S100-B | “group 1 [MgSO_4_and melatonin] had lower concentrations of S100-B at 2 days (median = 8 vs 12, p = 0.001) and at 6 days (median = 3 vs 10.5, p < 0.001), respectively” | Data taken from abstract only |

Abbreviations: aEEG: amplitude integrated electroencephalography; CSF: cerebrospinal fluid; FFP: frozen fresh plasma; FiNO: fraction of inspired nitric oxygen; HIE: hypoxic ischaemic encephalopathy; IL-1-β: interleukin-1 beta; IL-6: interleukin-6; INR mg/kg: milligrams per kilogram; IQR: interquartile range; MAP: mean arterial pressure; MgSO_4_: magnesium sulphate; mmHg: millimetres of mercury; N: number of participants; NIBP: non-invasive blood pressure; NS: non-significant; pg/mL: picograms per millilitre; PLT: platelet; RBC: red blood cell; TH: therapeutic hypothermia.
