# Supplementary material for: Neonatal magnesium sulphate for neuroprotection: A systematic review and meta‐analysis
Source: Dev Med Child Neurol. 2024 Mar 11;66(9):1157–72. doi: 10.1111/dmcn.15899 (PMC11579813; doi:10.1111/dmcn.15899)
Supplement: Supplementary file 13 — Table S7: Results from non‐randomized studies [file DMCN-66-1157-s003.docx]

**Table S7:** Results from non-randomised studies

| **Study** | **Participants** | **Relevant comparison groups** | **Outcome measure(s)** | **Result(s)** | **Notes** |
| --- | --- | --- | --- | --- | --- |
| Levene 1995 | N = 15 | 1: MgSO_4_ 400 mg/kg (N=7)  2: MgSO_4_ 250 mg/kg (N=8) | MAP, respiratory depression, EEG readings, heart rate | Group 1: “After the larger dose, mean arterial pressure (MAP) fell by a mean of 6 mm Hg (13%) at one hour but was not significantly reduced thereafter. Respiratory depression lasted three to six hours. EEG readings and heart rate were not significantly different.”  Group 2: “After 250 mg/kg MgSO4, MAP, EEG, tone and heart rate were unchanged. One infant developed transient respiratory depression.” | MAP reported in figures |
| Okonkwo 2018 | N = 711 | 1: MgSO_4_ (N=100)  2: No MgSO_4_ (N=611) | Neonatal death, all | 17/100 vs. 174/611 (χ^2^= 5.762; p = 0.016) |  |
|  |  |  | Neonatal death, neonates with HIE | 15/78 vs. 173/414 (χ^2^ = 14.144; p < 0.0001) |  |
|  |  |  | Neonatal death, neonates with stage III HIE | 10/25 vs. 116/184 (χ^2^= 4.881; p = 0.027) |  |
| Sreenivasa 2017 | N = 100 | 1: MgSO_4_ (N=50)  2: Control (N=50) | Neonatal death | 3/50 vs. 4/50 (p > 0.05) | Likely error in reported P value reported for ‘normal neuromotor tone’ |
|  |  |  | Seizures | 27/50 vs. 29/50 (p > 0.05) |  |
|  |  |  | Seizures controlled with 1 anticonvulsant | 26/50 vs. 21/50 (p < 0.05) |  |
|  |  |  | Seizures continued at 24 hours | 5/50 vs. 10/50 (p < 0.01) |  |
|  |  |  | Duration of recovery from neurological abnormalities (days; mean, SD) | 3.56 ± 1.23 vs. 5.01 ± 1.62 (p < 0.001) |  |
|  |  |  | Initiation of nasogastric tube feeding (days; mean, SD) | 3.25 ± 1.12 vs. 4.02 ± 1.03 (p < 0.001) |  |
|  |  |  | Initiation of spoon feeding (days; mean, SD) | 3.90 ± 1.26 vs. 5.80 ± 1.41 (p < 0.001) |  |
|  |  |  | Initiation of direct breast feeding (days; mean, SD) | 4.90 ± 1.56 vs. 6.30 ± 1.67 (p < 0.001) |  |
|  |  |  | Abnormal neurological findings at discharge | 12/50 vs. 22/50 (p < 0.01) |  |
|  |  |  | Normal neuromotor tone (Amiel-Tison criteria) | 35/50 vs. 19/50 (p > 0.05) |  |
|  |  |  | Normal neuroimaging | 34/50 vs. 19/50 (p < 0.05) |  |
|  |  |  | Heart rate, oxygen saturation, respiratory rate | “showed no significant fall after magnesium administration.” |  |
|  |  |  | Adverse effects | “No adverse effects of magnesium were noted in this study” |  |
| Szemraj 2005 | N = 45 | 1: MgSO_4_ (N=18)  2: Control 1 (no MgSO_4_) (N=7)  3: Control 2 (healthy) (N=20) | ATPase activities in erythrocyte membranes; protein kinases A and C in erythrocyte membranes; immunocharacteristics of band 3 in erythrocyte membranes | Summary: “Results: The time-dependent decrease of Ca^2+^-ATPase activity was detected in untreated newborns, whereas MgSO4 prevented this reduction. After 48 h, protein kinases activities differed in MgSO4-treated and untreated groups. Magnesium therapy increased the amount of band 3 and diminished proteolytic degradation of this protein… These data may partly explain the molecular mechanisms of MgSO4 action in asphyxiated newborns.” | Results presented for individual outcomes in figures |
|  |  |  | Side effects | “but the side effects of MgSO4 administration were not observed during the study” |  |

Abbreviations: ATPase: P-type adenosine triphosphatase; Ca^2+^: calcium cation; EEG: electroencephalogram; HIE: hypoxic ischaemic encephalopathy; MAP: mean arterial pressure; MgSO_4_: magnesium sulphate; mmHg: millimetres of mercury; mg/kg: milligrams per kilogram; N: number of participants; SD: standard deviation.
